# Global, regional, and national disease burden attributable to high body mass index in youth and young adults: 2021 global burden of disease study analysis

**DOI:** 10.3389/fpubh.2025.1680402

**Published:** 2025-11-17

**Authors:** Yushi Zhang, Rongchen Ma, Yue Ma, Renyi Zhou

**Affiliations:** 1 Department of Orthopedics, The First Hospital of China Medical University, Shenyang, China; 2 Department of Neurology, First Hospital of China Medical University, Shenyang, Liaoning, China; 3 Department of Pulmonary and Critical Care Medicine, Shengjing Hospital of China Medical University, Shenyang, China

**Keywords:** disability-adjusted life years, mortality rate, global burden of disease, high BMI, adolescents, young adults

## 1 Background

In recent years, BMI has been globally recognized and widely adopted as an indicator for assessing an individual's height and weight. Specifically, it is calculated by dividing body weight (kg) by the square of height (m) (1). Among adults, a BMI ≥25 kg/m^2^ is defined as overweight, whereas a BMI ≥30 kg/m^2^ falls into the category of obesity (2). A high BMI (i.e., BMI ≥25 kg/m^2^) is a major risk factor for cardiovascular diseases, diabetes, chronic kidney disease, and other conditions (3). It ranks among the top five risk factors for morbidity and mortality, and among younger populations, high BMI is one of the three most prevalent chronic diseases in adolescents (4). Since 1980, obesity rates have doubled in more than 70 countries and have continued to rise in most other nations (5). Recent statistics indicate that overweight/obesity continues to increase globally, with the number of overweight individuals reaching 2 billion, accounting for 30% of the world's population (6). If current trends persist, by 2030, an estimated 38% of the world's adult population will be overweight, with an additional 20% classified as obese (7). When focusing on the adolescent population, overweight and obesity are also prevalent issues within this group. Recent estimates show that over 40 million children and more than 330 million adolescents are overweight or obese (8), and this rate continues to rise. When we focus on the 15–39 age group, the prevalence characteristics of high BMI in this group are highly consistent with the global growth trend. The heterogeneity of risk exposure (such as metabolic sensitivity, behavioral patterns, and socio-economic driving factors) in this group is more prominent compared to other age groups. Revealing the biological mechanisms and social determinants of the dynamic evolution of BMI in this group, and exploring targeted early intervention strategies, have significant public health value in curbing the trend of younger onset of chronic diseases.

As the high BMI rate among these groups increases, they also impose many potential burdens on global healthcare. The substantial increase in overweight and obese populations has imposed significant potential burdens on global health care. Studies indicate a strong association between high BMI and chronic diseases such as cardiovascular disease, diabetes, and cancer. Obesity leads to the accumulation of visceral fat, which secretes proinflammatory cytokines (e.g., IL-6 and TNF-α) and free fatty acids, triggering low-grade systemic inflammation and insulin resistance. This subsequently results in dyslipidaemia (elevated triglycerides and reduced HDL cholesterol), hypertension (sympathetic nervous system activation and renin–angiotensin system dysfunction), and increased risk of atherosclerosis (9). Hypertriglyceridaemia (men: triglycerides >2 mmol/L; women: triglycerides >1.5 mmol/L) can serve as a simple screening indicator for excessive visceral fat, and this phenotype is significantly correlated with coronary heart disease risk. Consequently, obese individuals (BMI ≥30 kg/m^2^) are at markedly greater risk of mortality than are those with a normal weight (10). The research focus was placed on the 15–39 age group. It was found that a high BMI not only poses an immediate threat to the metabolic health of this group, but also, through epigenetic imprints and behavioral patterns, solidifies into a cross-generational health effect, significantly increasing the cumulative risks of metabolic syndrome in adulthood and cardiovascular events in old age. Longitudinal studies have revealed that severe obesity (BMI ≥35 kg/m^2^) during adolescence is strongly associated with cardiomyopathy risk in adulthood. Data from a Swedish male cohort revealed that severely obese individuals faced a 9.2-fold greater risk of hospitalization for heart failure (HR = 9.2, 95% CI: 4.2–20.3) (11). The underlying mechanism involves obesity-induced lipid deposition in cardiomyocytes, mitochondrial dysfunction, and inflammatory responses, leading to cardiac remodeling (left ventricular hypertrophy) (12).

When we focus on diabetes, we can always find a relationship between high BMI and it. Each five-unit increase in BMI increases the risk of type 2 diabetes (T2DM) by 72% (RR = 1.72, 95% CI: 1.65–1.81), whereas central obesity indicators (e.g., waist circumference and waist-to-hip ratio) also exhibit a linear positive correlation with diabetes risk (RR = 1.61–1.73) (13). The prevalence of obesity has nearly doubled since 1980, with adult obesity rates reaching 13% in 2014, and childhood obesity poses a similarly severe challenge. The age group of 15–39 years old, which serves as a crucial transitional period from childhood to adulthood, plays a pivotal role in interrupting the intergenerational transmission of obesity and preventing the occurrence of chronic diseases in adults. Targeted research on this group can precisely identify the early warning signs of abnormal BMI trajectories, providing empirical evidence for the formulation of obesity prevention and control plans covering the entire life cycle. The term “diabesity” has been proposed, highlighting that ~80% of diabetic patients are overweight or obese, with the combined effect increasing mortality risk sevenfold (14). Researchers analyzed data from PubMed, Scopus, and Web of Science up to May 2021, incorporating 216 cohort studies covering 26 million participants, and confirmed a robust linear positive correlation between BMI and T2DM risk across all regions, ethnicities, and sexes (15). A 5%−10% weight reduction in obese individuals can lower glycated hemoglobin (HbA1c) by 0.6%−1%, improving diabetes control (9), further substantiating the role of high BMI in diabetes pathogenesis.

At the same time, the occurrence of tumors is also closely related to a high BMI. Obesity is an independent risk factor for various cancers, including breast, endometrial, colorectal, and pancreatic cancer. U.S. data indicate that obese individuals face a sevenfold higher cancer mortality rate than do individuals with normal weight (16). The mechanisms by which high BMI promotes tumorigenesis include (1) adipose tissue inflammation (e.g., macrophage infiltration and crown-like structure (CLS) formation), resembling chronic wound healing, which releases growth factors (VEGF and EGF) to sustain cell proliferation, angiogenesis, and stromal remodeling, creating a “fertile ground” for tumor development (17), and (2) hypoxia in adipose tissue, which induces HIF-1α expression and promotes fibrosis factors (e.g., collagen VI, IL-6, and TNF-α), further fostering a protumor microenvironment (18). Additionally, obesity induces adipose tissue remodeling, where excessive nutrients cause white adipose tissue (WAT) hypertrophy and hyperplasia, triggering endoplasmic reticulum stress, oxidative stress, and the activation of proinflammatory pathways (e.g., NF-κB and JNK). This releases inflammatory cytokines (e.g., IL-6 and TNF-α), while M2 macrophages secrete IL-10 and TGF-β, suppressing antitumour immunity (19).

A high BMI not only facilitates tumor initiation but also impairs lymphatic function (e.g., obesity-related fatty acids such as palmitate disrupt lymphatic tight junctions), promoting lymphedoema and tumor metastasis (20). Prognostic analyses of several cancers revealed that obese (BMI ≥30) patients with breast cancer at diagnosis face a 33% higher mortality risk (HR = 1.33), obese patients with prostate cancer exhibit a 24% increased risk of biochemical recurrence post-surgery (HR = 1.24), and metastatic prostate cancer mortality is further elevated. Extremely obese (BMI ≥35) patients with colon cancer experience 25%−35% shorter overall survival, likely due to exacerbated insulin resistance and inflammation (21).

When we focus on the group of teenagers and young adults (aged 15–39), we can observe that the impact of high BMI on this group presents certain unique characteristics. Adolescents are in a rapid phase of growth and development, with significantly increased energy demands, and the food they consume is metabolized at a faster rate (22). If energy intake consistently exceeds expenditure, it can easily lead to fat accumulation. Concurrently, hormonal changes during adolescence may influence fat distribution, with females being more prone to central obesity (23). Among these adolescents, ~10%−25% are classified as “metabolically healthy obese” (MHO), exhibiting insulin sensitivity comparable to that of normal-weight individuals and lower carotid intima-media thickness (CIMT). However, long-term follow-up studies indicate that MHO adolescents still face a greater risk of developing type 2 diabetes and cardiovascular diseases later in life than do metabolically healthy normal-weight individuals (24).

When investigating the causes of high BMI in adolescents, researchers have reported that monogenic obesity (e.g., MC4R mutations) and common genetic variants (e.g., the FTO gene) exert more pronounced effects during adolescence, possibly due to accelerated energy metabolism during puberty. Additionally, obesity can lead to low self-esteem, increased risk of depression, and impaired social relationships and educational attainment. The psychological stress experienced by obese adolescents may further exacerbate unhealthy lifestyle choices (25).

Furthermore, obesity during adolescence can have significant long-term consequences in adulthood. On the one hand, a high BMI can disrupt hormonal balance, interfering with normal pubertal development. Pubertal sex hormones (e.g., estrogen and testosterone) regulate fat distribution and appetite, influencing fat accumulation in adulthood. Females, whose adipose tissue secretes more leptin and oestradiol, are more susceptible to insulin resistance and weight gain (26). Adolescent obesity not only affects pubertal development but also significantly increases the risk of adult-onset conditions such as gynecological disorders (e.g., PCOS and menstrual abnormalities), mental health issues, and reproductive system cancers through mechanisms such as metabolic dysregulation, endocrine disruption, and chronic inflammation (27).

On the other hand, a high BMI severely impacts the circulatory and metabolic systems. Studies have revealed that individuals who are obese (BMI ≥35) at age 18 face a 42% increased risk of diabetes in adulthood (*P* <0.01), with the risk increasing with increasing severity of obesity. With respect to hypertension, females presented a 25% increased risk (average age of 46 years), whereas males presented a 42% increased risk (*P* <0.01), with interactions observed between sex and age (28). Consequently, obese adolescents are at significantly elevated risk of developing cardiovascular diseases in adulthood, including atherosclerosis and coronary heart disease, as well as nonalcoholic fatty liver disease (NAFLD), which may progress to nonalcoholic steatohepatitis (NASH) or even cirrhosis (29).

Thus, adolescent obesity poses a substantial future health care burden. Early intervention targeting high BMI in adolescents could alleviate future medical pressures and improve overall efficiency. Currently, researchers focus predominantly on analyzing the impact of high BMI across all age groups, often overlooking the unique vulnerabilities of adolescents. This study aims to assess the burden of high BMI among individuals aged 15–39 years across 204 countries and territories worldwide, with the goal of enhancing the understanding and awareness of adolescent overweight and obesity. The choice of the age group of 15–39 years old is also because this age stage is a critical window period for the formation of behavioral habits. BMI intervention at this stage can bring lifelong health benefits. For instance, weight loss during adolescence can significantly reduce the risk of type 2 diabetes in adulthood (30). BMI in this age range is closely related to career development and fertility, which has unique public health economic significance.

## 2 Method

### 2.1 Data sources and integration

Dataset: based on the open database of the Global Burden of Disease Study 2021 (GBD 2021), it contains health indicator data for 204 countries/regions from 1990 to 2021.

Ethical compliance: a completely anonymized dataset was used. The research plan was approved by the institutional review board and complies with the ethical guidelines of the Helsinki Declaration.

Data calibration: a stratified model, space-time modeling, and Bayesian inference were employed to calibrate cross-regional heterogeneity, ensuring the comparability of indicators for countries at different levels of development (31).

### 2.2 Research subjects and exposure definition

Population scope: inclusion of adolescents and young adults aged 15–39 worldwide, divided into five groups according to the GBD 2021 age classification criteria (15–19, 20–24, 25–29, 30–34, 35–39 years old).

High BMI definition: BMI ≥ 25 kg/m^2^ (overweight) or ≥ 30 kg/m^2^ (obese), using the updated WHO standard of 2023.

Theoretical minimum risk exposure (TMREL): Set BMI = 20–25 kg/m^2^ as the theoretical risk-free exposure range, and calculate the state with the minimum disease burden within this range (5).

### 2.3 Statistical analysis methods

#### 2.3.1 Nonlinear correlation modeling

##### 2.3.1.1 Gaussian process regression (GPR)

Core principle: based on the Bayesian framework, the covariance structure between the input space (SDI) and the output variable (BMI burden index) is defined through the kernel function to generate a probabilistic prediction surface.

Advantage comparison: compared to the generalized additive model (GAM), GPR does not require presetting the function form and can automatically identify the non-monotonic correlation between BMI and SDI (such as *U*-shaped curve); compared with spline regression, it has stronger generalization ability for small sample data.

#### 2.3.2 Subgroup stratification and trend analysis

Gender stratification: separate models were built for males and females, controlling for the modifying effects of sex hormone levels on the association between BMI and diseases (such as the impact of androgens on the distribution of visceral fat in males).

Age stratification: the 15–39 age group was divided into five independent groups for analysis, capturing the differences in metabolic trajectories between adolescence (15–19 years old) and early adulthood (20–39 years old).

Regional stratification: ecological analysis was conducted by geographical regions of the 21 GBD categories to identify regional heterogeneity in SDI and BMI burden (such as the differences between high-income regions in the Middle East and low-income regions in Sub-Saharan Africa).

#### 2.3.3 Quantification of trends and effects

Estimated annual percentage change (EAPC): the annual average change rate of the BMI burden indicators is calculated through linear regression.

Standardized rate calculation: the direct standardization method based on the global population age structure is used to eliminate the influence of age composition differences on the mortality rate/DALY rate.

#### 2.3.4 Visualization and spatial analysis

Map generation: using the R language's ggplot2 package (version 4.3.2), a distribution map of BMI-related mortality/DALY rates for 204 countries was created, with the color gradient representing the interquartile range of the standardized rates at 49.

Spatial clustering: the K-means algorithm was employed to identify disease burden hotspots.

Correlation visualization: a Spearman correlation heat map of SDI and BMI burden indicators was drawn.

### 2.4 Construction of multivariate model

Variable adjustment: gradually incorporate potential confounding factors such as age, gender, and per capita GDP into the main effect model of SDI, and use backward stepwise regression to select variables.

Independent effect test: calculate the marginal effect of SDI after adjusting for age and gender through stratified analysis.

### 2.5 Ethical statement

The data was obtained from the GBD open platform (https://vizhub.healthdata.org/gbd-compare/). All analyses were conducted on encrypted servers and strictly followed the data usage protocol. The study did not involve individual identity information and met the data anonymization standards of CRDCN.

## 3 Results

### 3.1 Overall high-BMI-related burden in youth and young adults

**Figure 1:**
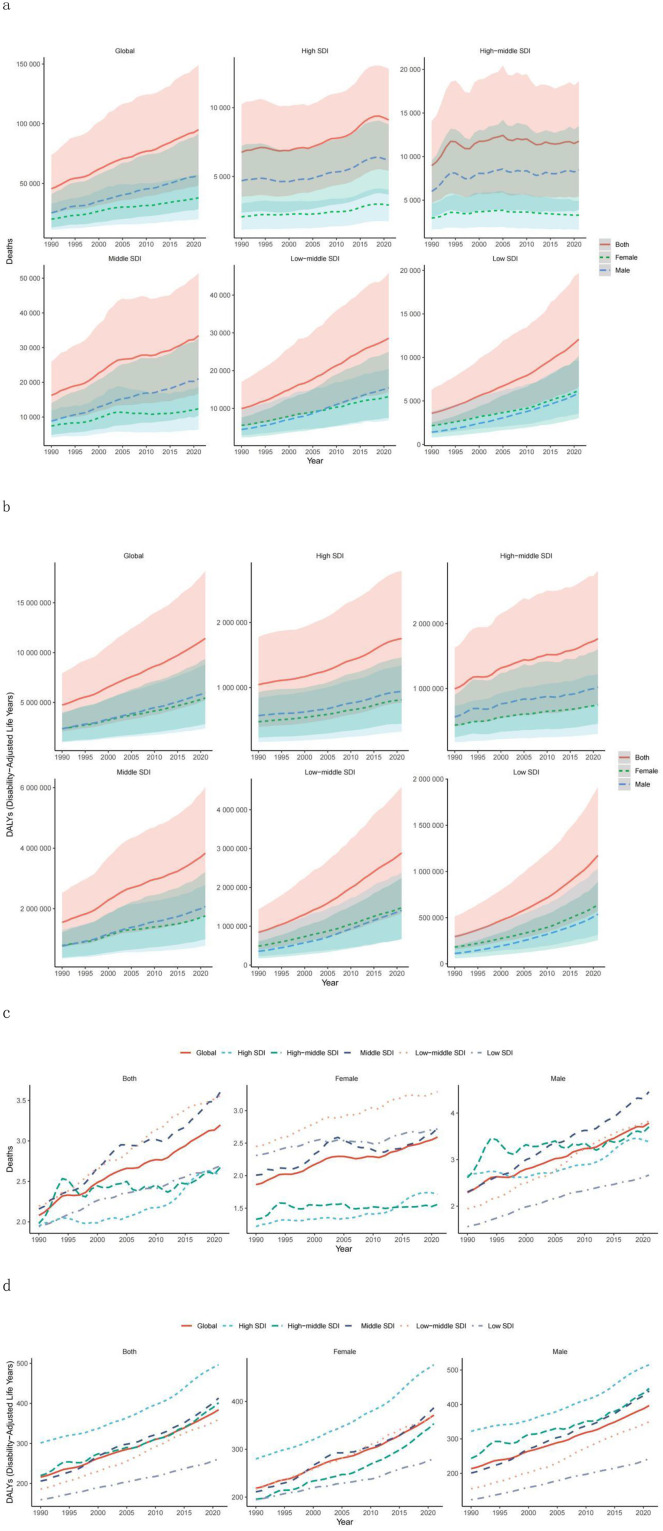
Temporal trends in global disease burden attributable to high BMI among youth and young adults by SDI quintile and sex from 1990–2021. **(a)** Number of deaths. **(b)** DALYs. **(c)** Mortality rate. **(d)** DALY rate. DALY, disability-adjusted life year; SDI, sociodemographic index.

Globally, the number of deaths and DALYs associated with high BMI among adolescents and young adults has shown a continuous upwards trend, increasing from 455,789 (95% UI: 24,580.60–73,819.16) in 1990 to 951,102 (95% UI: 48,227.30–148,807.26) in 2021, indicating a 53.73% increase (95% UI: 38.40%−68.90%; Table 1; Figure 1a; Supplementary Table S1). DALYs increased from 4,733,088 (95% UI: 2,084,809.24–7,910,759.74) in 1990 to 11,428,432 (95% UI: 5,195,837.94–18,184,470.87) in 2021, reflecting 77.9% growth (95% UI: 62.00–94.03%; Table 2; Figure 1b; Supplementary Table S2).

**Table 1:** Number of deaths, mortality rates, and EAPCs among youths and young adults with high BMIs (1990–2021).

**Location**	**Number of deaths (95% UI)**		**Mortality rate/100,000 (95% UI)**		**EAPC of mortality rate (95% CI)**	**Age-standardized mortality rate/100,000 (95% UI)**		**EAPC of age-standardized mortality rate (95% CI)**	**Difference in number of cases (%, 95% UI)**
	**1990**	**2021**	**1990**	**2021**	**1990–2021**	**1990**	**2021**	**1990–2021**	**1990–2021**
Global	45,579.84 (24,580.60–73,819.16)	95,102.93 (48,227.30–148,807.26)	2.08 (1.12–3.37)	3.20 (1.62–5.00)	2.29 (2.19–2.40)	2.25 (1.18–3.66)	3.10 (1.59–4.83)	1.00 (0.95–1.04)	53.73 (38.40–68.90)
**SDI region**									
High SDI	6,758.07 (3,522.45–10,230.39)	9,109.90 (5,424.26–12,819.17)	1.95 (1.02–2.95)	2.58 (1.54–3.63)	1.10 (0.92–1.27)	1.83 (0.94–2.77)	2.24 (1.31–3.17)	0.85 (0.70–1.00)	32.41 (15.68–56.00)
High–middle SDI	8,964.14 (4,661.09–14,081.90)	11,770.34 (5,404.16–18,597.26)	1.98 (1.03–3.11)	2.67 (1.23–4.22)	0.38 (0.14–0.62)	2.00 (1.03–3.18)	2.24 (1.04–3.52)	−0.04 (−0.20 to 0.12)	34.96 (18.23–50.83)
Middle SDI	16,253.28 (8,877.95–25,942.59)	33,460.26 (16,799.23–51,475.08)	2.16 (1.18–3.45)	3.61 (1.81–5.55)	2.22 (2.02–2.43)	2.46 (1.34–3.95)	3.35 (1.70–5.15)	1.00 (0.93–1.06)	67.06 (46.13–84.72)
Low–middle SDI	9,955.03 (5,241.47–17,014.72)	28,569.87 (14,330.41–45,817.91)	2.20 (1.16–3.75)	3.56 (1.79–5.71)	3.56 (3.44–3.68)	2.55 (1.31–4.38)	3.74 (1.91–6.00)	1.37 (1.28–1.46)	62.15 (40.29–88.92)
Low SDI	3,578.50 (2,123.08–6,264.65)	12,088.71 (6,609.95–19,691.47)	1.94 (1.15–3.40)	2.69 (1.47–4.39)	3.95 (3.88–4.03)	2.32 (1.31–4.15)	3.21 (1.71–5.27)	0.95 (0.87–1.02)	38.65 (15.74–65.97)
**GBD region**									
Andean Latin America	523.60 (238.88–988.99)	928.26 (477.30–1,487.77)	3.39 (1.54–6.40)	3.43 (1.76–5.49)	1.69 (1.52–1.85)	3.94 (1.79–7.35)	3.44 (1.74–5.55)	−0.55 (−0.72 to −0.39)	1.24 (−22.38 to 28.29)
Australasia	119.73 (56.68–187.90)	125.20 (61.86–182.87)	1.47 (0.70–2.30)	1.20 (0.59–1.75)	−0.29 (−0.50 to −0.08)	1.42 (0.66–2.28)	1.07 (0.53–1.59)	−1.10 (−1.31 to −0.89)	−18.57 (−28.79 to −8.46)
Caribbean	565.92 (303.47–830.32)	1,016.90 (547.83–1,523.17)	3.81 (2.04–5.59)	5.59 (3.01–8.37)	1.96 (1.78–2.14)	4.34 (2.33–6.53)	5.50 (3.07–8.38)	1.09 (0.83–1.35)	46.74 (24.76–73.97)
Central Asia	917.97 (440.25–1,521.27)	1,410.16 (705.99–2,307.01)	3.23 (1.55–5.35)	3.77 (1.89–6.17)	0.27 (−0.19 to 0.73)	3.56 (1.72–5.98)	3.48 (1.71–5.63)	−0.97 (−1.46 to −0.47)	16.91 (2.77–36.92)
Central Europe	1,640.15 (766.51–2,624.02)	725.54 (379.46–1,111.94)	3.50 (1.64–5.60)	2.07 (1.08–3.18)	−2.51 (−2.72 to −2.30)	3.16 (1.50–5.07)	1.70 (0.89–2.60)	−2.19 (−2.33 to −2.05)	−40.82 (−47.67 to −29.69)
Central Latin America	2,497.87 (1,275.31–3,785.50)	5,536.35 (3,137.16–7,951.10)	3.66 (1.87–5.55)	5.47 (3.10–7.86)	2.61 (2.32–2.90)	4.31 (2.21–6.58)	5.49 (3.06–7.94)	0.85 (0.48–1.23)	49.57 (31.21–69.93)
Central Sub-Saharan Africa	481.34 (258.30–913.43)	2,014.18 (946.14–3,574.19)	2.32 (1.24–4.40)	3.72 (1.75–6.61)	4.61 (4.54–4.68)	2.83 (1.43–5.56)	4.45 (2.06–8.24)	1.40 (1.34–1.46)	60.60 (17.86–118.93)
East Asia	6,297.85 (3,902.76–9,887.64)	11,504.50 (5,300.42–18,576.84)	1.11 (0.69–1.75)	2.40 (1.11–3.88)	1.68 (1.46–1.89)	1.20 (0.72–1.90)	1.99 (0.93–3.24)	1.60 (1.39–1.80)	115.72 (45.21–179.59)
Eastern Europe	25,90.74 (1,166.20–4,212.71)	2,581.85 (1,048.87–4,248.36)	3.02 (1.36–4.91)	3.90 (1.59–6.42)	−0.97 (−1.55 to −0.39)	2.68 (1.19–4.31)	2.95 (1.21–4.94)	−0.57 (−1.30 to 0.16)	29.17 (7.72–50.61)
Eastern Sub-Saharan Africa	1,547.76 (1,006.30–2,555.35)	4,612.01 (2,691.47–7,549.38)	2.18 (1.42–3.60)	2.63 (1.54–4.31)	3.40 (3.32–3.48)	2.70 (1.62–4.41)	3.18 (1.78–5.26)	0.27 (0.18–0.36)	20.58 (−4.42 to 49.63)
High-income Asia Pacific	634.71 (400.47–958.58)	287.97 (152.75–435.86)	0.94 (0.59–1.42)	0.57 (0.30–0.86)	−2.78 (−2.92 to −2.65)	0.93 (0.57–1.43)	0.49 (0.26–0.74)	−2.47 (−2.61 to −2.34)	−39.41 (−52.50 to −25.83)
High-income North America	3,010.13 (1,588.03–4,364.42)	4,218.47 (2,751.91–5,477.12)	2.66 (1.40–3.85)	3.42 (2.23–4.45)	1.06 (0.90–1.21)	2.42 (1.27–3.55)	3.19 (2.08–4.18)	1.07 (0.93–1.21)	28.92 (11.68–61.22)
North Africa and Middle East	6,418.84 (3,388.12–10,134.24)	15,761.53 (8,386.75–23,651.04)	4.80 (2.53–7.57)	6.20 (3.30–9.30)	3.16 (3.07–3.26)	5.70 (2.94–9.04)	5.91 (3.15–8.92)	0.18 (0.09–0.27)	29.24 (10.57–56.78)
Oceania	201.60 (96.33–333.17)	541.35 (262.82–872.46)	7.59 (3.63–12.54)	9.61 (4.66–15.48)	3.15 (3.03–3.27)	9.01 (4.23–15.07)	10.35 (4.99–16.74)	0.38 (0.31–0.46)	26.60 (2.01–61.28)
South Asia	6,095.50 (2,735.16–11,624.85)	20,398.17 (9,472.39–34,561.94)	1.41 (0.63–2.69)	2.58 (1.20–4.37)	4.11 (3.80–4.43)	1.61 (0.71–3.24)	2.67 (1.20–4.50)	1.80 (1.53–2.06)	82.62 (47.10–128.19)
Southeast Asia	4,307.84 (2,409.03–7,180.52)	10,625.81 (5,350.67–16,920.44)	2.19 (1.22–3.64)	3.83 (1.93–6.10)	2.83 (2.53–3.13)	2.49 (1.36–4.07)	3.71 (1.91–5.92)	1.28 (1.09–1.47)	75.22 (44.60–111.76)
Southern Latin America	514.27 (235.98–854.69)	534.97 (273.02–809.80)	2.70 (1.24–4.48)	2.07 (1.06–3.14)	0.37 (0.14–0.59)	2.81 (1.28–4.70)	1.99 (1.00–3.04)	−0.89 (−1.10 to −0.68)	−23.06 (−32.94 to −10.81)
Southern Sub-Saharan Africa	1,332.66 (655.17–2,387.19)	2,307.30 (1,141.22–4,048.67)	6.17 (3.03–11.04)	6.78 (3.35–11.90)	1.39 (0.19–2.61)	7.37 (3.56–13.40)	6.60 (3.23–11.81)	−0.59 (−1.79 to 0.61)	9.95 (−5.19 to 27.84)
Tropical Latin America	2,444.93 (1,268.36–3,752.03)	3,237.64 (1,587.42–4,745.86)	3.80 (1.97–5.83)	3.67 (1.80–5.37)	0.74 (0.60–0.87)	4.25 (2.25–6.60)	3.38 (1.68–4.97)	−0.92 (−1.09 to −0.75)	−3.56 (−14.41 to 7.14)
Western Europe	1,907.45 (901.92–008.24)	1,004.05 (498.52–1,502.17)	1.32 (0.63–2.09)	0.77 (0.38–1.16)	−2.17 (−2.31 to −2.03)	1.28 (0.61–1.99)	0.69 (0.35–1.04)	−1.95 (−2.03 to −1.88)	−41.54 (−46.40 to −36.25)
Western Sub-Saharan Africa	1,528.99 (938.10–2,601.71)	5,730.72 (3,177.02–8,891.09)	2.14 (1.31–3.63)	3.00 (1.66–4.65)	4.27 (4.07–4.46)	2.59 (1.49–4.37)	3.63 (2.03–5.70)	1.00 (0.83–1.16)	40.30 (9.53–78.38)

**Table 2:** DALYs, DALY rates, and EAPCs of youths and young adults with high BMIs (1990–2021).

**Location**	**DALYs (95% UI)**		**DALY rate/100,000 (95% UI)**		**EAPC of DALY rate (95% CI)**	**Age-standardized DALY rate/100,000 (95% UI)**		**EAPC of age-standardized DALY rate (95% CI)**	**Difference in number of cases (%- 95% UI)**
	**1990**	**2021**	**1990**	**2021**	**1990–2021**	**1990**	**2021**	**1990–2021**	**1990–2021**
Global	47,33,088.15 (20,84,809.24–79,10,759.74)	11,428,432.13 (51,95,837.94–18,184,470.87)	215.95 (95.12–360.93)	384.17 (174.66–611.28)	1.83 (1.80–1.85)	231.29 (103.06–386.11)	373.72 (169.66–591.85)	1.58 (1.56–1.60)	77.90 (62.00–94.03)
**SDI region**									
High SDI	10,44,695.94 (394,887.42–17,73,710.69)	17,53,422.03 (767,238.56–2,789,234.06)	301.10 (113.81–511.21)	496.38 (217.20–789.61)	1.64 (1.55–1.72)	285.32 (107.12–488.73)	445.61 (191.62–719.66)	1.56 (1.49–1.62)	64.86 (48.76–89.07)
High–middle SDI	995,060.03 (408,923.58–16,27,943.14)	1,768,661.46 (755,729.87–28,14,994.44)	219.88 (90.36–359.74)	401.72 (171.65–639.38)	1.71 (1.61–1.81)	221.81 (93.07–367.22)	346.82 (146.52–556.59)	1.30 (1.24–1.36)	82.70 (65.77–99.92)
Middle SDI	1,549,630.40 (727,937.80–2,521,715.19)	3,834,480.61 (1,788,927.11–6,015,002.08)	205.89 (96.72–335.05)	413.43 (192.88–648.52)	2.18 (2.10–2.27)	230.76 (109.77–379.47)	388.02 (178.22–606.06)	1.71 (1.66–1.76)	100.79 (79.32–118.45)
Low–middle SDI	842,782.20 (399,227.70–1,432,594.55)	2,886,912.26 (1,342,653.85–45,79,034.76)	185.88 (88.05–315.97)	359.74 (167.31–570.60)	2.23 (2.19–2.27)	212.69 (99.42–368.58)	375.93 (172.91–599.40)	1.97 (1.91–2.02)	93.53 (68.96–120.07)
Low SDI	294,037.74 (156,839.89–513,279.76)	1,173,284.11 (573,379.89–1,911,808.28)	159.54 (85.10–278.49)	261.27 (127.68–425.73)	1.57 (1.54–1.59)	187.26 (97.00–331.55)	306.05 (149.14–499.35)	1.51 (1.48–1.55)	63.77 (39.01–87.38)
**GBD region**									
Andean Latin America	46,131.33 (19,295.85–82,769.46)	104,694.68 (45,845.04–170,268.08)	298.32 (124.78–535.25)	386.62 (169.30–628.77)	0.79 (0.65–0.92)	341.48 (139.62–625.79)	386.51 (167.44–627.26)	0.36 (0.24–0.49)	29.60 (7.07–54.01)
Australasia	27,743.79 (9,140.14–49,980.35)	43,895.99 (13,034.58–79,697.98)	340.25 (112.10–612.96)	419.22 (124.48–761.14)	0.42 (0.22–0.61)	331.67 (104.96–625.77)	387.39 (111.35–718.57)	0.37 (0.18–0.56)	23.21 (3.76–41.62)
Caribbean	56,685.41 (26,590.97–86,156.15)	114,248.71 (59,076.48–170,962.00)	381.34 (178.89–579.60)	627.65 (324.55–939.21)	1.58 (1.48–1.69)	425.44 (199.60–660.96)	618.26 (312.53–933.52)	1.38 (1.25–1.51)	64.59 (48.97–84.62)
Central Asia	85,710.45 (35,108.70–141,184.40)	164,173.06 (71,054.34–266,619.75)	301.23 (123.39–496.19)	439.12 (190.05–713.14)	0.34 (0.04–0.64)	327.52 (135.68–546.40)	406.47 (176.94–660.65)	0.11 (−0.19 to 0.42)	45.78 (30.85–61.79)
Central Europe	172,941.76 (64,447.32–295,193.98)	133,532.99 (46,395.51–233,181.13)	369.15 (137.57–630.10)	381.31 (132.48–665.86)	0.25 (0.05–0.46)	339.15 (125.04–582.50)	323.34 (111.17–564.55)	−0.22 (−0.28 to −0.16)	3.29 (−9.55 to 14.47)
Central Latin America	277,580.28 (125,006.36–436,900.06)	676,831.40 (322,543.12–1,016,488.00)	406.61 (183.11–639.98)	669.05 (318.84–1,004.81)	1.62 (1.47–1.77)	469.60 (213.53–739.16)	670.13 (320.65–1,016.25)	1.21 (1.04–1.37)	64.54 (50.89–79.31)
Central Sub-Saharan Africa	37,353.29 (19,528.55–68,079.89)	1,82,186.94 (83,695.56–321,468.02)	179.91 (94.06–327.90)	336.78 (154.72–594.25)	1.94 (1.88–2.00)	215.15 (105.46–405.92)	395.72 (174.75–694.49)	1.91 (1.86–1.96)	87.20 (44.21–140.92)
East Asia	736,001.83 (399,280.97–1,184,445.61)	1,786,095.57 (820,870.22–2,754,698.24)	130.10 (70.58–209.37)	372.84 (171.35–575.04)	3.37 (3.16–3.59)	138.66 (73.47–222.54)	319.91 (146.27–497.15)	2.87 (2.70–3.04)	186.57 (129.73–227.72)
Eastern Europe	272,906.31 (99,913.48–457,493.60)	298,509.76 (106,960.74–507,019.05)	318.19 (116.49–533.41)	451.10 (161.64–766.20)	0.52 (0.26–0.78)	285.02 (103.67–484.73)	355.46 (124.86–606.72)	0.18 (−0.24–0.62)	41.77 (25.46–58.29)
Eastern Sub-Saharan Africa	120,235.46 (72,518.09–201,570.19)	410,341.69 (201,902.49–669,481.98)	169.61 (102.30–284.34)	234.23 (115.25–382.16)	0.92 (0.84–1.00)	205.37 (118.35–340.55)	277.52 (136.22–454.32)	0.78 (0.71–0.85)	38.10 (11.15–63.53)
High-income Asia Pacific	99,559.21 (45,482.98–161,770.50)	114,927.68 (48,061.98–183,774.04)	147.51 (67.39–239.68)	227.40 (95.10–363.63)	1.41 (1.31–1.50)	146.96 (65.75–246.38)	201.22 (84.11–328.44)	0.93 (0.79–1.07)	54.17 (25.55–82.28)
High-income North America	477,573.27 (166,033.99–833,592.04)	777,178.39 (342,221.36–1,231,271.62)	421.45 (146.52–735.64)	630.91 (277.81–999.54)	1.31 (1.25–1.37)	389.88 (135.11–685.46)	597.29 (255.64–970.09)	1.54 (1.45–1.64)	49.70 (30.28–84.94)
North Africa and Middle East	551,612.16 (246,448.69–890,566.55)	1,771,614.49 (834,009.56–2,752,964.65)	412.18 (184.15–665.45)	696.76 (328.01–1,082.71)	1.85 (1.72–1.99)	480.90 (212.94–775.91)	670.10 (315.77–1,041.36)	1.12 (1.05–1.19)	69.04 (48.39–93.31)
Oceania	158,22.23 (7,493.27–25,267.91)	48,690.64 (24,064.42–74,484.68)	595.63 (282.09–951.21)	864.16 (427.10–1,321.96)	1.05 (0.98–1.12)	693.56 (328.54–1,130.57)	921.24 (453.09–1,426.56)	0.83 (0.78–0.88)	45.08 (23.10–73.21)
South Asia	539,015.66 (231,057.85–972,344.95)	2,177,457.75 (945,173.62–3,514,101.71)	124.88 (53.53–225.28)	275.30 (119.50–444.30)	2.63 (2.51–2.76)	140.27 (59.94–263.96)	283.70 (123.18–464.64)	2.40 (2.25–2.56)	120.45 (83.24–169.99)
Southeast Asia	337,041.75 (173,070.54–550,573.45)	902,330.15 (400,006.14–1,457,368.88)	171.08 (87.85–279.47)	325.37 (144.24–525.51)	1.94 (1.79–2.10)	192.75 (94.47–318.96)	315.44 (142.62–508.78)	1.49 (1.36–1.63)	90.18 (64.48–121.10)
Southern Latin America	60,944.68 (22,393.35–107,504.41)	104,568.85 (37,982.59–177,431.39)	319.43 (117.37–563.46)	405.37 (147.24–687.83)	0.87 (0.75–1.00)	331.15 (121.88–587.57)	390.40 (139.78–666.91)	0.65 (0.56–0.75)	26.91 (8.04–41.56)
Southern Sub-Saharan Africa	99,239.51 (44,242.33–177,666.03)	195,797.43 (88,617.83–341,658.99)	459.12 (204.68–821.95)	575.27 (260.36–1,003.82)	0.49 (−0.34 to 1.32)	540.46 (243.47–980.40)	561.18 (252.16–981.68)	−0.05 (−0.95 to 0.86)	25.30 (9.79–41.70)
Tropical Latin America	227,741.06 (102,100.69–364,665.74)	414,729.45 (167,222.52–666,173.07)	354.11 (158.76–567.02)	469.63 (189.36–754.36)	0.85 (0.71–0.99)	389.93 (170.61–635.04)	438.96 (176.36–718.92)	0.32 (0.22–0.42)	32.62 (12.22–49.79)
Western Europe	357,556.11 (136,993.20–601,954.32)	434,818.38 (176,381.78–725,415.47)	248.10 (95.05–417.68)	335.06 (135.92–558.99)	0.95 (0.90–0.99)	240.35 (89.09–403.18)	309.00 (120.81–526.33)	0.88 (0.82–0.95)	35.05 (21.35–47.82)
Western Sub-Saharan Africa	133,692.57 (71,154.60–223,006.62)	571,808.14 (294,000.47–902,010.83)	186.79 (99.41–311.57)	299.05 (153.76–471.74)	1.49 (1.38–1.61)	222.12 (113.76–378.23)	355.06 (181.40–567.15)	1.45 (1.34–1.56)	60.10 (35.75–89.18)

Concurrently, both the death rate and DALY rate exhibited an upwards trajectory from 1990 to 2021. The death rate rose from 2.08 per 100,000 population (95% UI: 1.12–3.37) in 1990 to 3.20 per 100,000 population (95% UI: 1.62–5.00) in 2021. Similarly, the DALY rate increased from 215.95 per 100,000 population (95% UI: 95.12–360.93) in 1990 to 384.17 per 100,000 population (95% UI: 174.66–611.28) in 2021. Additionally, the age-standardized mortality and DALY rates also demonstrated an increasing trend (Tables 1, 2; Figures 1c, d).

### 3.2 High-BMI-related burden in youth and young adults by GBD region

From 1990 to 2021, most GBD regions presented a significant upwards trend in the number of adolescent and young adult deaths and DALYs associated with high BMI. However, Central Europe, the high-income Asia Pacific region, and Western Europe showed notable declining trends, whereas Eastern Europe demonstrated a slight decrease (Tables 1, 2). In 2021, South Asia recorded the highest number of deaths and DALYs linked to high BMI, whereas Australasia had the lowest.

Additionally, apart from Australasia, Central Europe, Southern Latin America, and Western Europe—where death rates displayed a downwards trend—all other GBD regions experienced varying degrees of increase in high-BMI-related death rates among adolescents and young adults. Central Europe showed the largest decline, whereas Central Latin America had the greatest increase (Tables 1, 2).

In 2021, Oceania had the highest death and DALY rates, with a substantial increase compared with most GBD regions. Conversely, the high-income Asia Pacific region maintained the lowest death and DALY rates, which were consistently lower than those reported in other regions (Tables 1, 2; Supplementary Figure S1).

### 3.3 High-BMI-related burden in youth and young adults by country/territory

In 2021, among the 204 countries and regions analyzed in the GBD study, India had the highest number of deaths, followed by China and Pakistan. In terms of DALYs, China ranked the highest, followed by India and the United States (Supplementary Tables S3, S4).

**Figure 2:**
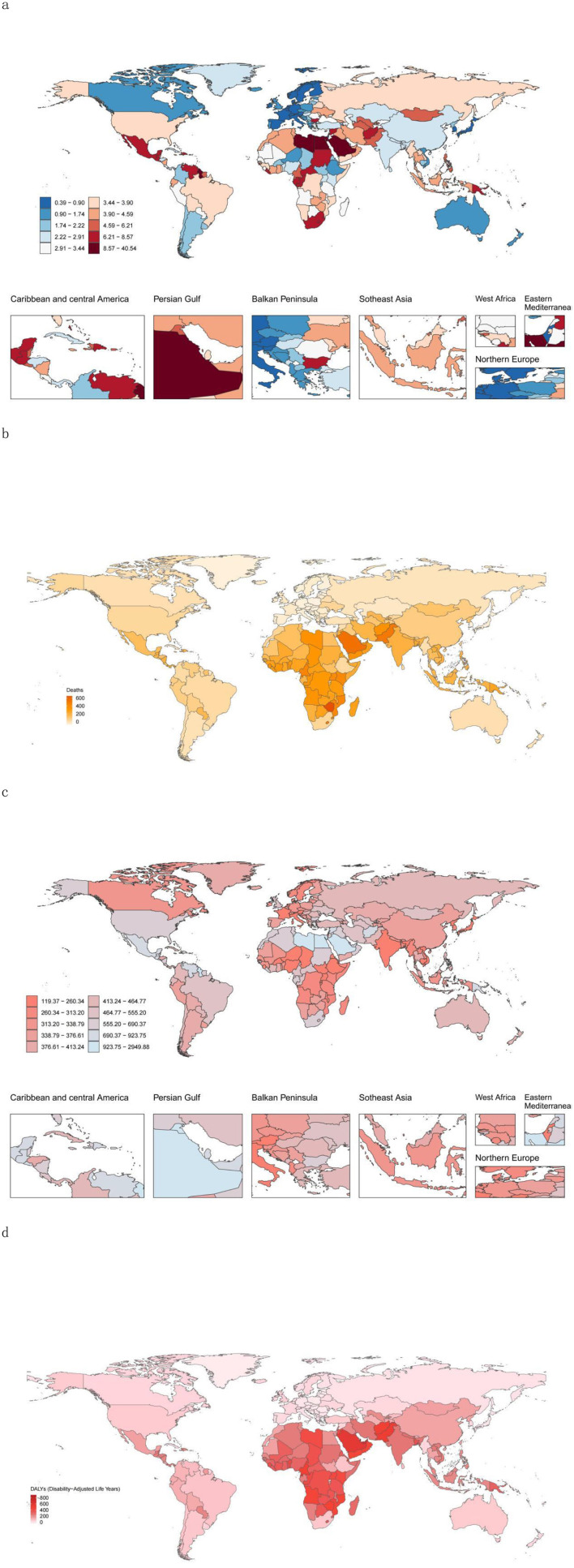
High-BMI-related burden globally in youths and young adults in 204 countries/territories. **(a)** Mortality rate in 2021. **(b)** Change in the number of deaths attributable to High-BMI between 1990 and 2021. **(c)** DALY rate in 2021. **(d)** Change in the DALYs attributable to HSBP between 1990 and 2021. DALY, disability-adjusted life year.

Nauru presented the highest DALY rate and death rate, with the Marshall Islands and Kiribati following closely (Figure 2; Supplementary Tables S3, S4). San Marino recorded the lowest number of deaths, whereas Switzerland had the lowest death rate. Tokelau reported the lowest DALYs, and Vietnam reported the lowest DALY rate. Zimbabwe experienced the largest percentage increase in both the number of deaths and the death rate (Figure 2; Supplementary Tables S3, S4).

Hungary saw the greatest percentage decrease in the number of deaths, whereas Luxembourg had the largest percentage decline in the death rate. The State of Qatar showed the highest percentage increase in DALYs, and Zimbabwe had the largest percentage increase in DALY rates. Conversely, Hungary recorded the greatest percentage reduction in DALYs, and the Federal Democratic Republic of Ethiopia exhibited the largest percentage decrease in DALY rates (Figure 2; Supplementary Tables S3, S4).

Additionally, Zimbabwe demonstrated the most significant increase in the EAPC for both death and DALY rates, whereas Luxembourg had the largest decline in the EAPC for death rates (Supplementary Table S5).

### 3.4 Association of High-BMI-related burden with SDI

The analysis of regions stratified by SDI levels revealed that from 1990 to 2021, both death and DALY rates in SDI regions significantly increased. The increases were more pronounced in the low-SDI, low–middle-SDI, and middle-SDI regions than in the other SDI regions, with high-school-SDI regions showing the most significant increase (Tables 1, 2; Figures 1a, b). Notably, mortality and DALY rates in SDI regions have shown an increasing trend from 1990 to 2021 (Tables 1, 2; Figures 1a, b).

**Figure 3:**
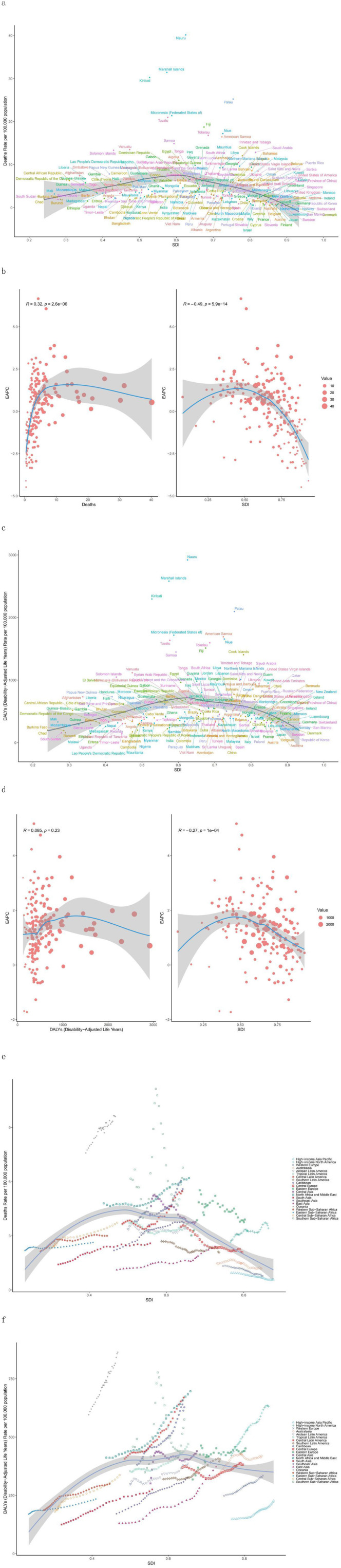
Correlations between high-BMI-related mortality rates and DALY rates and between the SDI and EAPC. **(a**, **c)** Correlations between mortality **(a)** or DALY **(c)** rates and the SDI in 2021. **(b, d)** Correlations between the EAPC of mortality rate **(b)** or DALY rate **(d)** and the SDI in 2021. **(e, f)** Associations between the high-BMI-related death or DALY rate and the SDI (1990–2021) across 21 regions. For each region, the points from left to right depict the estimates for each year. The solid blue line represents the expected values across the SDI spectrum. **(e)** Mortality rate. **(f)** DALY rate. DALY, disability-adjusted life year; EAPC, estimated annual percentage change; SDI, sociodemographic index.

In 2021, the number of deaths and DALYs were highest in medium-SDI areas, followed by low-SDI areas, with the lowest number of deaths and relatively high DALYs in high-SDI areas, and the opposite was true for low-SDI areas (Tables 1, 2); the mortality rate was lowest and the DALY rate was highest in high-SDI areas, with the highest mortality rate in medium-SDI areas and the lowest DALY rate in low-SDI areas (Tables 1, 2; Figure 1). Overall, the relationships between mortality and DALY rates associated with high BMI and SDI were similar to a normal distribution (Figure 3), and the EAPC of death and DALY rates was negatively correlated with the SDI (Figure 3).

When Gaussian process regression was used to analyse the correlation between SDI and mortality and DALY rates, we observed that a higher SDI indicated a higher DALY rate and a lower mortality rate. However, in Southern Sub-Saharan Africa, this relationship markedly differed from that in other regions (Figure 3). The rate rapidly climbed to a peak of ~0.55 before plummeting, while maintaining a relatively high mortality rate. In contrast, the mortality rate in the high-income North America region showed a trend opposite to that of other high-SDI regions. In high-SDI areas, the mortality rates for the high-income Asia Pacific region, Western Europe, and Australasia showed a downwards trend and were significantly lower than those in other SDI regions. Notably, East Asia maintained a moderate SDI while having a low mortality rate (Figure 3e). On the other hand, Oceania, despite having a low SDI, had not only a high mortality rate but also a high DALY rate (Figure 3f).

### 3.5 High-BMI-related burden by sex

Globally, from 1990 to 2021, the mortality rate associated with high BMI among adolescents and young adults increased by 108.65% (95% UI: 87.84%−129.23%), with males experiencing a rise of 124.81% (95% UI: 102.70%−146.21%) and females an increase of 88.30% (95% UI: 65.67%−115.85%; Figure 1a; Supplementary Table S1). Concurrently, the DALYs for males increased by 153.18% (95% UI: 129.52%−176.83%), whereas those for females increased by 129.75% (95% UI: 107.80%−154.51%; Figure 1b; Supplementary Table S2). In 2021, both the number of deaths and DALYs were greater in males than in females, with the death count being nearly 1.5 times greater than that of females, whereas the DALYs showed no significant disparity (Supplementary Tables S1, S2). That same year, the mortality rate and DALY rate were higher in males than in females, with both metrics exhibiting slight increases (Supplementary Tables S1, S2).

From the perspective of different SDI levels, between 1990 and 2021, both the number of deaths and mortality rates increased for males and females across all SDI regions. The middle-SDI regions presented the highest number of deaths and mortality rates, whereas the high-SDI regions presented the lowest number of deaths and mortality rates. Notably, only in low-SDI regions did females have higher numbers of deaths and mortality rates than males did (Supplementary Tables S1, S2, Supplementary Figures S1, S2). The middle-SDI regions had the highest DALYs, but the highest DALY rates were observed in the high-SDI regions. Unlike males in low–middle- and low-SDI regions, males in other SDI regions presented higher DALYs and DALY rates than females did (Supplementary Tables S1 and S2).

In terms of mortality trends, all SDI regions presented an increasing trend in mortality rates, with the DALY rate trend being consistent with the mortality rate trend (Figure 1; Supplementary Table S1).

### 3.6 High-BMI-related burden by age group

In 2021, the disease burden among adolescents and young adults attributable to high BMI markedly increased with increasing age, particularly in the 35–39 years age group (Supplementary Tables S6, S7). Individuals under 25 years of age predominantly experienced mortality due to type 2 diabetes, whereas those aged 25–39 years faced not only elevated risks of type 2 diabetes but also increased susceptibility to chronic kidney injury. With increasing age, the mortality rates from stroke and hypertensive heart disease caused by high BMI also gradually increased (Supplementary Figure S2).

## 4 Discussion

### 4.1 Key findings

Based on the latest 2021 GBD data analysis, this study comprehensively summarizes the global disease burden of high BMI among young populations over the three decades from 1990 to 2021. Previous GBD studies have mostly focused on the entire population, but the rapid increase in BMI among adolescents has not been given sufficient attention. This study found that the growth rate of DALY among young people in low- and middle-income countries was 2.3 times that of the older adult, highlighting the urgency of early intervention. Furthermore the findings indicate that the burden related to high BMI has not been effectively curbed worldwide and continues to rise, particularly in middle-SDI and low–middle-SDI regions (where the number of deaths in 2021 increased by approximately one-third compared with that in 1990, and the mortality rate also significantly increased). This trend may be associated with dietary structure adjustments and improved health care coverage in these regions.

However, some high-SDI regions exhibited a reverse trend. Observational data revealed that in certain countries in Central Europe and the high-income Asia-Pacific region, the number of deaths and mortality rates in 2021 decreased by ~50% compared with those in 1990. An examination of relevant policies and dietary habits in these countries suggests that high-SDI regions place greater emphasis on balanced diets and self-health maintenance, thereby reducing the incidence of high BMI. Additionally, due to the advanced medical standards in high-SDI regions, early intervention for high-BMI populations and preventive measures for those at risk near threshold levels can be implemented promptly.

Nevertheless, many high-SDI regions still experience an increasing prevalence of high BMI, and the overall trend in the number of deaths and mortality rates is increasing. Consequently, high BMI continues to pose a significant disease burden for young populations globally.

In terms of sex, high BMI exhibited distinct trends and variations across different sex groups. In most regions globally, the mortality rate and number of deaths among young males are higher than those among females in the same regions, with the opposite trend observed only in low-SDI areas and older adult populations (32). The underlying reasons include the frequent association of male obesity with “muscularity” or “health,” leading to delayed diagnosis and intervention, whereas females are more likely to be screened and monitored due to aesthetic standards, facilitating early intervention (33). Additionally, males are more prone to fat accumulation, whereas females tend to distribute subcutaneous fat more in the hips and thighs, which has a less negative metabolic impact. This difference may be linked to hormonal levels (testosterone vs. estrogen), Estrogen can regulate adipocyte differentiation through nuclear receptors (ERα/ERβ) and membrane receptors (GPER). Additionally, estrogen can inhibit the accumulation of visceral fat (the accumulation of visceral fat after the decline of estrogen levels in men can also serve as evidence for this) and promote the development of subcutaneous fat through IGF-1R and PPARγ signaling. However, in male obese individuals, it leads to a decrease in testosterone secretion, thereby reducing the inhibitory effect of testosterone on the differentiation of preadipocytes (34). Resulting in male obesity being more strongly associated with hypertension, high triglycerides, and low HDL-C, whereas female obesity is more strongly correlated with diabetes (32, 35, 36). Consequently, males present higher mortality rates and death counts than females do. Furthermore, males in high- and middle–high-SDI regions face greater work pressure and life burdens, leading to endocrine disorders and other complications that exacerbate the severity of high BMI.

Among the 15–39 years age group, different age cohorts presented varying burdens of high-BMI-related diseases. From 1990 to 2021, both the number of deaths and mortality rates increased among adolescents and young adults, with the older subgroups demonstrating a more pronounced upwards trajectory than the younger subgroups did. Concurrently, disease risk attributable to high BMI progressively increased with age. Encouragingly, younger age groups presented lower death counts and mortality rates, suggesting that early intervention could achieve effective risk control—an outcome contingent upon the school and family supervision of adolescent populations.

The DALYs and DALY rate of young adults have also significantly increased compared to those of teenagers. However, when compared longitudinally, the ratio of disability-adjusted life years (DALYs) between the two groups has increased in both 2021 and 1990. Moreover, the growth rate and trend of young adults are more obvious. On one hand, this is significantly related to hormonal fluctuations that cause abnormal appetite regulation (such as leptin resistance) and changes in fat distribution (centripetal obesity). Studies have shown that more than 90% of obese adolescents maintain overweight or obese status in adulthood, and early obesity can exacerbate the risk of metabolic syndrome in adulthood (such as hypertension, insulin resistance) (23). On the other hand, obesity during adolescence (12–19 years old) is directly related to visceral fat accumulation and increased arterial stiffness. Young adults (over 20 years old) are more likely to experience a cluster effect of metabolic syndrome (hypertension + high blood sugar + abnormal lipid levels). The reason is that young adults face new triggers such as workplace stress and social engagements, making them more likely to have a high BMI and obesity (37). In terms of growth and development, adolescents are in a rapid growth period (especially during puberty), and the development of bones, muscles, and organs requires a large amount of energy, with a high basal metabolic rate (BMR). Even if they consume more calories, they may be used to support height and weight growth, and fat accumulation is relatively less. After adulthood, growth and development are basically completed, metabolic demands decrease, and the basal metabolic rate gradually decreases. If calorie intake is not adjusted accordingly, excess energy is more likely to be converted into fat storage.

Additionally, the BMI risk factor exhibits unconventional patterns across different regions. In China, which is classified as a middle-SDI region, Over the past four decades, the rates of overweight and obesity among all age groups (children, adolescents, adults) in China have risen significantly and rapidly. The latest (2015–19) national and population-specific obesity rates based on Chinese standards (such as 34.3% for adults being overweight and 16.4% obese) (38). And when we combine this with the growth curve of disability-adjusted life years (DALY) it was also found that China's rate increased from 132.04 to 232.37 between 1990 and 2021, ranking fifth among 204 countries and regions, which is also in line with China's research on its obese population. The reason for this is that dietary shifts have led increasing numbers of younger individuals to adopt high-salt and high-fat diets—a marked departure from nutritional patterns two decades ago. This dietary transition constitutes the primary driver behind China's rising BMI-attributable mortality. Therefore, Barry Popkin and other scholars proposed the Nutrition Transition Theory, which describes the process by which people's dietary structure changes from a traditional low-calorie, high-fiber diet to a high-calorie, high-fat, and high-sugar processed food diet as economic development and social changes occur (39). These modern diets characterized by animal-based foods, processed foods, fast food, etc., have shown a positive correlation trend with the risk of overweight/obesity when analyzed through meta-analysis (40). Longitudinal studies tend to show an increasing risk. They believe that this is closely related to the increase in disposable income, families' tendency to purchase high-calorie, convenient foods (such as fried foods, sugary beverages), changes in food prices, and the low cost of industrially produced processed foods (such as corn syrup replacing sugar), which squeeze the traditional food market. In addition, the accelerating pace of urban life, the surge in demand for fast food and takeout, but the decrease in physical activity, the change in transportation methods (cars replacing walking), and the decline in physical labor in occupations have all contributed to the occurrence of high BMI. Moreover, improvement in medical accessibility has increased the detection rate of high-risk diseases (such as diabetes and hypertension), resulting in more survivors suffering from chronic complications. These survivors accumulate disability over time, increasing YLDs (years of life lost due to disability), thereby increasing overall DALYs (disability-adjusted life years) even as mortality rates decline (41). Although the increase in DALYs reflects the success in avoiding premature deaths, it also warns that the health system has failed to prevent the disability of survivors. This requires shifting the focus from treatment to rehabilitation and quality-of-life interventions. Improved health care accessibility and regional medical coverage enable adolescents and young adults affected by high BMIs to receive better and more timely medical interventions, which paradoxically contributes to their elevated DALY rates.

In high-SDI regions such as Nauru, the gradual adoption of high-salt, high-fat diets coupled with public apathy toward obesity has resulted in high-BMI-related death rates. In contrast, Central European nations such as Switzerland—also a high-SDI region—maintain comparatively low death rates due to balanced dietary habits and strong national emphasis on BMI monitoring.

Notably, Zimbabwe, a low-SDI country, has a high-BMI-attributable number of deaths and mortality rate, likely attributable to its underdeveloped health care infrastructure relative to other regions.

Analyzing data from 204 countries and territories globally reveals a clear trend: nations in higher-SDI regions with greater governmental focus on BMI risk mitigation present lower-BMI-related death rates. Conversely, regions with advanced health care systems present higher DALYs and rate burdens among BMI-affected populations.

### 4.2 Driving factors

In light of the current global prevalence of high BMI, identifying its driving factors is essential to guide further prevention and early correction of high BMI, thereby promoting public health and alleviating medical burdens. Among various factors, dietary pattern changes have emerged as a significant “driver” of high BMI in recent years. With improvements in living standards, people—especially adolescents—find it easier and more appealing to consume high-sugar and high-fat foods, compounded by irregular eating habits, contributing to the current high BMI situation.

On the one hand, high-sugar diets disrupt gut-brain axis signaling, reduce sensitivity to satiety hormones (e.g., leptin), and increase ghrelin secretion, leading to hyperphagia. On the other hand, prolonged high sugar intake may induce insulin resistance, further exacerbating metabolic syndrome and obesity (9). Concurrently, obese individuals exhibit heightened food addiction tendencies (42), worsening obesity among high-BMI populations.

Severe physical inactivity among younger people is another major contributor to high BMI. With advancements in education and work environments, adolescents across different countries and regions have adopted sedentary study and work habits while neglecting physical exercise. Long-term cohort studies indicate that declining physical activity levels are closely associated with fat accumulation and increased waist circumference, particularly amid reduced occupational activity and the widespread adoption of sedentary lifestyles (43). Research shows that sedentary behavior (activities with energy expenditure ≤ 1.5 METs, such as sitting/lying) is significantly correlated with high BMI, independent of physical activity level. The underlying mechanism involves reduced muscle insulin sensitivity and impaired fat breakdown due to inactivity, promoting fat storage (44). Furthermore, the 2018 Scientific Report of the U.S. Physical Activity Guidelines Advisory Committee updated the importance of sedentary behavior as an independent health risk factor, although its impact on BMI should be assessed in conjunction with physical activity levels.

From the perspective of the living environment, it also has a profound impact on human behavior. In some regions, high-density traffic exacerbates PM2.5 and NO_2_ pollution, discouraging outdoor activities and reducing overall physical exertion. Developing countries commonly face issues such as narrow sidewalks and motorway encroachment on pedestrian spaces (e.g., sidewalks compressed to <1 m wide alongside multilane highways), increasing safety risks for pedestrians. Mixed-use communities (e.g., slums in India) often suffer from poor sanitation, security hazards, and noise pollution, forcing residents to limit outdoor activities (45). Additionally, urban green space availability and school environments significantly affect adolescents' willingness to engage in physical activity (46).

An analysis of youth obesity revealed that sociocultural factors also play a crucial role in high BMI. One factor is adolescents' increased acceptance of obesity, which enhances their self-concept and emotional stability (47). Another is insufficient health awareness—many young individuals remain unaware of the bodily burden and long-term consequences of high BMI, habitually overeating and consuming excessive sugary beverages, disrupting metabolic regulation and leading to high BMI. These obese adolescents also lack awareness of weight control and maintaining metabolic balance, perpetuating a vicious cycle that worsens their condition.

### 4.3 Public health recommendations

To address the current high BMI prevalence among adolescents, coordinated efforts across policies, schools, and multiple sectors are needed to reduce its incidence. At the policy level, implementing a sugar-sweetened beverage tax has proven effective, with studies showing sustained declines in purchases within 2 years of adoption in certain countries. Concurrently, robust food labeling policies—such as prominent warnings on high-calorie products—can curb consumer demand, as evidenced by Chile's successful obesity rate reduction (48). However, policy implementation faces challenges, including industry resistance, weak enforcement mechanisms, and insufficient supporting measures. A tripartite strategy combining “fiscal regulation + social mobilization + research support” is essential for systemic obesity prevention (49).

As the primary setting for adolescent activities, schools play a pivotal role. Health education programs should prioritize weight management awareness, with trained teachers serving as behavioral models (e.g., Greece's “Child Health Study,” which involves educators in curriculum design) (50). Enhanced physical activity initiatives further mitigate high BMI risks: PE curricula should emphasize skill-building and enjoyment, whereas non-PE hours should maximize movement opportunities through structured games or interactive infrastructure (e.g., marked play zones) (51). School-family collaboration is equally critical—homework, parent-teacher meetings, and distributed materials (e.g., Germany's “Healthy Habits Program” with cooking classes or Spain's nutrition guides) can promote dietary improvements (e.g., increased produce intake) and screen-time limits (modeled by the U.S. “Transform Project”) (50). Take China as an example. Although traditional dietary culture involves high carbohydrate intake (such as noodles and rice), in recent years, through the control of school-based meals for teenagers, the adjustment of the dietary concepts of young adults, and the collaboration of digital health management platforms, this success is attributed to two innovative points: (1) Integrating TCM constitution identification into dietary guidance; (2) Establishing a three-level digital monitoring network for home, school, and society. In contrast, India, when promoting similar projects, neglected local dietary preferences (such as the preference for ghee tea in northern India), resulting in a decline in intervention effectiveness. Such multifaceted interventions foster early BMI management.

Cross-sector coordination is equally vital. Community-primary health care partnerships can deliver home visits, dietary guidance, and exercise monitoring, particularly in underserved areas. Private sector engagement, such as tech-developed remote obesity tracking tools, complements public policies (52). However, barriers persist: rural resource constraints hinder sustained collaboration, whereas inadequate evaluation frameworks obscure sector-specific contributions. Solutions include the use of multiple ministerial policies (e.g., education-health-agriculture alliances) for resource optimization and mechanistic studies to quantify the long-term BMI impacts of collaborative models (53), thereby streamlining intersectoral efficacy. It is worth noting that the enhancement of self-health management awareness is emerging as a significant new force in combating childhood obesity. With the widespread adoption of wearable devices (such as smartwatches) and mobile health applications, individuals can achieve dynamic health management by monitoring real-time indicators such as BMI, activity levels, and sleep quality. This technology-enabled self-management approach is highly scalable and particularly suitable for promotion in urban environments. However, its effectiveness is highly dependent on cultural adaptability–for instance, in China, integrating traditional health preservation concepts with modern monitoring technologies can significantly enhance the compliance of teenagers.

For different countries and regions, corresponding policies should be formulated based on local conditions to better align with national development trends and the lifestyles of the people. For high SDI areas, personalized dietary guidance and monitoring programs can be promoted. For example, countries like Finland and Portugal have established national nutrition monitoring systems as priority infrastructure projects, including regular dietary surveys and health outcome tracking (such as obesity rates) (54). For medium SDI areas, the intervention of obesity and diabetes should be included in the core services of UHC (Universal Health Coverage), such as diabetes treatment and cardiovascular disease management, especially for the adolescent population. Use the effective coverage index of UHC (such as mortality-incidence ratio, treatment coverage) to monitor the health service gap for adolescents and guide resource allocation toward the prevention and control of these diseases (55). For low SDI areas, the top priority is to improve infrastructure construction, provide a good environment for exercise, stimulate teenagers to increase physical exercise to cultivate a healthier lifestyle, and reduce the burden of diseases with high BMI.

### 4.4 Limitations

Although this study analyzed the high-BMI population in the database from 1990 to 2021 and identified their trends and correlations, several limitations remain. On the one hand, the GBD database mentions only the high-BMI population without further classification. For example, obesity is not differentiated into central obesity [defined as excessive abdominal fat accumulation, with diagnostic criteria of waist circumference ≥90 cm for men and ≥80 cm for women in South Asian populations (56)] and peripheral obesity. With the same BMI, the degree of harm caused by central obesity is less than that caused by generalized obesity, so health risks cannot be solely determined based on the BMI.

On the other hand, BMI does not fully reflect an individual's obesity status and should be evaluated comprehensively alongside indicators such as body fat percentage (BF%), waist circumference (WC), and visceral adipose tissue (VAT) (56). Many individuals may not have a high BMI but exhibit high visceral fat and body fat percentages, which still significantly increases their risk of cardiovascular diseases and other metabolic disorders. Therefore, their health conditions should be closely monitored, and early intervention strategies should be implemented to delay disease onset and reduce medical burdens.

Moreover, the GBD database relies on reports from various countries, which may introduce measurement bias. Different countries may have varying standards for high BMI or employ different BMI calculation methods, all of which could affect the final statistical results and the reliability of the research findings.

Finally, the data do not specify whether high BMI is primary or secondary. At the same time, some secondary factors also contribute to the occurrence of high BMI such as polycystic ovary syndrome (PCOS), hypothyroidism, or genetic predispositions (e.g., gene variants such as FTO and MC4R, which are significantly associated with obesity risk) (57, 58). The control and intervention methods for primary high BMI may not fully apply to these secondary cases. However, the GBD database cannot establish direct links between these factors and high BMI, necessitating further research to elucidate their correlations.

## 5 Conclusion

The high BMI disease burden among global adolescents and young adults shows significant regional and social differentiation. The increase is most pronounced in the medium-low SDI regions, while in the high SDI regions, it shows a polarized trend. The risk for men is significantly higher than that for women, but in the low SDI regions, the mortality rate for women exceeds that for men. The age gradient effect is prominent, with the DALY rate for the 35–39 age group being significantly higher than that of the adolescent group. In response to these situations, differentiated intervention strategies are recommended: in middle- and low-income countries, pilot programs involving sugar-sweetened beverage taxes (significantly reducing the obesity rate among Mexican children), promoting the Finnish “Healthy School” model (further reducing the BMI overexposure rate), and establishing a multi-sectoral collaboration network (the “5-2-1-0” program in the United States has led to a decrease in BMI). In the future, attention should be focused on the interaction between central obesity and BMI, digital health intervention technologies, and the molecular mechanisms of intergenerational transmission, in order to further control the incidence of high BMI among adolescents and young adults worldwide.

## Data Availability

The datasets presented in this study can be found in online repositories. The names of the repository/repositories and accession number(s) can be found in the article/Supplementary material.
