# Supplementary material for: Global, regional, and national disease burden attributable to high body mass index in youth and young adults: 2021 global burden of disease study analysis
Source: Front Public Health. 2025 Nov 17;13:1680402. doi: 10.3389/fpubh.2025.1680402 (PMC12665546; doi:10.3389/fpubh.2025.1680402)
Supplement: Supplementary file 1 [file Data_Sheet_1.docx]

**ble S1.** Changes in High BMI -related deaths and death rates in the youth and young adults by sex and SDI region- 1990-2021

| **Location** | **Death cases (95% UI)** | | **Percentage change in death case (95%UI)** | **Death rate/100- 000(95%UI)** | | **Percentage change in death rate/100- 000 (95%UI)** | **EAPC of death rate**  **/100- 000 (95%CI)** |
| --- | --- | --- | --- | --- | --- | --- | --- |
|  | **1990** | **2021** | **1990-2021** | **1990** | **2021** | **1990-2021** | **1990-2021** |
| **Global** | | | | | | | |
| **Global both** | 45579.84(24580.60-73819.16) | 95102.93(48227.30-148807.26) | 108.65(87.84-129.23) | 2.08(1.12-3.37) | 3.20(1.62-5.00) | 53.73(38.40-68.90) | 1.27(1.21-1.33) |
| Global woman | 20177.44(11253.02-33128.63) | 37995.11(20216.49-57229.32) | 88.30(65.67-115.85) | 1.86(1.04-3.06) | 2.59(1.38-3.91) | 39.24(22.50-59.60) | 0.93(0.85-1.02) |
| Global man | 25402.40(13157.06-40948.33) | 57107.82(28544.33-91742.48) | 124.81(102.70-146.21) | 2.29(1.19-3.69) | 3.78(1.89-6.08) | 65.06(48.83-80.78) | 1.52(1.46-1.57) |
| **SDI region** | | | | | | | |
| **High SDI both** | 6758.07(3522.45-10230.39) | 9109.90(5424.26-12819.17) | 34.80(17.77-58.82) | 1.95(1.02-2.95) | 2.58(1.54-3.63) | 32.41(15.68-56.00) | 0.96(0.79-1.12) |
| High SDI woman | 2070.74(1134.30-3041.18) | 2923.91(1751.69-4035.80) | 41.20(23.14-64.23) | 1.22(0.67-1.79) | 1.71(1.03-2.37) | 40.93(22.91-63.92) | 1.05(0.89-1.22) |
| High SDI man | 4687.33(2376.63-7224.12) | 6185.99(3701.54-8790.74) | 31.97(14.80-58.78) | 2.65(1.34-4.09) | 3.39(2.03-4.81) | 27.67(11.06-53.61) | 0.88(0.71-1.06) |
| **High-middle SDI both** | 8964.14(4661.09-14081.90) | 11770.34(5404.16-18597.26) | 31.30(15.02-46.74) | 1.98(1.03-3.11) | 2.67(1.23-4.22) | 34.96(18.23-50.83) | 0.43(0.26-0.61) |
| High-middle SDI woman | 2955.34(1609.00-4610.78) | 3293.40(1646.81-4939.49) | 11.44(-5.44-29.54) | 1.33(0.72-2.07) | 1.56(0.78-2.34) | 17.30(-0.47-36.35) | 0.16(0.02-0.31) |
| High-middle SDI man | 6008.79(3063.01-9570.30) | 8476.94(3911.32-13526.44) | 41.08(22.17-60.97) | 2.61(1.33-4.16) | 3.70(1.71-5.91) | 41.79(22.79-61.78) | 0.52(0.32-0.72) |
| **Middle SDI both** | 16253.28(8877.95-25942.59) | 33460.26(16799.23-51475.08) | 105.87(80.08-127.64) | 2.16(1.18-3.45) | 3.61(1.81-5.55) | 67.06(46.13-84.72) | 1.53(1.42-1.65) |
| Middle SDI woman | 7446.32(4120.67-12013.38) | 12422.68(6409.65-18645.36) | 66.83(44.34-93.38) | 2.01(1.11-3.24) | 2.73(1.41-4.09) | 35.85(17.53-57.47) | 0.77(0.59-0.96) |
| Middle SDI man | 8806.97(4799.48-14034.83) | 21037.58(10438.32-32754.30) | 138.87(104.14-166.84) | 2.31(1.26-3.68) | 4.46(2.21-6.94) | 93.19(65.09-115.81) | 2.07(1.99-2.15) |
| **Low-middle SDI both** | 9955.03(5241.47-17014.72) | 28569.87(14330.41-45817.91) | 186.99(148.30-234.38) | 2.20(1.16-3.75) | 3.56(1.79-5.71) | 62.15(40.29-88.92) | 1.65(1.59-1.72) |
| Low-middle SDI woman | 5517.50(2912.42-9714.39) | 13133.16(6937.28-20468.55) | 138.03(97.88-191.62) | 2.45(1.29-4.31) | 3.29(1.74-5.13) | 34.33(11.68-64.58) | 0.97(0.91-1.03) |
| Low-middle SDI man | 4437.53(2325.22-7578.88) | 15436.70(7500.02-24977.60) | 247.87(193.74-305.36) | 1.95(1.02-3.32) | 3.83(1.86-6.19) | 96.76(66.14-129.27) | 2.36(2.27-2.45) |
| **Low SDI both** | 3578.50(2123.08-6264.65) | 12088.71(6609.95-19691.47) | 237.81(182.01-304.37) | 1.94(1.15-3.40) | 2.69(1.47-4.39) | 38.65(15.74-65.97) | 1.00(0.94-1.06) |
| Low SDI woman | 2159.42(1243.33-3818.74) | 6174.82(3551.03-9634.56) | 185.95(133.32-258.01) | 2.31(1.33-4.09) | 2.72(1.56-4.24) | 17.52(-4.11-47.14) | 0.43(0.36-0.49) |
| Low SDI man | 1419.08(805.58-2537.66) | 5913.89(3037.81-10165.54) | 316.74(245.09-406.84) | 1.56(0.89-2.79) | 2.67(1.37-4.59) | 70.80(41.44-107.73) | 1.72(1.62-1.83) |
| High BMI- high body mass index; SDI- sociodemographic index; UI- uncertainty interval; CI-confidence interval; EAPC- estimated annual percentage change. | | | | | | | |

**Table S2.** Changes in High BMI-related DALYs and DALY rates in the youth and young adults by sex and SDI region- 1990-2021

| **Location** | **DALYs (95% UI)** | | **Percentage change in DALYs (95%UI)** | **DALY rate/100- 000 (95%UI)** | | **Percentage change in DALY rate/100- 000 (95%UI)** | **EAPC of DALY rate/100- 000 (95%CI)** |
| --- | --- | --- | --- | --- | --- | --- | --- |
|  | **1990** | **2021** | **1990-2021** | **1990** | **2021** | **1990-2021** | **1990-2021** |
| **Global** | | | | | | | |
| **Global Both** | 4733088.15(2084809.24-7910759.74) | 11428432.13(5195837.94-18184470.87) | 141.46(119.87-163.35) | 215.95(95.12-360.93) | 384.17(174.66-611.28) | 77.90(62.00-94.03) | 1.83(1.80-1.85) |
| Global woman | 2367584.06(1016630.37-4027342.21) | 5439410.01(2405129.69-8818615.37) | 129.75(107.80-154.51) | 218.55(93.84-371.75) | 371.26(164.16-601.90) | 69.88(53.65-88.19) | 1.67(1.64-1.71) |
| Global man | 2365504.09(1069425.10-3893723.35) | 5989022.12(2838287.88-9383517.61) | 153.18(129.52-176.83) | 213.40(96.48-351.27) | 396.70(188.00-621.55) | 85.89(68.52-103.26) | 1.97(1.94-2.00) |
| **SDI region** | | | | | | | |
| **High SDI both** | 1044695.94(394887.42-1773710.69) | 1753422.03(767238.56-2789234.06) | 67.84(51.45-92.49) | 301.10(113.81-511.21) | 496.38(217.20-789.61) | 64.86(48.76-89.07) | 1.64(1.55-1.72) |
| High SDI woman | 475786.90(162975.01-842440.66) | 812606.65(321494.75-1343167.46) | 70.79(54.28-96.18) | 279.52(95.75-494.92) | 476.49(188.52-787.60) | 70.47(53.99-95.81) | 1.74(1.67-1.81) |
| High SDI man | 568909.03(231912.41-926872.37) | 940815.37(445743.82-1462285.69) | 65.37(48.69-90.59) | 321.88(131.21-524.40) | 514.95(243.97-800.37) | 59.98(43.84-84.38) | 1.54(1.45-1.64) |
| **High-middle SDI both** | 995060.03(408923.58-1627943.14) | 1768661.46(755729.87-2814994.44) | 77.74(61.27-94.50) | 219.88(90.36-359.74) | 401.72(171.65-639.38) | 82.70(65.77-99.92) | 1.71(1.61-1.81) |
| High-middle SDI woman | 435188.82(170913.54-735275.07) | 747149.37(299645.67-1218974.84) | 71.68(53.10-90.27) | 195.60(76.82-330.47) | 353.46(141.76-576.67) | 80.71(61.15-100.27) | 1.80(1.71-1.89) |
| High-middle SDI man | 559871.21(247491.79-904027.74) | 1021512.09(456210.38-1606937.59) | 82.45(65.22-102.68) | 243.37(107.58-392.98) | 446.30(199.32-702.07) | 83.38(66.06-103.70) | 1.63(1.52-1.75) |
| **Middle SDI both** | 1549630.40(727937.80-2521715.19) | 3834480.61(1788927.11-6015002.08) | 147.44(120.98-169.20) | 205.89(96.72-335.05) | 413.43(192.88-648.52) | 100.79(79.32-118.45) | 2.18(2.10-2.27) |
| Middle SDI woman | 782668.91(358435.64-1302523.93) | 1760963.35(785834.40-2795037.07) | 124.99(95.91-149.54) | 211.01(96.64-351.17) | 386.60(172.52-613.61) | 83.21(59.53-103.20) | 1.84(1.73-1.95) |
| Middle SDI man | 766961.49(377230.76-1218134.90) | 2073517.26(989596.18-3209231.99) | 170.35(141.11-195.42) | 200.92(98.82-319.12) | 439.32(209.67-679.94) | 118.65(95.00-138.92) | 2.51(2.43-2.58) |
| **Low-middle SDI both** | 842782.20(399227.70-1432594.55) | 2886912.26(1342653.85-4579034.76) | 242.55(199.04-289.51) | 185.88(88.05-315.97) | 359.74(167.31-570.60) | 93.53(68.96-120.07) | 2.23(2.19-2.27) |
| Low-middle SDI woman | 488876.59(226221.98-852590.10) | 1476432.50(670274.31-2387439.53) | 202.01(158.96-249.89) | 217.02(100.42-378.48) | 369.89(167.92-598.13) | 70.44(46.15-97.46) | 1.77(1.74-1.80) |
| Low-middle SDI man | 353905.61(172086.94-611507.27) | 1410479.76(667345.23-2242249.29) | 298.55(239.04-356.61) | 155.13(75.43-268.05) | 349.70(165.45-555.92) | 125.42(91.76-158.26) | 2.78(2.72-2.84) |
| **Low SDI both** | 294037.74(156839.89-513279.76) | 1173284.11(573379.89-1911808.28) | 299.03(238.70-356.55) | 159.54(85.10-278.49) | 261.27(127.68-425.73) | 63.77(39.01-87.38) | 1.57(1.54-1.59) |
| Low SDI woman | 181823.59(95438.99-310949.46) | 636221.68(314737.88-1026467.07) | 249.91(194.79-313.14) | 194.53(102.11-332.68) | 279.75(138.39-451.35) | 43.81(21.16-69.80) | 1.12(1.08-1.16) |
| Low SDI man | 112214.15(58736.70-193925.98) | 537062.43(256994.73-887957.64) | 378.60(302.90-459.84) | 123.53(64.66-213.48) | 242.31(115.95-400.63) | 96.16(65.13-129.46) | 2.17(2.10-2.24) |
| DALYs- disability-adjusted life years; DALY- disability-adjusted life year; SDI- sociodemographic index; UI- uncertainty interval; CI-confidence interval; EAPC- estimated annual percentage change. | | | | | | | |

**Table S3.** Ranks of High BMI-related deaths- death rates- and changes in the youth and young adults in 204 countries/territories- 1990-2021

| **The rank of death cases** | | |  | **The rank of percentage change in death case** | | |  | **The rank of death rates** | | |  | **The rank of percentage change in death rate** | | |
| --- | --- | --- | --- | --- | --- | --- | --- | --- | --- | --- | --- | --- | --- | --- |
| **(Descending)** | | |  | **(Descending) 1990-2021** | | |  | **(Descending)** | | |  | **(Descending) 1990-2021** | | |
| **Country or region** | **Rank** | |  | **Country or region** | **Rank** | **Value (95%UI)** |  | **Country or region** | **Rank** | |  | **Country or region** | **Rank** | **Value (95%UI)** |
|  | **1990** | **2021** |  |  |  |  |  |  | **1990** | **2021** |  |  |  |  |
| China | 1 | 2 |  | Zimbabwe | 1 | 648.80(349.37-1014.32) |  | Nauru | 1 | 1 |  | Zimbabwe | 1 | 368.43(181.11-597.09) |
| India | 2 | 1 |  | Saudi Arabia | 2 | 547.70(326.97-914.29) |  | Kiribati | 2 | 3 |  | Lesotho | 2 | 277.56(133.08-473.30) |
| United States of America | 3 | 6 |  | Pakistan | 3 | 499.02(338.98-732.04) |  | Marshall Islands | 3 | 2 |  | Thailand | 3 | 198.47(111.09-327.21) |
| Brazil | 4 | 8 |  | Lesotho | 4 | 482.06(259.32-783.83) |  | Tuvalu | 4 | 6 |  | Libya | 4 | 164.21(87.90-256.99) |
| Egypt | 5 | 4 |  | Equatorial Guinea | 5 | 462.66(217.45-924.94) |  | Micronesia | 5 | 5 |  | Eswatini | 5 | 163.31(57.57-306.74) |
| Russian Federation | 6 | 13 |  | Djibouti | 6 | 459.74(227.57-844.08) |  | Cook Islands | 6 | 15 |  | Pakistan | 6 | 147.13(81.11-243.27) |
| Indonesia | 7 | 5 |  | Cameroon | 7 | 446.75(275.65-668.25) |  | Palau | 7 | 4 |  | Saudi Arabia | 7 | 132.38(53.18-263.90) |
| Mexico | 8 | 7 |  | Qatar | 8 | 436.31(304.96-654.93) |  | Fiji | 8 | 7 |  | Viet Nam | 8 | 122.86(33.29-266.68) |
| South Africa | 9 | 12 |  | Mozambique | 9 | 431.73(231.12-727.98) |  | Niue | 9 | 8 |  | China | 9 | 117.71(45.26-183.71) |
| Philippines | 10 | 9 |  | Kenya | 10 | 425.17(279.43-614.86) |  | Tokelau | 10 | 9 |  | Panama | 10 | 117.46(60.50-187.45) |
| Turkey | 11 | 20 |  | Uganda | 11 | 403.08(212.47-693.49) |  | American Samoa | 11 | 10 |  | Kenya | 11 | 112.42(53.47-189.15) |
| Pakistan | 12 | 3 |  | Gambia | 12 | 400.16(236.92-635.86) |  | Northern Mariana Islands | 12 | 20 |  | Ukraine | 12 | 110.30(38.59-200.47) |
| Myanmar | 13 | 21 |  | United Arab Emirates | 13 | 397.81(252.95-566.45) |  | Egypt | 13 | 16 |  | Mozambique | 13 | 109.76(30.62-226.62) |
| Germany | 14 | 60 |  | Libya | 14 | 371.84(235.56-537.53) |  | Samoa | 14 | 11 |  | Dominica | 14 | 105.84(53.15-169.24) |
| Nigeria | 15 | 11 |  | Yemen | 15 | 366.28(199.18-635.77) |  | South Africa | 15 | 28 |  | Bangladesh | 15 | 103.16(30.99-214.78) |
| Poland | 16 | 67 |  | Sierra Leone | 16 | 362.49(209.84-614.91) |  | Vanuatu | 16 | 12 |  | Sierra Leone | 16 | 98.08(32.70-206.19) |
| Ethiopia | 17 | 26 |  | Afghanistan | 17 | 362.24(229.81-609.76) |  | Syrian Arab Republic | 17 | 30 |  | Indonesia | 17 | 97.71(45.81-174.25) |
| Iran | 18 | 15 |  | Eswatini | 18 | 344.84(166.21-587.15) |  | Saint Kitts and Nevis | 18 | 95 |  | Solomon Islands | 18 | 96.05(26.05-262.90) |
| Bangladesh | 19 | 14 |  | Angola | 19 | 342.37(190.11-590.11) |  | Trinidad and Tobago | 19 | 17 |  | Dominican Republic | 19 | 90.70(47.22-147.23) |
| Morocco | 20 | 27 |  | Congo | 20 | 341.24(177.78-619.23) |  | Solomon Islands | 20 | 13 |  | Mauritius | 20 | 89.91(57.32-131.07) |
| Sudan | 21 | 17 |  | Chad | 21 | 338.18(183.37-590.24) |  | United States Virgin Islands | 21 | 25 |  | Congo | 21 | 88.66(18.77-207.52) |
| Ukraine | 22 | 30 |  | Benin | 22 | 330.48(192.66-536.00) |  | Guyana | 22 | 19 |  | Gambia | 22 | 88.55(27.01-177.41) |
| Iraq | 23 | 24 |  | Côte d'Ivoire | 23 | 326.56(191.27-528.32) |  | Bahamas | 23 | 24 |  | Uganda | 23 | 87.63(16.54-195.94) |
| Argentina | 24 | 44 |  | Togo | 24 | 324.60(176.94-543.13) |  | Tonga | 24 | 18 |  | Central African Republic | 24 | 85.33(16.36-193.28) |
| Thailand | 25 | 19 |  | Solomon Islands | 25 | 318.06(168.79-673.86) |  | Afghanistan | 25 | 29 |  | Tunisia | 25 | 83.93(31.91-162.12) |
| Saudi Arabia | 26 | 10 |  | Democratic Republic of the Congo | 26 | 317.82(178.69-535.10) |  | Guam | 26 | 21 |  | Democratic People's Republic of Korea | 26 | 83.44(12.72-207.51) |
| Syrian Arab Republic | 27 | 43 |  | Liberia | 27 | 313.14(178.61-512.61) |  | Grenada | 27 | 44 |  | Jamaica | 27 | 81.97(34.16-158.05) |
| Colombia | 28 | 38 |  | United Republic of Tanzania | 28 | 305.60(168.51-474.41) |  | Hungary | 28 | 139 |  | Djibouti | 28 | 81.48(6.20-206.09) |
| Algeria | 29 | 25 |  | Central African Republic | 29 | 288.55(143.97-514.88) |  | Papua New Guinea | 29 | 27 |  | Botswana | 29 | 80.72(7.70-209.63) |
| Venezuela | 30 | 32 |  | Eritrea | 30 | 283.13(109.78-574.13) |  | Iraq | 30 | 71 |  | Côte d'Ivoire | 30 | 79.96(22.88-165.08) |
| United Kingdom | 31 | 57 |  | Oman | 31 | 282.19(166.93-449.18) |  | Saudi Arabia | 31 | 14 |  | Eritrea | 31 | 77.32(-2.91-212.01) |
| Japan | 32 | 75 |  | Guinea | 32 | 281.46(136.59-514.04) |  | Puerto Rico | 32 | 59 |  | Sao Tome and Principe | 32 | 76.06(1.35-225.16) |
| Democratic Republic of the Congo | 33 | 16 |  | Belize | 33 | 280.77(212.66-385.18) |  | Seychelles | 33 | 32 |  | Palau | 33 | 76.01(28.86-150.30) |
| Republic of Korea | 34 | 96 |  | Madagascar | 34 | 277.53(138.82-501.65) |  | Georgia | 34 | 78 |  | Vanuatu | 34 | 74.05(14.11-164.93) |
| Romania | 35 | 70 |  | Botswana | 35 | 274.03(122.90-540.82) |  | Guatemala | 35 | 43 |  | Togo | 35 | 73.00(12.84-162.04) |
| Peru | 36 | 36 |  | Zambia | 36 | 272.77(140.06-467.55) |  | Bulgaria | 36 | 40 |  | Iran (Islamic Republic of) | 36 | 72.80(44.78-111.37) |
| Uzbekistan | 37 | 28 |  | Sao Tome and Principe | 37 | 272.75(114.57-588.44) |  | Qatar | 37 | 86 |  | Liberia | 37 | 69.77(14.49-151.73) |
| Hungary | 38 | 110 |  | Vanuatu | 38 | 270.32(142.78-463.68) |  | Mauritius | 38 | 23 |  | United Republic of Tanzania | 38 | 68.42(11.49-138.52) |
| Spain | 39 | 95 |  | Nigeria | 39 | 257.25(130.16-442.85) |  | Bahrain | 39 | 50 |  | Mexico | 39 | 67.99(44.89-96.39) |
| Kazakhstan | 40 | 72 |  | Papua New Guinea | 40 | 256.20(146.50-478.90) |  | Kuwait | 40 | 55 |  | Uzbekistan | 40 | 66.99(34.62-108.02) |
| Afghanistan | 41 | 18 |  | Panama | 41 | 254.80(161.87-369.00) |  | Saint Lucia | 41 | 53 |  | Suriname | 41 | 66.30(26.54-119.73) |
| United Republic of Tanzania | 42 | 22 |  | Ghana | 42 | 250.66(145.66-415.70) |  | Greenland | 42 | 135 |  | Democratic Republic of the Congo | 42 | 65.86(10.63-152.12) |
| Malaysia | 43 | 33 |  | Burkina Faso | 43 | 244.70(133.39-405.82) |  | Haiti | 43 | 47 |  | Samoa | 43 | 64.28(4.47-141.67) |
| Italy | 44 | 102 |  | Mali | 44 | 233.25(131.98-406.85) |  | Equatorial Guinea | 44 | 56 |  | Philippines | 44 | 63.36(30.66-101.10) |
| Ghana | 45 | 29 |  | Malawi | 45 | 232.30(136.00-359.53) |  | Gabon | 45 | 37 |  | India | 45 | 62.94(25.97-112.48) |
| France | 46 | 85 |  | Bangladesh | 46 | 231.21(113.55-413.18) |  | Myanmar | 46 | 87 |  | Cameroon | 46 | 61.29(10.81-126.63) |
| Sri Lanka | 47 | 58 |  | Guinea-Bissau | 47 | 230.71(121.20-429.39) |  | Brunei Darussalam | 47 | 58 |  | Namibia | 47 | 60.11(-0.37-156.64) |
| Ecuador | 48 | 51 |  | Maldives | 48 | 230.12(138.39-378.47) |  | Saint Vincent and the Grenadines | 48 | 38 |  | Tonga | 48 | 58.95(12.93-136.54) |
| Canada | 49 | 69 |  | Somalia | 49 | 230.01(103.80-399.13) |  | Barbados | 49 | 72 |  | Turkmenistan | 49 | 58.73(17.69-108.89) |
| Viet Nam | 50 | 37 |  | Bahrain | 50 | 229.11(169.11-320.24) |  | Lebanon | 50 | 114 |  | Costa Rica | 50 | 57.36(34.21-86.91) |
| Bulgaria | 51 | 88 |  | Jordan | 51 | 206.13(137.91-299.48) |  | Morocco | 51 | 67 |  | Timor-Leste | 51 | 56.99(-8.62-183.90) |
| Guatemala | 52 | 40 |  | Lao People's Democratic Republic | 52 | 204.24(92.55-379.00) |  | Mexico | 52 | 31 |  | Yemen | 52 | 55.78(-0.05-145.81) |
| Cameroon | 53 | 23 |  | Haiti | 53 | 200.66(107.02-345.77) |  | Bolivia | 53 | 90 |  | Venezuela (Bolivarian Republic of) | 53 | 55.67(17.01-106.90) |
| Cuba | 54 | 103 |  | Viet Nam | 54 | 200.01(79.44-393.62) |  | Ecuador | 54 | 79 |  | Mongolia | 54 | 55.21(19.81-107.63) |
| Kenya | 55 | 31 |  | Namibia | 55 | 199.23(86.20-379.62) |  | El Salvador | 55 | 46 |  | Marshall Islands | 55 | 54.73(16.72-112.68) |
| Azerbaijan | 56 | 83 |  | Philippines | 56 | 197.80(138.19-266.59) |  | Venezuela | 56 | 42 |  | Belarus | 56 | 54.19(17.31-104.87) |
| Chile | 57 | 79 |  | Sudan | 57 | 197.56(81.67-371.85) |  | Antigua and Barbuda | 57 | 116 |  | Trinidad and Tobago | 57 | 53.69(14.18-106.97) |
| Taiwan | 58 | 78 |  | Kuwait | 58 | 191.99(136.90-269.95) |  | Guinea-Bissau | 58 | 52 |  | El Salvador | 58 | 53.14(14.87-114.23) |
| Côte d'Ivoire | 59 | 35 |  | Nicaragua | 59 | 191.65(127.77-289.01) |  | Liberia | 59 | 36 |  | Guam | 59 | 52.13(29.57-78.21) |
| Nepal | 60 | 50 |  | India | 60 | 191.19(125.12-279.73) |  | Jordan | 60 | 104 |  | Malawi | 60 | 51.73(7.76-109.82) |
| Haiti | 61 | 47 |  | Palestine | 61 | 190.54(111.99-302.18) |  | Estonia | 61 | 165 |  | Guinea | 61 | 51.67(-5.93-144.14) |
| Dominican Republic | 62 | 49 |  | Indonesia | 62 | 188.48(112.75-300.15) |  | United Arab Emirates | 62 | 64 |  | Nicaragua | 62 | 51.26(18.12-101.74) |
| Georgia | 63 | 122 |  | Guatemala | 63 | 187.55(119.59-304.67) |  | Belize | 63 | 54 |  | Taiwan (Province of China) | 63 | 49.73(21.26-82.94) |
| Yemen | 64 | 34 |  | Dominican Republic | 64 | 182.29(117.93-265.96) |  | Brazil | 64 | 89 |  | South Sudan | 64 | 49.03(-4.36-137.31) |
| Madagascar | 65 | 42 |  | Gabon | 65 | 181.83(73.50-354.63) |  | Palestine | 65 | 84 |  | Comoros | 65 | 48.38(-5.57-213.61) |
| Bolivia | 66 | 65 |  | Timor-Leste | 66 | 181.79(64.03-409.60) |  | Poland | 66 | 171 |  | Guyana | 66 | 48.17(9.41-101.77) |
| Czechia | 67 | 130 |  | Iran (Islamic Republic of) | 67 | 176.11(131.34-237.75) |  | Turkey | 67 | 130 |  | Tokelau | 67 | 47.72(5.68-122.92) |
| Rwanda | 68 | 90 |  | Nepal | 68 | 168.23(68.60-331.33) |  | Suriname | 68 | 45 |  | Belize | 68 | 47.57(21.17-88.03) |
| Papua New Guinea | 69 | 46 |  | Uzbekistan | 69 | 167.26(115.44-232.92) |  | Azerbaijan | 69 | 115 |  | Saint Vincent and the Grenadines | 69 | 46.92(20.64-87.30) |
| Australia | 70 | 100 |  | Niger | 70 | 166.99(77.54-318.24) |  | Cameroon | 70 | 48 |  | Lao People's Democratic Republic | 70 | 46.53(-7.26-130.70) |
| Belarus | 71 | 97 |  | Comoros | 71 | 166.09(69.34-462.41) |  | Mauritania | 71 | 105 |  | Chad | 71 | 46.16(-5.48-130.24) |
| Serbia | 72 | 118 |  | Malaysia | 72 | 161.29(116.10-220.86) |  | Dominican Republic | 72 | 33 |  | Nepal | 72 | 46.15(-8.14-135.01) |
| Somalia | 73 | 52 |  | Paraguay | 73 | 160.33(91.79-247.06) |  | Philippines | 73 | 51 |  | Niue | 73 | 46.09(0.68-127.06) |
| Zambia | 74 | 48 |  | Senegal | 74 | 159.82(79.75-297.35) |  | Libya | 74 | 22 |  | Madagascar | 74 | 46.03(-7.63-132.71) |
| Mali | 75 | 53 |  | Cambodia | 75 | 159.01(65.20-334.99) |  | Tajikistan | 75 | 112 |  | Guinea-Bissau | 75 | 45.34(-2.78-132.66) |
| El Salvador | 76 | 71 |  | Burundi | 76 | 158.04(65.17-303.40) |  | Rwanda | 76 | 150 |  | American Samoa | 76 | 44.99(1.76-100.20) |
| Senegal | 77 | 59 |  | Thailand | 77 | 144.00(72.57-249.25) |  | Turkmenistan | 77 | 57 |  | Gabon | 77 | 44.62(-10.97-133.29) |
| Democratic People's Republic of Korea | 78 | 63 |  | Mexico | 78 | 142.65(109.29-183.67) |  | Central African Republic | 78 | 41 |  | Republic of Moldova | 78 | 42.85(18.86-78.44) |
| Malawi | 79 | 55 |  | Cabo Verde | 79 | 139.72(67.59-258.18) |  | Congo | 79 | 39 |  | Angola | 79 | 42.27(-6.70-121.94) |
| Uganda | 80 | 41 |  | Costa Rica | 80 | 133.14(98.84-176.92) |  | Russian Federation | 80 | 85 |  | Ghana | 80 | 40.74(-1.40-106.98) |
| Mozambique | 81 | 39 |  | Algeria | 81 | 133.08(69.72-224.78) |  | Romania | 81 | 118 |  | Fiji | 81 | 40.37(4.72-93.76) |
| Angola | 82 | 45 |  | Tunisia | 82 | 132.69(66.88-231.61) |  | Oman | 82 | 68 |  | Benin | 82 | 39.82(-4.94-106.57) |
| Puerto Rico | 83 | 119 |  | South Sudan | 83 | 132.35(49.12-269.99) |  | Algeria | 83 | 69 |  | Zambia | 83 | 39.76(-9.99-112.79) |
| Tunisia | 84 | 66 |  | Egypt | 84 | 129.79(81.06-191.73) |  | Latvia | 84 | 132 |  | Malaysia | 84 | 39.49(15.37-71.29) |
| Tajikistan | 85 | 80 |  | Kiribati | 85 | 126.51(62.14-219.22) |  | Ghana | 85 | 70 |  | Kiribati | 85 | 39.08(-0.44-96.01) |
| Slovakia | 86 | 135 |  | Honduras | 86 | 124.96(27.18-246.07) |  | Montenegro | 86 | 131 |  | Algeria | 86 | 38.32(0.72-92.74) |
| Portugal | 87 | 137 |  | Mongolia | 87 | 121.68(71.12-196.55) |  | Slovakia | 87 | 162 |  | Papua New Guinea | 87 | 37.77(-4.66-123.90) |
| Jordan | 88 | 61 |  | Democratic People's Republic of Korea | 88 | 121.21(35.93-270.83) |  | Somalia | 88 | 100 |  | Cambodia | 88 | 37.69(-12.18-131.23) |
| Libya | 89 | 54 |  | Jamaica | 89 | 120.95(62.89-213.32) |  | Eswatini | 89 | 26 |  | Oman | 89 | 37.02(-4.30-96.89) |
| Greece | 90 | 125 |  | Suriname | 90 | 119.21(66.80-189.64) |  | Argentina | 90 | 149 |  | Nigeria | 90 | 35.63(-12.62-106.08) |
| Netherlands | 91 | 139 |  | Turkmenistan | 91 | 115.06(59.46-183.03) |  | Kazakhstan | 91 | 138 |  | Paraguay | 91 | 33.36(-1.75-77.79) |
| Turkmenistan | 92 | 92 |  | Marshall Islands | 92 | 113.98(61.42-194.12) |  | Senegal | 92 | 108 |  | Haiti | 92 | 33.33(-8.20-97.68) |
| Kyrgyzstan | 93 | 105 |  | Mauritania | 93 | 112.48(43.17-216.28) |  | Bermuda | 93 | 151 |  | Bahamas | 93 | 31.89(-0.19-76.12) |
| Niger | 94 | 77 |  | Brunei Darussalam | 94 | 98.65(59.02-146.61) |  | Mongolia | 94 | 62 |  | Seychelles | 94 | 31.47(6.39-60.63) |
| Cambodia | 95 | 82 |  | Samoa | 95 | 97.11(25.35-189.97) |  | North Macedonia | 95 | 146 |  | United States of America | 95 | 30.26(12.31-62.76) |
| Lebanon | 96 | 106 |  | Iraq | 96 | 90.54(43.58-184.74) |  | Zambia | 96 | 80 |  | Bulgaria | 96 | 27.22(0.61-63.12) |
| Honduras | 97 | 94 |  | El Salvador | 97 | 89.99(42.51-165.77) |  | Kyrgyzstan | 97 | 121 |  | Burkina Faso | 97 | 26.98(-14.03-86.33) |
| Burkina Faso | 98 | 68 |  | Tajikistan | 98 | 84.71(40.57-133.58) |  | Dominica | 98 | 49 |  | United States Virgin Islands | 98 | 25.84(-6.71-71.04) |
| Guinea | 99 | 64 |  | China | 99 | 83.28(22.28-138.84) |  | Ethiopia | 99 | 169 |  | Cabo Verde | 99 | 25.07(-12.56-86.88) |
| Croatia | 100 | 146 |  | Dominica | 100 | 82.53(35.81-138.75) |  | Uzbekistan | 100 | 61 |  | Guatemala | 100 | 24.79(-4.70-75.62) |
| Republic of Moldova | 101 | 124 |  | Venezuela (Bolivarian Republic of) | 101 | 81.98(36.79-141.86) |  | Honduras | 101 | 134 |  | Micronesia (Federated States of) | 101 | 23.85(-9.24-79.38) |
| Fiji | 102 | 108 |  | Peru | 102 | 78.33(28.61-151.80) |  | Mali | 102 | 113 |  | Sri Lanka | 102 | 23.29(-21.13-75.05) |
| Nicaragua | 103 | 87 |  | Ecuador | 103 | 77.93(35.76-137.63) |  | Comoros | 103 | 76 |  | Sudan | 103 | 22.67(-25.11-94.52) |
| Kuwait | 104 | 89 |  | Mauritius | 104 | 73.90(44.06-111.59) |  | Peru | 104 | 119 |  | Bhutan | 104 | 22.38(-21.02-105.23) |
| Belgium | 105 | 143 |  | Bolivia (Plurinational State of) | 105 | 73.68(20.34-158.91) |  | United States of America | 105 | 91 |  | Equatorial Guinea | 105 | 22.14(-31.09-122.49) |
| Bosnia and Herzegovina | 106 | 145 |  | Bahamas | 106 | 72.85(30.80-130.80) |  | Nicaragua | 106 | 77 |  | Nauru | 106 | 21.97(-5.11-61.10) |
| Paraguay | 107 | 99 |  | Tonga | 107 | 67.56(19.05-149.37) |  | Cuba | 107 | 133 |  | Saint Lucia | 107 | 20.67(-1.90-52.89) |
| Lao People's Democratic Republic | 108 | 91 |  | Seychelles | 108 | 61.65(30.81-97.50) |  | Maldives | 108 | 127 |  | Brunei Darussalam | 108 | 20.03(-3.92-49.01) |
| United Arab Emirates | 109 | 62 |  | Bhutan | 109 | 57.41(1.59-163.98) |  | Lithuania | 109 | 154 |  | Bahrain | 109 | 19.91(-1.95-53.11) |
| Lithuania | 110 | 150 |  | Fiji | 110 | 55.29(15.85-114.36) |  | Czechia | 110 | 175 |  | Egypt | 110 | 19.34(-5.97-51.51) |
| Burundi | 111 | 101 |  | Morocco | 111 | 52.85(6.63-149.57) |  | Republic of Moldova | 111 | 88 |  | Afghanistan | 111 | 19.13(-15.00-82.92) |
| Liberia | 112 | 73 |  | Ukraine | 112 | 52.62(0.58-118.06) |  | Croatia | 112 | 174 |  | United Arab Emirates | 112 | 18.45(-16.02-58.58) |
| Zimbabwe | 113 | 56 |  | Trinidad and Tobago | 113 | 52.60(13.36-105.50) |  | Malaysia | 113 | 99 |  | Kuwait | 113 | 16.25(-5.69-47.29) |
| Central African Republic | 114 | 81 |  | Kyrgyzstan | 114 | 51.60(18.93-97.53) |  | Jamaica | 114 | 66 |  | Somalia | 114 | 15.40(-28.73-74.55) |
| Benin | 115 | 74 |  | Lebanon | 115 | 49.22(13.07-102.25) |  | Serbia | 115 | 163 |  | Russian Federation | 115 | 13.06(-5.31-33.73) |
| Trinidad and Tobago | 116 | 117 |  | Cyprus | 116 | 49.03(18.60-95.64) |  | Paraguay | 116 | 110 |  | Mali | 116 | 11.71(-22.24-69.90) |
| Armenia | 117 | 141 |  | Palau | 117 | 48.61(8.80-111.34) |  | Cabo Verde | 117 | 117 |  | Senegal | 117 | 11.19(-23.08-70.05) |
| Finland | 118 | 154 |  | South Africa | 118 | 48.51(25.31-77.15) |  | Lao People's Democratic Republic | 118 | 94 |  | Greece | 118 | 9.22(-8.61-28.93) |
| Congo | 119 | 76 |  | Tuvalu | 119 | 47.25(11.45-101.12) |  | Gambia | 119 | 63 |  | Morocco | 119 | 8.24(-24.49-76.73) |
| Austria | 120 | 149 |  | United States of America | 120 | 41.90(22.35-77.30) |  | Côte d'Ivoire | 120 | 73 |  | Tuvalu | 120 | 6.95(-19.05-46.08) |
| Switzerland | 121 | 162 |  | Saint Lucia | 121 | 41.76(15.26-79.62) |  | Colombia | 121 | 148 |  | Peru | 121 | 6.45(-23.22-50.31) |
| Latvia | 122 | 153 |  | Nauru | 122 | 40.41(9.23-85.44) |  | Armenia | 122 | 145 |  | Albania | 122 | 5.84(-16.46-40.02) |
| South Sudan | 123 | 107 |  | Guyana | 123 | 35.27(-0.11-84.21) |  | Belarus | 123 | 96 |  | Maldives | 123 | 3.12(-25.53-49.46) |
| Chad | 124 | 84 |  | Ethiopia | 124 | 34.61(-3.96-89.67) |  | Madagascar | 124 | 107 |  | Syrian Arab Republic | 124 | 2.27(-27.62-50.31) |
| Palestine | 125 | 104 |  | Sri Lanka | 125 | 34.46(-13.99-90.91) |  | Sri Lanka | 125 | 126 |  | Palestine | 125 | 2.12(-25.49-41.36) |
| Togo | 126 | 86 |  | Guam | 126 | 32.97(13.25-55.76) |  | Guinea | 126 | 103 |  | Burundi | 126 | 1.46(-35.06-58.61) |
| Mauritania | 127 | 112 |  | Saint Vincent and the Grenadines | 127 | 32.18(8.53-68.50) |  | Yemen | 127 | 98 |  | Canada | 127 | 1.42(-12.18-21.55) |
| Oman | 128 | 98 |  | Micronesia (Federated States of) | 128 | 31.27(-3.80-90.12) |  | Iran | 128 | 83 |  | Barbados | 128 | 1.32(-22.05-37.41) |
| Sweden | 129 | 151 |  | Brazil | 129 | 30.35(15.93-45.23) |  | Namibia | 129 | 92 |  | Grenada | 129 | 0.80(-18.79-27.24) |
| Mongolia | 130 | 113 |  | Colombia | 130 | 29.62(5.25-56.58) |  | Bhutan | 130 | 128 |  | Ecuador | 130 | 0.51(-23.31-34.24) |
| New Zealand | 131 | 140 |  | Tokelau | 131 | 25.98(-9.87-90.12) |  | Malawi | 131 | 109 |  | Kyrgyzstan | 131 | 0.47(-21.18-30.91) |
| Jamaica | 132 | 116 |  | American Samoa | 132 | 25.23(-12.11-72.92) |  | Ukraine | 132 | 65 |  | South Africa | 132 | -3.62(-18.68-14.97) |
| Uruguay | 133 | 138 |  | Rwanda | 133 | 25.19(-18.37-97.64) |  | Uruguay | 133 | 152 |  | Brazil | 133 | -4.06(-14.67-6.89) |
| Mauritius | 134 | 127 |  | Taiwan (Province of China) | 134 | 22.44(-0.84-49.60) |  | Tunisia | 134 | 82 |  | Mauritania | 134 | -4.30(-35.52-42.45) |
| Sierra Leone | 135 | 93 |  | Grenada | 135 | 22.20(-1.55-54.26) |  | Democratic Republic of the Congo | 135 | 97 |  | Uruguay | 135 | -4.53(-20.62-16.82) |
| Costa Rica | 136 | 115 |  | Chile | 136 | 17.40(0.46-34.63) |  | Togo | 136 | 93 |  | Monaco | 136 | -4.91(-40.93-51.12) |
| North Macedonia | 137 | 148 |  | Belarus | 137 | 14.68(-12.75-52.37) |  | Bosnia and Herzegovina | 137 | 164 |  | Chile | 137 | -4.97(-18.68-8.98) |
| Israel | 138 | 144 |  | Azerbaijan | 138 | 12.35(-11.01-37.26) |  | Indonesia | 138 | 81 |  | Cuba | 138 | -6.12(-23.03-16.71) |
| Guyana | 139 | 136 |  | Myanmar | 139 | 9.29(-25.67-63.77) |  | Angola | 139 | 122 |  | Tajikistan | 139 | -6.36(-28.74-18.42) |
| Estonia | 140 | 173 |  | Syrian Arab Republic | 140 | 8.43(-23.27-59.35) |  | Benin | 140 | 125 |  | Armenia | 140 | -6.50(-24.86-15.60) |
| Singapore | 141 | 142 |  | Canada | 141 | 8.21(-6.29-29.69) |  | Chile | 141 | 155 |  | Puerto Rico | 141 | -6.93(-21.79-13.53) |
| Denmark | 142 | 167 |  | Australia | 142 | 7.98(-8.01-24.18) |  | Sao Tome and Principe | 142 | 102 |  | Cyprus | 142 | -8.65(-27.30-19.92) |
| Albania | 143 | 157 |  | Antigua and Barbuda | 143 | 3.20(-14.45-26.87) |  | Pakistan | 143 | 60 |  | Romania | 143 | -9.06(-26.38-18.24) |
| Gabon | 144 | 120 |  | Niue | 144 | 3.06(-28.98-60.18) |  | United Republic of Tanzania | 144 | 111 |  | Colombia | 144 | -9.34(-26.38-9.51) |
| Norway | 145 | 170 |  | Republic of Moldova | 145 | 1.66(-15.42-26.99) |  | Germany | 145 | 188 |  | Honduras | 145 | -11.64(-50.05-35.92) |
| Ireland | 146 | 164 |  | Uruguay | 146 | 0.55(-16.39-23.03) |  | Niger | 146 | 168 |  | Jordan | 146 | -12.35(-31.89-14.37) |
| Eritrea | 147 | 111 |  | Argentina | 147 | 0.02(-15.50-20.53) |  | Botswana | 147 | 106 |  | Northern Mariana Islands | 147 | -12.52(-39.39-37.42) |
| Guinea-Bissau | 148 | 121 |  | Singapore | 148 | -0.43(-20.65-23.91) |  | New Zealand | 148 | 176 |  | Bolivia (Plurinational State of) | 148 | -12.84(-39.61-29.93) |
| Slovenia | 149 | 176 |  | Monaco | 149 | -3.12(-39.81-53.97) |  | Slovenia | 149 | 186 |  | Malta | 149 | -14.44(-29.48-4.93) |
| Namibia | 150 | 132 |  | Turkey | 150 | -6.45(-28.33-22.48) |  | Costa Rica | 150 | 123 |  | Australia | 150 | -15.65(-28.14-3.00) |
| Panama | 151 | 126 |  | New Zealand | 151 | -7.72(-18.57-6.03) |  | Lesotho | 151 | 34 |  | Azerbaijan | 151 | -15.71(-33.23-2.98) |
| Bahrain | 152 | 129 |  | Barbados | 152 | -8.31(-29.45-24.35) |  | Burundi | 152 | 161 |  | Cook Islands | 152 | -15.97(-45.07-27.84) |
| Qatar | 153 | 109 |  | Russian Federation | 153 | -9.67(-24.35-6.84) |  | Finland | 153 | 187 |  | Bosnia and Herzegovina | 153 | -16.49(-42.24-12.51) |
| Botswana | 154 | 134 |  | Israel | 154 | -16.55(-29.10-1.14) |  | Mozambique | 154 | 101 |  | Myanmar | 154 | -16.54(-43.24-25.06) |
| Lesotho | 155 | 114 |  | Malta | 155 | -17.00(-31.60-1.78) |  | Portugal | 155 | 181 |  | Niger | 155 | -16.67(-44.59-30.54) |
| Eswatini | 156 | 128 |  | Bulgaria | 156 | -18.82(-35.80-4.09) |  | Nigeria | 156 | 140 |  | Montenegro | 156 | -16.87(-32.17-4.53) |
| Gambia | 157 | 123 |  | Greece | 157 | -19.12(-32.32-4.52) |  | Monaco | 157 | 167 |  | Georgia | 157 | -17.68(-30.16-2.31) |
| Solomon Islands | 158 | 133 |  | United Kingdom | 158 | -21.84(-27.80-14.05) |  | Nepal | 158 | 141 |  | Latvia | 158 | -17.81(-33.75-6.45) |
| Montenegro | 159 | 174 |  | Kazakhstan | 159 | -22.71(-40.19-3.34) |  | Burkina Faso | 159 | 156 |  | Iraq | 159 | -21.48(-40.83-17.33) |
| Bahamas | 160 | 156 |  | Andorra | 160 | -22.75(-50.00-15.83) |  | United Kingdom | 160 | 179 |  | Singapore | 160 | -21.88(-37.75-2.78) |
| Micronesia | 161 | 166 |  | United States Virgin Islands | 161 | -26.53(-45.53-0.14) |  | Sierra Leone | 161 | 120 |  | France | 161 | -22.06(-35.54-5.80) |
| Equatorial Guinea | 162 | 131 |  | Iceland | 162 | -27.78(-37.60-9.54) |  | Greece | 162 | 166 |  | Antigua and Barbuda | 162 | -22.63(-35.86-4.89) |
| Kiribati | 163 | 152 |  | Albania | 163 | -29.36(-44.24-6.55) |  | Singapore | 163 | 180 |  | Qatar | 163 | -23.26(-42.06-8.02) |
| Samoa | 164 | 160 |  | North Macedonia | 164 | -29.38(-44.19-7.72) |  | Iceland | 164 | 183 |  | Andorra | 164 | -24.23(-50.95-13.62) |
| Suriname | 165 | 155 |  | France | 165 | -29.62(-41.79-14.94) |  | Spain | 165 | 185 |  | Kazakhstan | 165 | -24.72(-41.74-0.67) |
| Bhutan | 166 | 165 |  | Armenia | 166 | -30.06(-43.79-13.53) |  | Thailand | 166 | 75 |  | United Kingdom | 166 | -24.90(-30.63-17.41) |
| Brunei Darussalam | 167 | 161 |  | Cuba | 167 | -31.02(-43.44-14.24) |  | Republic of Korea | 167 | 191 |  | Lithuania | 167 | -25.60(-40.36-5.17) |
| Comoros | 168 | 158 |  | Montenegro | 168 | -31.87(-44.41-14.33) |  | Chad | 168 | 153 |  | Lebanon | 168 | -25.86(-43.83-0.48) |
| Barbados | 169 | 178 |  | Saint Kitts and Nevis | 169 | -31.89(-52.58-1.10) |  | Luxembourg | 169 | 202 |  | North Macedonia | 169 | -26.67(-42.05-4.18) |
| Vanuatu | 170 | 147 |  | Puerto Rico | 170 | -31.95(-42.82-16.99) |  | Australia | 170 | 177 |  | New Zealand | 170 | -29.23(-37.55-18.69) |
| Guam | 171 | 175 |  | Cook Islands | 171 | -35.84(-58.06-2.39) |  | Canada | 171 | 172 |  | Turkey | 171 | -29.76(-46.19-8.05) |
| Marshall Islands | 172 | 171 |  | Ireland | 172 | -37.46(-46.02-26.76) |  | India | 172 | 142 |  | Argentina | 172 | -30.27(-41.09-15.98) |
| Cabo Verde | 173 | 169 |  | San Marino | 173 | -38.38(-64.30-6.38) |  | Kenya | 173 | 124 |  | Serbia | 173 | -30.41(-45.28-11.45) |
| Cyprus | 174 | 177 |  | Northern Mariana Islands | 174 | -38.41(-57.33-3.25) |  | Cambodia | 174 | 158 |  | Bermuda | 174 | -30.43(-46.40-8.83) |
| Timor-Leste | 175 | 168 |  | Sweden | 175 | -42.15(-52.18-28.40) |  | Albania | 175 | 173 |  | Italy | 175 | -32.69(-40.63-22.24) |
| Belize | 176 | 163 |  | Serbia | 176 | -42.56(-54.83-26.91) |  | South Sudan | 176 | 157 |  | Japan | 176 | -34.04(-47.61-18.69) |
| United States Virgin Islands | 177 | 186 |  | Romania | 177 | -43.58(-54.33-26.65) |  | Uganda | 177 | 137 |  | San Marino | 177 | -35.42(-62.59-1.90) |
| Saint Lucia | 178 | 180 |  | Belgium | 178 | -48.14(-57.14-34.24) |  | Panama | 178 | 129 |  | Iceland | 178 | -37.31(-45.84-21.47) |
| Northern Mariana Islands | 179 | 188 |  | Austria | 179 | -48.26(-56.70-38.15) |  | Taiwan | 179 | 159 |  | Spain | 179 | -38.51(-47.31-29.39) |
| Tonga | 180 | 179 |  | Spain | 180 | -48.52(-55.89-40.88) |  | Ireland | 180 | 190 |  | Rwanda | 180 | -39.51(-60.55-4.49) |
| American Samoa | 181 | 182 |  | Italy | 181 | -50.20(-56.08-42.46) |  | Djibouti | 181 | 144 |  | Portugal | 181 | -39.67(-47.90-29.56) |
| Maldives | 182 | 172 |  | Luxembourg | 182 | -51.71(-60.90-39.99) |  | Eritrea | 182 | 147 |  | Slovakia | 182 | -43.18(-54.38-26.21) |
| Djibouti | 183 | 159 |  | Japan | 183 | -52.30(-62.11-41.20) |  | Israel | 183 | 195 |  | Belgium | 183 | -44.91(-54.47-30.14) |
| Luxembourg | 184 | 195 |  | Slovakia | 184 | -52.50(-61.86-38.32) |  | Switzerland | 184 | 204 |  | Austria | 184 | -44.97(-53.95-34.21) |
| Grenada | 185 | 185 |  | Bermuda | 185 | -52.63(-63.51-37.93) |  | China | 185 | 136 |  | Ireland | 185 | -45.17(-52.68-35.79) |
| Saint Vincent and the Grenadines | 186 | 183 |  | Portugal | 186 | -52.97(-59.38-45.08) |  | Denmark | 186 | 199 |  | Croatia | 186 | -46.51(-56.50-31.72) |
| Seychelles | 187 | 184 |  | Latvia | 187 | -53.60(-62.60-39.90) |  | Bangladesh | 187 | 143 |  | Ethiopia | 187 | -46.95(-62.15-25.25) |
| Iceland | 188 | 192 |  | Netherlands | 188 | -54.54(-61.63-44.69) |  | Belgium | 188 | 194 |  | Greenland | 188 | -47.08(-63.59-26.74) |
| Malta | 189 | 191 |  | Bosnia and Herzegovina | 189 | -55.75(-69.39-40.38) |  | Norway | 189 | 203 |  | Sweden | 189 | -47.61(-56.69-35.16) |
| Nauru | 190 | 187 |  | Norway | 190 | -55.90(-61.67-44.97) |  | Austria | 190 | 196 |  | Netherlands | 190 | -48.09(-56.19-36.84) |
| Greenland | 191 | 198 |  | Georgia | 191 | -56.13(-62.78-45.47) |  | Malta | 191 | 184 |  | Saint Kitts and Nevis | 191 | -48.21(-63.94-24.80) |
| Saint Kitts and Nevis | 192 | 196 |  | Lithuania | 192 | -57.01(-65.54-45.20) |  | Cyprus | 192 | 182 |  | Czechia | 192 | -48.83(-58.31-36.84) |
| Cook Islands | 193 | 197 |  | Finland | 193 | -58.01(-64.30-49.16) |  | Sudan | 193 | 35 |  | Israel | 193 | -52.01(-59.23-41.84) |
| Antigua and Barbuda | 194 | 193 |  | Greenland | 194 | -59.21(-71.94-43.54) |  | Democratic People's Republic of Korea | 194 | 160 |  | Republic of Korea | 194 | -52.63(-65.82-33.43) |
| Palau | 195 | 190 |  | Czechia | 195 | -59.28(-66.82-49.74) |  | Andorra | 195 | 189 |  | Finland | 195 | -54.24(-61.09-44.60) |
| Sao Tome and Principe | 196 | 181 |  | Denmark | 196 | -59.28(-65.60-51.39) |  | Timor-Leste | 196 | 170 |  | Slovenia | 196 | -55.45(-63.74-44.36) |
| Dominica | 197 | 189 |  | Croatia | 197 | -63.20(-70.08-53.03) |  | Zimbabwe | 197 | 74 |  | Estonia | 197 | -57.19(-65.82-45.11) |
| Bermuda | 198 | 199 |  | Republic of Korea | 198 | -63.98(-74.02-49.39) |  | Sweden | 198 | 198 |  | Denmark | 198 | -57.39(-64.01-49.14) |
| Tuvalu | 199 | 194 |  | Switzerland | 199 | -64.33(-69.76-55.96) |  | Italy | 199 | 193 |  | Norway | 199 | -60.25(-65.45-50.40) |
| Andorra | 200 | 200 |  | Germany | 200 | -66.83(-70.79-61.24) |  | Netherlands | 200 | 200 |  | Germany | 200 | -61.04(-65.69-54.48) |
| Monaco | 201 | 201 |  | Slovenia | 201 | -66.90(-73.06-58.66) |  | France | 201 | 192 |  | Hungary | 201 | -62.24(-69.27-50.47) |
| Niue | 202 | 202 |  | Poland | 202 | -68.83(-73.58-61.96) |  | San Marino | 202 | 197 |  | Poland | 202 | -62.78(-68.45-54.59) |
| Tokelau | 203 | 203 |  | Estonia | 203 | -70.20(-76.20-61.78) |  | Japan | 203 | 201 |  | Switzerland | 203 | -66.15(-71.30-58.21) |
| San Marino | 204 | 204 |  | Hungary | 204 | -71.89(-77.12-63.13) |  | Viet Nam | 204 | 178 |  | Luxembourg | 204 | -67.69(-73.84-59.86) |

HSBP- high systolic blood pressure; UI- uncertainty interval.

**Table S4.** Ranks of High BMI-related DALYs- DALY rates- and the changes in the youth and young adults of 204 countries/territories- 1990-2021

| **The rank of DALYs** | | |  | **The rank of percentage change in DALYs** | | |  | **The rank of DALY rates** | | |  | **The rank of percentage change in DALY rates** | | |
| --- | --- | --- | --- | --- | --- | --- | --- | --- | --- | --- | --- | --- | --- | --- |
| **(Descending)** | | |  | **(Descending) 1990-2021** | | |  | **(Descending)** | | |  | **(Descending) 1990-2021** | | |
| **Country or region** | **Rank** | |  | **Country or region** | **Rank** | **Value (95%UI)** |  | **Country or region** | **Rank** | |  | **Country or region** | **Rank** | **Value (95%UI)** |
|  | **1990** | **2021** |  |  |  |  |  |  | **1990** | **2021** |  |  |  |  |
| People's Republic of China | 1 | 1 |  | State of Qatar | 1 | 891.67(718.20-1119.59) |  | Republic of Nauru | 1 | 1 |  | Republic of Zimbabwe | 1 | 256.79(156.47-374.71) |
| United States of America | 2 | 3 |  | United Arab Emirates | 2 | 682.61(528.50-844.80) |  | Republic of Kiribati | 2 | 3 |  | Kingdom of Lesotho | 2 | 196.14(110.97-309.32) |
| Republic of India | 3 | 2 |  | Republic of Equatorial Guinea | 3 | 618.57(397.35-1014.94) |  | Republic of the Marshall Islands | 3 | 2 |  | People's Republic of Bangladesh | 3 | 194.19(111.17-311.28) |
| Federative Republic of Brazil | 4 | 7 |  | Kingdom of Saudi Arabia | 4 | 577.69(413.59-844.65) |  | Tuvalu | 4 | 8 |  | Kingdom of Thailand | 4 | 191.93(126.42-274.70) |
| Russian Federation | 5 | 13 |  | Islamic Republic of Pakistan | 5 | 530.62(393.15-706.31) |  | Federated States of Micronesia | 5 | 5 |  | People's Republic of China | 5 | 189.65(132.04-232.37) |
| United Mexican States | 6 | 5 |  | Islamic Republic of Afghanistan | 6 | 506.52(366.24-741.00) |  | Cook Islands | 6 | 12 |  | State of Libya | 6 | 182.28(124.59-247.87) |
| Arab Republic of Egypt | 7 | 6 |  | Republic of Djibouti | 7 | 499.91(299.45-775.82) |  | Republic of Palau | 7 | 4 |  | Islamic Republic of Pakistan | 7 | 160.17(103.45-232.65) |
| Republic of Indonesia | 8 | 8 |  | Republic of Cameroon | 8 | 499.50(367.06-676.08) |  | American Samoa | 8 | 6 |  | Socialist Republic of Viet Nam | 8 | 155.26(65.78-254.49) |
| Republic of South Africa | 9 | 15 |  | Republic of Zimbabwe | 9 | 470.35(309.98-658.84) |  | Republic of Fiji | 9 | 10 |  | Republic of Tunisia | 9 | 144.26(102.43-199.23) |
| Federal Republic of Germany | 10 | 26 |  | Republic of Yemen | 10 | 452.60(297.58-667.27) |  | Republic of Niue | 10 | 7 |  | Kingdom of Saudi Arabia | 10 | 143.14(84.26-238.91) |
| Republic of Turkey | 11 | 16 |  | Republic of Mozambique | 11 | 449.47(276.40-674.11) |  | Tokelau | 11 | 9 |  | Kingdom of Eswatini | 11 | 132.12(62.12-230.15) |
| United Kingdom of Great Britain and Northern Ireland | 12 | 19 |  | Sultanate of Oman | 12 | 449.37(326.18-595.12) |  | Northern Mariana Islands | 12 | 17 |  | Republic of Panama | 12 | 128.52(89.77-174.71) |
| Islamic Republic of Pakistan | 13 | 4 |  | Republic of Angola | 13 | 448.84(304.35-641.30) |  | Independent State of Samoa | 13 | 11 |  | Islamic Republic of Iran | 13 | 126.91(90.95-169.42) |
| Republic of the Philippines | 14 | 12 |  | Republic of Uganda | 14 | 420.52(264.54-609.16) |  | Arab Republic of Egypt | 14 | 20 |  | Taiwan (Province of China) | 14 | 125.01(80.79-166.62) |
| Japan | 15 | 35 |  | Republic of Benin | 15 | 417.85(298.32-562.11) |  | United States Virgin Islands | 15 | 22 |  | Republic of Botswana | 15 | 121.62(46.67-233.06) |
| Republic of Poland | 16 | 48 |  | Republic of Kenya | 16 | 416.80(301.04-534.12) |  | Kingdom of Tonga | 16 | 15 |  | Republic of Mozambique | 16 | 116.76(48.48-205.37) |
| Ukraine | 17 | 27 |  | Republic of the Gambia | 17 | 415.76(279.57-590.90) |  | Guam | 17 | 24 |  | Republic of the Congo | 17 | 116.63(49.96-206.11) |
| Federal Republic of Nigeria | 18 | 10 |  | Hashemite Kingdom of Jordan | 18 | 412.50(323.00-512.88) |  | Republic of Vanuatu | 18 | 14 |  | Dominican Republic | 18 | 115.16(75.48-159.35) |
| Islamic Republic of Iran | 19 | 14 |  | Republic of the Congo | 19 | 406.65(250.74-615.92) |  | Puerto Rico | 19 | 36 |  | Republic of Indonesia | 19 | 111.11(62.73-171.65) |
| Republic of the Union of Myanmar | 20 | 29 |  | State of Libya | 20 | 404.11(301.07-521.22) |  | Republic of Trinidad and Tobago | 20 | 18 |  | Democratic Republic of Sao Tome and Principe | 20 | 110.60(53.35-198.05) |
| People's Republic of Bangladesh | 21 | 11 |  | Republic of Maldives | 21 | 388.80(272.96-552.46) |  | Republic of South Africa | 21 | 50 |  | Republic of Kenya | 21 | 109.04(62.21-156.49) |
| Argentine Republic | 22 | 30 |  | Republic of Sierra Leone | 22 | 383.69(261.53-563.18) |  | Saint Kitts and Nevis | 22 | 67 |  | Republic of Sierra Leone | 22 | 107.16(54.84-184.03) |
| Republic of Colombia | 23 | 24 |  | People's Republic of Bangladesh | 23 | 379.62(244.27-570.49) |  | Hungary | 23 | 90 |  | Federal Democratic Republic of Nepal | 23 | 105.67(46.07-183.47) |
| Republic of Iraq | 24 | 17 |  | Democratic Republic of the Congo | 24 | 379.43(241.57-566.98) |  | Commonwealth of the Bahamas | 24 | 26 |  | Republic of Mauritius | 24 | 105.55(74.02-146.67) |
| Federal Democratic Republic of Ethiopia | 25 | 33 |  | Republic of Chad | 25 | 376.89(245.72-568.65) |  | Republic of Guyana | 25 | 19 |  | State of Eritrea | 25 | 105.33(24.89-213.99) |
| Kingdom of Morocco | 26 | 22 |  | United Republic of Tanzania | 26 | 360.43(223.60-502.03) |  | Syrian Arab Republic | 26 | 33 |  | Central African Republic | 26 | 103.57(43.11-196.10) |
| French Republic | 27 | 40 |  | Togolese Republic | 27 | 359.20(228.67-523.97) |  | Republic of Iraq | 27 | 44 |  | Commonwealth of Dominica | 27 | 102.10(72.06-139.82) |
| Republic of Italy | 28 | 54 |  | Republic of Botswana | 28 | 358.66(203.56-589.32) |  | State of Kuwait | 28 | 21 |  | People's Democratic Republic of Algeria | 28 | 102.06(58.56-153.04) |
| Kingdom of Thailand | 29 | 25 |  | Republic of Zambia | 29 | 357.34(219.78-525.79) |  | Grenada | 29 | 37 |  | Democratic People's Republic of Korea | 29 | 100.83(50.13-175.36) |
| Republic of Korea | 30 | 45 |  | Kingdom of Lesotho | 30 | 356.54(225.24-531.03) |  | State of Qatar | 30 | 30 |  | Solomon Islands | 30 | 100.23(46.32-204.66) |
| Bolivarian Republic of Venezuela | 31 | 34 |  | Republic of C么te d'Ivoire | 31 | 354.33(251.96-495.97) |  | Solomon Islands | 31 | 16 |  | Republic of India | 31 | 99.79(60.49-153.93) |
| Kingdom of Spain | 32 | 46 |  | Democratic Republic of Sao Tome and Principe | 32 | 345.89(224.67-531.04) |  | Kingdom of Bahrain | 32 | 28 |  | Republic of Suriname | 32 | 99.75(67.01-145.09) |
| Kingdom of Saudi Arabia | 33 | 9 |  | Republic of Liberia | 33 | 344.93(236.95-491.93) |  | Kingdom of Saudi Arabia | 33 | 13 |  | Republic of Singapore | 33 | 97.23(49.29-136.96) |
| Republic of Sudan | 34 | 18 |  | State of Eritrea | 34 | 343.64(169.83-578.42) |  | Greenland | 34 | 109 |  | Sultanate of Oman | 34 | 96.96(52.79-149.21) |
| Romania | 35 | 71 |  | State of Kuwait | 35 | 334.98(268.61-412.38) |  | Republic of Guatemala | 35 | 41 |  | Republic of Djibouti | 35 | 94.50(29.51-183.95) |
| People's Democratic Republic of Algeria | 36 | 21 |  | Solomon Islands | 36 | 326.97(212.02-549.67) |  | Islamic Republic of Afghanistan | 36 | 31 |  | Republic of the Gambia | 36 | 94.43(43.09-160.46) |
| Canada | 37 | 49 |  | Central African Republic | 37 | 326.79(200.04-520.78) |  | United Arab Emirates | 37 | 25 |  | Republic of Uganda | 37 | 94.13(35.96-164.49) |
| Syrian Arab Republic | 38 | 52 |  | Republic of Mali | 38 | 326.76(222.75-481.69) |  | United Mexican States | 38 | 27 |  | Republic of C么te d'Ivoire | 38 | 91.68(48.49-151.43) |
| Republic of Uzbekistan | 39 | 31 |  | Kingdom of Bahrain | 39 | 323.68(258.43-407.75) |  | Saint Lucia | 39 | 35 |  | United Republic of Tanzania | 39 | 91.19(34.37-149.98) |
| Democratic Republic of the Congo | 40 | 20 |  | Independent State of Papua New Guinea | 40 | 322.38(221.83-484.75) |  | Republic of Bulgaria | 40 | 53 |  | Democratic Republic of the Congo | 40 | 90.32(35.59-164.77) |
| Republic of Peru | 41 | 41 |  | Republic of Ghana | 41 | 320.33(211.45-469.69) |  | Barbados | 41 | 54 |  | Jamaica | 41 | 89.67(59.76-133.89) |
| Australia | 42 | 53 |  | Belize | 42 | 318.20(261.58-406.39) |  | Independent State of Papua New Guinea | 42 | 32 |  | Togolese Republic | 42 | 87.10(33.92-154.24) |
| Hungary | 43 | 99 |  | Republic of Guinea | 43 | 310.91(194.17-472.16) |  | Georgia | 43 | 78 |  | Republic of Vanuatu | 43 | 87.01(36.18-153.67) |
| Republic of Kazakhstan | 44 | 67 |  | Federal Republic of Nigeria | 44 | 310.38(215.77-434.77) |  | United States of America | 44 | 48 |  | Ukraine | 44 | 86.60(53.21-138.19) |
| Malaysia | 45 | 38 |  | Burkina Faso | 45 | 302.69(199.93-442.19) |  | New Zealand | 45 | 94 |  | United Arab Emirates | 45 | 86.22(49.55-124.81) |
| Republic of Chile | 46 | 63 |  | Palestine | 46 | 300.37(215.96-390.74) |  | Republic of Sudan | 46 | 52 |  | Republic of Yemen | 46 | 84.61(32.82-156.33) |
| Republic of Cuba | 47 | 84 |  | Republic of Vanuatu | 47 | 297.89(189.74-439.71) |  | Republic of El Salvador | 47 | 43 |  | Republic of Uzbekistan | 47 | 84.20(58.49-120.31) |
| United Republic of Tanzania | 48 | 28 |  | Republic of Madagascar | 48 | 293.66(170.39-469.14) |  | Bolivarian Republic of Venezuela | 48 | 38 |  | Republic of Palau | 48 | 83.25(44.67-140.37) |
| Islamic Republic of Afghanistan | 49 | 23 |  | Kingdom of Eswatini | 49 | 292.15(173.88-457.76) |  | Belize | 49 | 47 |  | Republic of Malta | 49 | 83.14(54.96-116.20) |
| Republic of Ecuador | 50 | 57 |  | Federal Democratic Republic of Nepal | 50 | 277.47(168.10-420.27) |  | Lebanese Republic | 50 | 58 |  | Republic of Liberia | 50 | 82.83(38.46-143.23) |
| Democratic Socialist Republic of Sri Lanka | 51 | 68 |  | Republic of Panama | 51 | 272.86(209.63-348.21) |  | Antigua and Barbuda | 51 | 75 |  | Republic of Cabo Verde | 51 | 82.21(36.66-143.89) |
| Republic of Ghana | 52 | 36 |  | Federal Republic of Somalia | 52 | 269.96(151.65-410.61) |  | Republic of Poland | 52 | 142 |  | Democratic Republic of Timor-Leste | 52 | 82.09(17.54-186.04) |
| Taiwan (Province of China) | 53 | 69 |  | Republic of Sudan | 53 | 267.37(159.62-410.32) |  | Republic of Seychelles | 53 | 46 |  | Brunei Darussalam | 53 | 81.87(46.87-119.44) |
| Republic of Guatemala | 54 | 42 |  | Republic of Guinea-Bissau | 54 | 262.84(160.86-429.80) |  | Saint Vincent and the Grenadines | 54 | 42 |  | Republic of Cyprus | 54 | 80.76(51.00-118.07) |
| Republic of Bulgaria | 55 | 101 |  | Islamic Republic of Iran | 55 | 262.58(205.11-330.51) |  | Republic of Haiti | 55 | 57 |  | Saint Vincent and the Grenadines | 55 | 79.00(54.36-111.74) |
| Socialist Republic of Viet Nam | 56 | 44 |  | Republic of Haiti | 56 | 261.31(178.19-382.31) |  | Brunei Darussalam | 56 | 40 |  | Independent State of Samoa | 56 | 78.22(34.18-130.01) |
| Kingdom of the Netherlands | 57 | 90 |  | Republic of India | 57 | 257.05(186.81-353.81) |  | Hashemite Kingdom of Jordan | 57 | 63 |  | Union of the Comoros | 57 | 78.05(19.49-185.34) |
| Czech Republic | 58 | 113 |  | Republic of Cabo Verde | 58 | 249.22(161.92-367.45) |  | Republic of Estonia | 58 | 129 |  | Republic of Cameroon | 58 | 76.85(37.78-128.94) |
| Republic of Cameroon | 59 | 32 |  | Lao People's Democratic Republic | 59 | 247.79(137.47-407.66) |  | Palestine | 59 | 72 |  | Republic of Angola | 59 | 76.51(30.04-138.40) |
| Federal Democratic Republic of Nepal | 60 | 47 |  | Republic of Malawi | 60 | 246.72(160.59-350.89) |  | Republic of Mauritius | 60 | 29 |  | Mongolia | 60 | 74.74(44.24-115.58) |
| Portuguese Republic | 61 | 97 |  | Socialist Republic of Viet Nam | 61 | 243.62(123.17-377.21) |  | Kingdom of Morocco | 61 | 55 |  | Republic of Namibia | 61 | 74.52(25.62-141.70) |
| Republic of Kenya | 62 | 37 |  | People's Democratic Republic of Algeria | 62 | 240.50(167.20-326.40) |  | United Kingdom of Great Britain and Northern Ireland | 62 | 69 |  | Republic of Korea | 62 | 74.17(32.19-124.97) |
| Republic of Azerbaijan | 63 | 82 |  | Republic of Honduras | 63 | 240.21(165.59-324.37) |  | Gabonese Republic | 63 | 56 |  | Republic of Costa Rica | 63 | 73.81(54.70-99.09) |
| Republic of Côte d'Ivoire | 64 | 43 |  | Republic of the Niger | 64 | 239.68(148.80-363.36) |  | Republic of Turkey | 64 | 88 |  | Republic of Guyana | 64 | 73.25(40.37-114.67) |
| Republic of Serbia | 65 | 109 |  | Republic of Guatemala | 65 | 236.64(177.66-337.63) |  | Bermuda | 65 | 89 |  | State of Kuwait | 65 | 73.18(46.75-103.99) |
| Republic of Belarus | 66 | 100 |  | Gabonese Republic | 66 | 229.26(138.73-355.93) |  | Commonwealth of Dominica | 66 | 34 |  | United Mexican States | 66 | 72.55(56.89-91.75) |
| Dominican Republic | 67 | 59 |  | Democratic Republic of Timor-Leste | 67 | 226.85(110.98-413.44) |  | Romania | 67 | 87 |  | Republic of Zambia | 67 | 71.47(19.89-134.63) |
| Hellenic Republic | 68 | 103 |  | Republic of Namibia | 68 | 226.16(134.77-351.70) |  | Federative Republic of Brazil | 68 | 82 |  | Republic of Trinidad and Tobago | 68 | 71.32(39.92-110.72) |
| Republic of Yemen | 69 | 39 |  | Republic of Nicaragua | 69 | 219.82(178.15-285.92) |  | Republic of Ecuador | 69 | 84 |  | Republic of Seychelles | 69 | 70.98(41.43-99.83) |
| Georgia | 70 | 130 |  | Union of the Comoros | 70 | 219.30(114.29-411.71) |  | Montenegro | 70 | 93 |  | Republic of Moldova | 70 | 70.12(46.40-103.85) |
| Republic of Haiti | 71 | 58 |  | Dominican Republic | 71 | 218.49(159.75-283.90) |  | Republic of Suriname | 71 | 45 |  | Kingdom of Tonga | 71 | 69.44(39.67-119.04) |
| Democratic People's Republic of Korea | 72 | 74 |  | Republic of Paraguay | 72 | 212.78(159.16-270.93) |  | Republic of Nicaragua | 72 | 62 |  | Gabonese Republic | 72 | 68.96(22.51-133.96) |
| Republic of El Salvador | 73 | 79 |  | Republic of Tunisia | 73 | 209.02(156.10-278.56) |  | Republic of Equatorial Guinea | 73 | 70 |  | Republic of Ghana | 73 | 68.71(25.00-128.65) |
| Kingdom of Belgium | 74 | 107 |  | Republic of Indonesia | 74 | 208.03(137.45-296.37) |  | Russian Federation | 74 | 92 |  | Republic of the Marshall Islands | 74 | 68.60(33.71-119.43) |
| Plurinational State of Bolivia | 75 | 78 |  | Republic of Senegal | 75 | 207.68(141.18-304.47) |  | Plurinational State of Bolivia | 75 | 118 |  | Republic of Niue | 75 | 68.28(27.85-126.84) |
| Puerto Rico | 76 | 120 |  | Kingdom of Cambodia | 76 | 207.56(112.22-344.22) |  | Argentine Republic | 76 | 110 |  | Republic of Benin | 76 | 68.20(29.38-115.06) |
| Republic of Tunisia | 77 | 72 |  | Malaysia | 77 | 204.99(160.54-259.83) |  | Republic of Latvia | 77 | 114 |  | Lao People's Democratic Republic | 77 | 67.51(14.37-144.51) |
| Republic of Madagascar | 78 | 61 |  | Republic of Iraq | 78 | 204.07(146.00-291.51) |  | Republic of Cuba | 78 | 85 |  | Kingdom of Morocco | 78 | 67.47(31.69-119.44) |
| Independent State of Papua New Guinea | 79 | 60 |  | Brunei Darussalam | 79 | 200.98(143.06-263.16) |  | Sultanate of Oman | 79 | 49 |  | Democratic Socialist Republic of Sri Lanka | 79 | 67.21(25.96-116.28) |
| Republic of Uganda | 80 | 50 |  | Republic of the Philippines | 80 | 199.49(148.19-255.60) |  | Slovak Republic | 80 | 143 |  | Hellenic Republic | 80 | 66.18(44.84-89.66) |
| Republic of Rwanda | 81 | 102 |  | Lebanese Republic | 81 | 198.65(139.55-271.06) |  | State of Libya | 81 | 23 |  | Bolivarian Republic of Venezuela | 81 | 66.07(40.08-98.55) |
| Republic of Senegal | 82 | 76 |  | Republic of Cyprus | 82 | 194.89(146.34-255.76) |  | Republic of Azerbaijan | 82 | 106 |  | Republic of Nicaragua | 82 | 65.86(44.25-100.14) |
| Republic of Mali | 83 | 65 |  | Republic of Uzbekistan | 83 | 194.80(153.65-252.58) |  | North Macedonia | 83 | 119 |  | Kingdom of Bhutan | 83 | 65.45(24.91-127.01) |
| Republic of Angola | 84 | 51 |  | Republic of Burundi | 84 | 192.40(105.43-311.15) |  | Czech Republic | 84 | 157 |  | Republic of Belarus | 84 | 65.36(33.44-100.87) |
| Slovak Republic | 85 | 129 |  | Arab Republic of Egypt | 85 | 177.41(125.52-236.91) |  | Dominican Republic | 85 | 39 |  | American Samoa | 85 | 64.68(30.75-109.28) |
| Swiss Confederation | 86 | 116 |  | Republic of Suriname | 86 | 163.30(120.14-223.06) |  | Australia | 86 | 102 |  | Republic of the Philippines | 86 | 64.28(36.15-95.06) |
| Federal Republic of Somalia | 87 | 70 |  | Republic of Costa Rica | 87 | 157.51(129.21-194.97) |  | Republic of Liberia | 87 | 60 |  | Kingdom of Cambodia | 87 | 63.49(12.81-136.14) |
| Republic of Zambia | 88 | 62 |  | Republic of Singapore | 88 | 151.39(90.29-202.04) |  | Principality of Monaco | 88 | 112 |  | Republic of Guinea | 88 | 63.38(16.96-127.49) |
| Republic of Malawi | 89 | 75 |  | Islamic Republic of Mauritania | 89 | 149.75(94.60-225.12) |  | Republic of Iceland | 89 | 117 |  | Independent State of Papua New Guinea | 89 | 63.37(24.47-126.16) |
| Republic of Tajikistan | 90 | 89 |  | Mongolia | 90 | 149.58(106.01-207.91) |  | Islamic Republic of Mauritania | 90 | 133 |  | Tokelau | 90 | 63.16(27.81-117.29) |
| Republic of Mozambique | 91 | 56 |  | United Mexican States | 91 | 149.23(126.62-176.97) |  | Republic of Costa Rica | 91 | 68 |  | Republic of El Salvador | 91 | 63.07(37.36-104.56) |
| Kingdom of Sweden | 92 | 115 |  | People's Republic of China | 92 | 143.84(95.34-179.80) |  | Turkmenistan | 92 | 80 |  | Malaysia | 92 | 62.82(39.09-92.10) |
| New Zealand | 93 | 119 |  | Democratic People's Republic of Korea | 93 | 142.19(81.04-232.06) |  | Federal Republic of Germany | 93 | 155 |  | Belize | 93 | 62.08(40.13-96.25) |
| Hashemite Kingdom of Jordan | 94 | 64 |  | Kingdom of Thailand | 94 | 138.65(85.10-206.32) |  | Republic of the Union of Myanmar | 94 | 166 |  | Turkmenistan | 94 | 61.87(32.72-99.48) |
| Republic of Austria | 95 | 124 |  | Republic of Tajikistan | 95 | 136.58(92.12-186.07) |  | Republic of Honduras | 95 | 108 |  | Republic of Haiti | 95 | 60.22(23.36-113.88) |
| State of Libya | 96 | 66 |  | Kingdom of Morocco | 96 | 136.49(85.96-209.87) |  | Republic of Tajikistan | 96 | 128 |  | Republic of Paraguay | 96 | 60.22(32.76-90.01) |
| Republic of Croatia | 97 | 142 |  | Republic of Kiribati | 97 | 136.45(81.86-214.73) |  | Republic of Croatia | 97 | 151 |  | Republic of Guinea-Bissau | 97 | 59.47(14.65-132.85) |
| Republic of Honduras | 98 | 81 |  | Republic of South Sudan | 98 | 136.05(67.88-237.28) |  | Republic of Chile | 98 | 95 |  | Republic of Chad | 98 | 59.07(15.32-123.04) |
| Republic of Finland | 99 | 128 |  | Republic of the Marshall Islands | 99 | 133.16(84.91-203.45) |  | Republic of Cameroon | 99 | 71 |  | Republic of Malawi | 99 | 58.32(18.99-105.88) |
| Republic of Moldova | 100 | 125 |  | Republic of Ecuador | 100 | 132.36(89.83-182.74) |  | Portuguese Republic | 100 | 98 |  | Saint Lucia | 100 | 58.22(37.91-83.25) |
| Kyrgyz Republic | 101 | 112 |  | Jamaica | 101 | 130.30(93.97-183.99) |  | Republic of Moldova | 101 | 79 |  | Islamic Republic of Afghanistan | 101 | 56.31(20.16-116.74) |
| Republic of Nicaragua | 102 | 86 |  | Plurinational State of Bolivia | 102 | 130.05(72.73-198.53) |  | Republic of Guinea-Bissau | 102 | 83 |  | Republic of Equatorial Guinea | 102 | 55.99(7.96-142.03) |
| Bosnia and Herzegovina | 103 | 150 |  | Republic of Peru | 103 | 122.44(75.47-180.04) |  | People's Democratic Republic of Algeria | 103 | 59 |  | Federal Republic of Nigeria | 103 | 55.80(19.88-103.02) |
| Turkmenistan | 104 | 111 |  | Turkmenistan | 104 | 119.32(79.82-170.28) |  | Republic of Kazakhstan | 104 | 120 |  | Kingdom of Bahrain | 104 | 54.37(30.59-85.00) |
| Lebanese Republic | 105 | 91 |  | Independent State of Samoa | 105 | 113.84(61.00-175.98) |  | Jamaica | 105 | 65 |  | Kingdom of Spain | 105 | 53.68(30.17-76.98) |
| State of Kuwait | 106 | 77 |  | Kingdom of Bhutan | 106 | 112.81(60.66-191.99) |  | Republic of Slovenia | 106 | 156 |  | Guam | 106 | 52.87(36.90-72.69) |
| Republic of the Niger | 107 | 87 |  | Republic of Colombia | 107 | 110.27(78.68-148.20) |  | Republic of Finland | 107 | 135 |  | Republic of Maldives | 107 | 52.68(16.50-103.80) |
| United Arab Emirates | 108 | 55 |  | Republic of Seychelles | 108 | 110.22(73.90-145.69) |  | Republic of Colombia | 108 | 103 |  | Republic of Fiji | 108 | 52.55(22.31-96.15) |
| State of Israel | 109 | 118 |  | Republic of El Salvador | 109 | 102.30(70.41-153.77) |  | Kyrgyz Republic | 109 | 130 |  | Republic of Madagascar | 109 | 52.26(4.58-120.14) |
| Burkina Faso | 110 | 83 |  | Commonwealth of the Bahamas | 110 | 97.21(64.79-138.04) |  | Grand Duchy of Luxembourg | 110 | 154 |  | Republic of Sudan | 110 | 51.45(7.03-110.38) |
| Kingdom of Norway | 111 | 138 |  | Bolivarian Republic of Venezuela | 111 | 94.13(63.75-132.10) |  | Republic of Serbia | 111 | 134 |  | Republic of South Sudan | 111 | 51.40(7.68-116.33) |
| Kingdom of Denmark | 112 | 136 |  | Kyrgyz Republic | 112 | 90.37(56.67-132.06) |  | Mongolia | 112 | 81 |  | French Republic | 112 | 51.05(23.37-77.30) |
| Republic of Paraguay | 113 | 93 |  | State of Israel | 113 | 88.75(60.23-113.88) |  | Ukraine | 113 | 73 |  | Commonwealth of the Bahamas | 113 | 50.48(25.74-81.63) |
| Kingdom of Cambodia | 114 | 94 |  | Republic of Mauritius | 114 | 88.22(59.35-125.88) |  | Kingdom of Eswatini | 114 | 51 |  | United States of America | 114 | 50.47(30.30-86.69) |
| Republic of Costa Rica | 115 | 110 |  | Saint Lucia | 115 | 85.88(62.02-115.29) |  | Ireland | 115 | 148 |  | Lebanese Republic | 115 | 48.37(19.01-84.35) |
| Republic of Zimbabwe | 116 | 73 |  | Taiwan (Province of China) | 116 | 84.00(47.84-118.03) |  | Republic of Uzbekistan | 116 | 76 |  | Burkina Faso | 116 | 48.34(10.48-99.72) |
| Republic of Guinea | 117 | 85 |  | Democratic Socialist Republic of Sri Lanka | 117 | 82.36(37.37-135.88) |  | Republic of the Philippines | 117 | 91 |  | Republic of Colombia | 117 | 47.06(24.97-73.60) |
| Ireland | 118 | 134 |  | Federative Republic of Brazil | 118 | 79.72(51.22-103.38) |  | Republic of Armenia | 118 | 138 |  | Hashemite Kingdom of Jordan | 118 | 46.73(21.11-75.47) |
| Republic of Armenia | 119 | 146 |  | Commonwealth of Dominica | 119 | 79.22(52.57-112.67) |  | Republic of Peru | 119 | 136 |  | United Kingdom of Great Britain and Northern Ireland | 119 | 46.14(30.60-68.01) |
| Republic of Lithuania | 120 | 155 |  | Kingdom of Tonga | 120 | 78.63(47.24-130.92) |  | Republic of Lithuania | 120 | 159 |  | Republic of Guatemala | 120 | 46.09(20.50-89.92) |
| Republic of Benin | 121 | 80 |  | Republic of Malta | 121 | 77.64(50.31-109.72) |  | Republic of Rwanda | 121 | 192 |  | Republic of Kiribati | 121 | 45.19(11.67-93.25) |
| Republic of Fiji | 122 | 133 |  | Federal Democratic Republic of Ethiopia | 122 | 76.63(32.32-133.13) |  | Republic of Paraguay | 122 | 100 |  | Arab Republic of Egypt | 122 | 44.07(17.12-74.97) |
| Republic of Latvia | 123 | 157 |  | Republic of Chile | 123 | 76.31(53.24-99.00) |  | Republic of Senegal | 123 | 144 |  | Republic of Mali | 123 | 43.05(8.19-94.98) |
| Republic of Liberia | 124 | 92 |  | Grand Duchy of Luxembourg | 124 | 74.23(46.58-102.84) |  | Kingdom of Norway | 124 | 181 |  | Republic of Chile | 124 | 42.71(24.04-61.09) |
| Republic of Trinidad and Tobago | 125 | 137 |  | Republic of South Africa | 125 | 72.63(49.97-99.21) |  | Swiss Confederation | 125 | 167 |  | United States Virgin Islands | 125 | 42.19(20.58-74.96) |
| Eastern Republic of Uruguay | 126 | 140 |  | Argentine Republic | 126 | 71.19(41.80-95.57) |  | Eastern Republic of Uruguay | 126 | 126 |  | State of Qatar | 126 | 41.90(17.07-74.51) |
| Republic of Singapore | 127 | 121 |  | Antigua and Barbuda | 127 | 70.35(47.85-94.23) |  | Republic of the Congo | 127 | 64 |  | Eastern Republic of Uruguay | 127 | 41.81(20.15-65.58) |
| Republic of Burundi | 128 | 117 |  | Republic of Trinidad and Tobago | 128 | 70.10(38.92-109.22) |  | Central African Republic | 128 | 74 |  | Portuguese Republic | 128 | 41.79(22.69-64.27) |
| Palestine | 129 | 104 |  | Republic of Fiji | 129 | 68.77(35.31-117.00) |  | Hellenic Republic | 129 | 97 |  | Palestine | 129 | 40.72(11.05-72.48) |
| Jamaica | 130 | 123 |  | Republic of Turkey | 130 | 66.31(37.78-96.56) |  | Republic of Belarus | 130 | 101 |  | Republic of San Marino | 130 | 39.98(22.61-62.18) |
| Republic of Albania | 131 | 156 |  | Australia | 131 | 66.27(35.18-96.25) |  | Bosnia and Herzegovina | 131 | 139 |  | Kingdom of Belgium | 131 | 38.71(16.48-62.80) |
| Sultanate of Oman | 132 | 88 |  | Republic of Azerbaijan | 132 | 64.53(37.77-91.54) |  | Canada | 132 | 149 |  | Republic of Cuba | 132 | 38.24(18.98-61.06) |
| Lao People's Democratic Republic | 133 | 114 |  | Republic of Rwanda | 133 | 64.33(10.68-135.66) |  | Republic of Ghana | 133 | 104 |  | Republic of Bulgaria | 133 | 37.80(15.42-68.29) |
| Central African Republic | 134 | 105 |  | United States of America | 134 | 63.92(41.95-103.37) |  | Islamic Republic of Iran | 134 | 66 |  | Barbados | 134 | 37.19(18.15-62.64) |
| Republic of Chad | 135 | 95 |  | Tuvalu | 135 | 62.55(30.79-113.46) |  | Malaysia | 135 | 116 |  | Bosnia and Herzegovina | 135 | 37.03(12.71-62.05) |
| North Macedonia | 136 | 153 |  | Saint Vincent and the Grenadines | 136 | 61.03(38.87-90.49) |  | Principality of Andorra | 136 | 160 |  | Japan | 136 | 36.02(12.67-60.16) |
| Mongolia | 137 | 126 |  | Republic of Guyana | 137 | 58.17(28.16-95.99) |  | Republic of Tunisia | 137 | 61 |  | Federated States of Micronesia | 137 | 35.77(5.63-82.30) |
| Republic of the Congo | 138 | 98 |  | Grenada | 138 | 57.34(32.43-83.51) |  | Federal Republic of Somalia | 138 | 171 |  | Republic of Albania | 138 | 35.27(15.90-57.47) |
| Islamic Republic of Mauritania | 139 | 127 |  | Republic of Palau | 139 | 54.72(22.15-102.96) |  | Republic of Mali | 139 | 150 |  | Canada | 139 | 34.72(14.33-62.49) |
| Republic of South Sudan | 140 | 131 |  | United Kingdom of Great Britain and Northern Ireland | 140 | 52.10(35.92-74.86) |  | Union of the Comoros | 140 | 107 |  | Republic of Italy | 140 | 34.56(15.65-51.30) |
| Togolese Republic | 141 | 106 |  | Eastern Republic of Uruguay | 141 | 49.36(26.54-74.39) |  | Kingdom of Belgium | 141 | 163 |  | Republic of Honduras | 141 | 33.62(4.32-66.68) |
| Republic of Sierra Leone | 142 | 108 |  | Republic of Nauru | 142 | 48.15(19.03-87.72) |  | Republic of San Marino | 142 | 162 |  | Republic of Peru | 142 | 32.78(4.74-67.17) |
| Republic of Slovenia | 143 | 158 |  | Federated States of Micronesia | 143 | 43.90(11.96-93.23) |  | Kingdom of Spain | 143 | 137 |  | Federative Republic of Brazil | 143 | 32.28(11.30-49.69) |
| Republic of Estonia | 144 | 161 |  | Canada | 144 | 43.75(21.99-73.38) |  | State of Israel | 144 | 185 |  | Principality of Andorra | 144 | 32.17(9.65-53.57) |
| Republic of Guyana | 145 | 152 |  | American Samoa | 145 | 42.24(12.93-80.76) |  | Republic of Zambia | 145 | 125 |  | Republic of Armenia | 145 | 31.79(11.72-51.59) |
| Republic of Panama | 146 | 122 |  | Republic of Iceland | 146 | 41.69(24.81-65.74) |  | Republic of Côte d'Ivoire | 146 | 99 |  | Republic of Senegal | 146 | 31.68(3.22-73.10) |
| Republic of Mauritius | 147 | 149 |  | Kingdom of Sweden | 147 | 40.61(20.95-60.30) |  | Republic of Cabo Verde | 147 | 113 |  | Republic of Ecuador | 147 | 31.26(7.24-59.72) |
| Gabonese Republic | 148 | 139 |  | Tokelau | 148 | 39.15(9.00-85.31) |  | Kingdom of Denmark | 148 | 176 |  | Republic of Kazakhstan | 148 | 31.14(7.23-56.97) |
| Kingdom of Bahrain | 149 | 132 |  | Syrian Arab Republic | 149 | 38.19(5.90-78.43) |  | Kingdom of Sweden | 149 | 178 |  | Kingdom of the Netherlands | 149 | 30.58(12.01-49.57) |
| State of Qatar | 150 | 96 |  | Ireland | 150 | 37.10(18.18-55.56) |  | Federal Democratic Republic of Ethiopia | 150 | 202 |  | Syrian Arab Republic | 150 | 30.34(-0.11-68.30) |
| State of Eritrea | 151 | 135 |  | French Republic | 151 | 36.40(11.41-60.11) |  | Republic of the Gambia | 151 | 105 |  | Australia | 151 | 29.89(5.60-53.30) |
| Republic of Namibia | 152 | 148 |  | Ukraine | 152 | 35.42(11.19-72.86) |  | Republic of Malta | 152 | 121 |  | Grenada | 152 | 29.79(9.24-51.37) |
| Republic of Guinea-Bissau | 153 | 144 |  | Principality of Andorra | 153 | 34.74(11.79-56.57) |  | Kingdom of the Netherlands | 153 | 179 |  | Federal Republic of Somalia | 153 | 29.38(-12.00-78.56) |
| Kingdom of Lesotho | 154 | 141 |  | Republic of Kazakhstan | 154 | 34.63(10.08-61.14) |  | Republic of Maldives | 154 | 165 |  | Russian Federation | 154 | 29.16(12.59-45.39) |
| Montenegro | 155 | 172 |  | Republic of the Union of Myanmar | 155 | 34.59(-2.08-86.85) |  | Republic of Yemen | 155 | 123 |  | Republic of Nauru | 155 | 28.70(3.40-63.08) |
| Kingdom of Eswatini | 156 | 151 |  | Guam | 156 | 33.62(19.65-50.94) |  | Kingdom of Bhutan | 156 | 147 |  | Romania | 156 | 28.62(4.54-59.97) |
| Republic of Botswana | 157 | 145 |  | Republic of San Marino | 157 | 33.58(17.00-54.77) |  | Republic of Benin | 157 | 146 |  | Kingdom of Denmark | 157 | 27.73(7.35-49.62) |
| Republic of the Gambia | 158 | 143 |  | Republic of Korea | 158 | 32.41(0.50-71.03) |  | Republic of Albania | 158 | 180 |  | Antigua and Barbuda | 158 | 27.71(10.84-45.62) |
| Solomon Islands | 159 | 154 |  | Kingdom of Belgium | 159 | 30.58(9.65-53.25) |  | Republic of Cyprus | 159 | 132 |  | Kingdom of Sweden | 159 | 27.34(9.53-45.16) |
| Commonwealth of the Bahamas | 160 | 164 |  | New Zealand | 160 | 29.11(12.84-46.87) |  | Democratic Socialist Republic of Sri Lanka | 160 | 152 |  | Kyrgyz Republic | 160 | 26.16(3.83-53.79) |
| Republic of Cyprus | 161 | 159 |  | Kingdom of Spain | 161 | 28.67(8.98-48.18) |  | Republic of Namibia | 161 | 141 |  | Bermuda | 161 | 25.44(6.14-45.29) |
| Republic of Suriname | 162 | 160 |  | Principality of Monaco | 162 | 27.49(9.55-52.64) |  | Republic of Guinea | 162 | 158 |  | Republic of Iraq | 162 | 25.30(1.37-61.33) |
| Kingdom of Bhutan | 163 | 167 |  | Swiss Confederation | 163 | 27.06(5.93-52.36) |  | Republic of Singapore | 163 | 122 |  | Principality of Monaco | 163 | 25.13(7.52-49.82) |
| Independent State of Samoa | 164 | 168 |  | Saint Kitts and Nevis | 164 | 24.35(2.69-51.29) |  | Democratic Republic of Sao Tome and Principe | 164 | 111 |  | Republic of Turkey | 164 | 24.86(3.44-47.57) |
| Republic of Equatorial Guinea | 165 | 147 |  | Barbados | 165 | 24.15(6.93-47.19) |  | Republic of Panama | 165 | 96 |  | Republic of Serbia | 165 | 24.80(-0.14-50.72) |
| Federated States of Micronesia | 166 | 175 |  | Hellenic Republic | 166 | 23.06(7.26-40.45) |  | Republic of Austria | 166 | 191 |  | Montenegro | 166 | 24.27(3.91-43.61) |
| Barbados | 167 | 177 |  | Republic of Belarus | 167 | 22.98(-0.75-49.40) |  | Islamic Republic of Pakistan | 167 | 86 |  | Republic of Azerbaijan | 167 | 23.44(3.37-43.71) |
| Republic of Kiribati | 168 | 169 |  | Kingdom of Denmark | 168 | 22.09(2.61-43.01) |  | Togolese Republic | 168 | 153 |  | Puerto Rico | 168 | 23.26(9.25-41.39) |
| Brunei Darussalam | 169 | 162 |  | Republic of Moldova | 169 | 21.06(4.18-45.07) |  | Lao People's Democratic Republic | 169 | 173 |  | Republic of Finland | 169 | 23.16(4.75-43.17) |
| Grand Duchy of Luxembourg | 170 | 176 |  | Republic of Niue | 170 | 18.71(-9.81-60.02) |  | Republic of Angola | 170 | 169 |  | Republic of Iceland | 170 | 23.00(8.34-43.87) |
| Union of the Comoros | 171 | 166 |  | Kingdom of Norway | 171 | 14.50(1.02-26.68) |  | Republic of Malawi | 171 | 177 |  | Republic of Lithuania | 171 | 21.89(1.76-45.16) |
| Guam | 172 | 179 |  | Kingdom of the Netherlands | 172 | 14.35(-1.91-30.98) |  | French Republic | 172 | 184 |  | Swiss Confederation | 172 | 20.58(0.53-44.59) |
| Republic of Vanuatu | 173 | 163 |  | North Macedonia | 173 | 13.63(-5.55-34.57) |  | Republic of Madagascar | 173 | 183 |  | Ireland | 173 | 20.19(3.60-36.38) |
| Republic of Iceland | 174 | 182 |  | Republic of Finland | 174 | 13.02(-3.88-31.39) |  | Kingdom of Lesotho | 174 | 77 |  | Republic of Tajikistan | 174 | 19.94(-2.60-45.03) |
| Belize | 175 | 165 |  | Republic of Austria | 175 | 12.18(-4.80-30.12) |  | Republic of Italy | 175 | 189 |  | Argentine Republic | 175 | 19.34(-1.15-36.33) |
| Republic of Malta | 176 | 180 |  | Portuguese Republic | 176 | 10.54(-4.35-28.07) |  | Republic of the Niger | 176 | 198 |  | Republic of Austria | 176 | 19.31(1.25-38.39) |
| Republic of Cabo Verde | 177 | 171 |  | Russian Federation | 177 | 3.19(-10.04-16.16) |  | Democratic Republic of the Congo | 177 | 164 |  | Republic of Latvia | 177 | 18.65(2.37-39.80) |
| Republic of the Marshall Islands | 178 | 178 |  | Republic of Serbia | 178 | 3.01(-17.57-24.42) |  | Republic of Korea | 178 | 175 |  | Tuvalu | 178 | 18.06(-5.01-55.04) |
| United States Virgin Islands | 179 | 189 |  | Montenegro | 179 | 1.84(-14.84-17.69) |  | Republic of Indonesia | 179 | 145 |  | North Macedonia | 179 | 17.99(-1.93-39.73) |
| Saint Lucia | 180 | 181 |  | Republic of Cuba | 180 | 1.58(-12.57-18.35) |  | Republic of Botswana | 180 | 131 |  | Cook Islands | 180 | 17.23(-10.11-54.78) |
| Democratic Republic of Timor-Leste | 181 | 174 |  | Republic of Italy | 181 | -0.44(-14.44-11.94) |  | United Republic of Tanzania | 181 | 170 |  | Grand Duchy of Luxembourg | 181 | 16.55(-1.95-35.69) |
| Kingdom of Tonga | 182 | 183 |  | Republic of Armenia | 182 | -1.42(-16.43-13.39) |  | Federal Republic of Nigeria | 182 | 186 |  | Plurinational State of Bolivia | 182 | 15.45(-13.32-49.82) |
| Northern Mariana Islands | 183 | 192 |  | Japan | 183 | -1.63(-18.52-15.83) |  | Federal Democratic Republic of Nepal | 183 | 161 |  | Republic of Burundi | 183 | 14.97(-19.23-61.66) |
| American Samoa | 184 | 185 |  | Federal Republic of Germany | 184 | -8.92(-23.18-5.26) |  | Taiwan (Province of China) | 184 | 140 |  | Georgia | 184 | 13.99(-1.12-35.47) |
| Republic of Djibouti | 185 | 170 |  | Republic of Albania | 185 | -9.72(-22.64-5.10) |  | Republic of Sierra Leone | 185 | 172 |  | Republic of Slovenia | 185 | 13.30(-7.76-31.16) |
| Grenada | 186 | 187 |  | Puerto Rico | 186 | -9.88(-20.12-3.38) |  | Japan | 186 | 196 |  | Islamic Republic of Mauritania | 186 | 12.48(-12.35-46.43) |
| Saint Vincent and the Grenadines | 187 | 186 |  | Cook Islands | 187 | -10.50(-31.37-18.17) |  | Republic of Burundi | 187 | 200 |  | Republic of South Africa | 187 | 12.03(-2.67-29.29) |
| Republic of Maldives | 188 | 173 |  | Republic of Bulgaria | 188 | -12.07(-26.35-7.39) |  | Kingdom of Thailand | 188 | 115 |  | Republic of Croatia | 188 | 9.79(-10.51-31.44) |
| Greenland | 189 | 197 |  | Slovak Republic | 189 | -14.17(-30.74-3.24) |  | Republic of Mozambique | 189 | 174 |  | State of Israel | 189 | 8.54(-7.86-22.99) |
| Republic of Seychelles | 190 | 188 |  | Bermuda | 190 | -14.59(-27.73-1.08) |  | Burkina Faso | 190 | 195 |  | Northern Mariana Islands | 190 | 8.29(-15.15-49.70) |
| Commonwealth of Dominica | 191 | 190 |  | Republic of Slovenia | 191 | -15.82(-31.46-2.54) |  | People's Republic of China | 191 | 124 |  | Federal Republic of Germany | 191 | 6.96(-9.79-23.61) |
| Antigua and Barbuda | 192 | 191 |  | United States Virgin Islands | 192 | -16.98(-29.60-2.15) |  | Republic of Kenya | 192 | 182 |  | Republic of the Niger | 192 | 6.02(-22.34-44.63) |
| Saint Kitts and Nevis | 193 | 194 |  | Romania | 193 | -20.21(-35.15-0.76) |  | Republic of Chad | 193 | 194 |  | Kingdom of Norway | 193 | 3.21(-8.94-14.20) |
| Bermuda | 194 | 200 |  | Czech Republic | 194 | -21.59(-34.52-9.70) |  | Republic of India | 194 | 187 |  | Republic of the Union of Myanmar | 194 | 2.78(-25.23-42.68) |
| Cook Islands | 195 | 196 |  | Northern Mariana Islands | 195 | -23.76(-40.27-5.39) |  | Republic of Uganda | 195 | 190 |  | Slovak Republic | 195 | 2.67(-17.15-23.49) |
| Republic of Nauru | 196 | 193 |  | Republic of Croatia | 196 | -24.48(-38.44-9.58) |  | Democratic People's Republic of Korea | 196 | 188 |  | New Zealand | 196 | -0.98(-13.47-12.63) |
| Democratic Republic of Sao Tome and Principe | 197 | 184 |  | Republic of Poland | 197 | -27.34(-40.72-14.93) |  | Kingdom of Cambodia | 197 | 199 |  | Czech Republic | 197 | -1.48(-17.73-13.46) |
| Republic of Palau | 198 | 195 |  | Bosnia and Herzegovina | 198 | -27.39(-40.28-14.13) |  | People's Republic of Bangladesh | 198 | 168 |  | Republic of Estonia | 198 | -5.06(-22.23-10.91) |
| Principality of Andorra | 199 | 199 |  | Republic of Lithuania | 199 | -29.57(-41.20-16.12) |  | Republic of South Sudan | 199 | 201 |  | Saint Kitts and Nevis | 199 | -5.44(-21.92-15.04) |
| Tuvalu | 200 | 198 |  | Republic of Latvia | 200 | -33.01(-42.20-21.07) |  | Republic of Djibouti | 200 | 193 |  | Republic of Poland | 200 | -13.24(-29.23-1.57) |
| Principality of Monaco | 201 | 201 |  | Republic of Estonia | 201 | -33.90(-45.85-22.78) |  | Republic of Zimbabwe | 201 | 127 |  | Greenland | 201 | -17.00(-31.07--1.40) |
| Republic of San Marino | 202 | 202 |  | Greenland | 202 | -36.04(-46.88-24.01) |  | State of Eritrea | 202 | 197 |  | Republic of Rwanda | 202 | -20.59(-46.52-13.88) |
| Republic of Niue | 203 | 203 |  | Georgia | 203 | -39.25(-47.30-27.80) |  | Democratic Republic of Timor-Leste | 203 | 203 |  | Hungary | 203 | -22.64(-37.32--9.24) |
| Tokelau | 204 | 204 |  | Hungary | 204 | -42.41(-53.34-32.43) |  | Socialist Republic of Viet Nam | 204 | 204 |  | Federal Democratic Republic of Ethiopia | 204 | -30.38(-47.85--8.12) |

DALYs- disability-adjusted life years; DALY- disability-adjusted life year; UI- uncertainty interval.

**Table S5.** EAPC ranks of High BMI-related death and DALY rates in the youth and young adults of 204 countries/territories- 1990-2021

| **EAPC of death rate(95%CI)** | | |  | **EAPC of DALY rate(95%CI)** | | |
| --- | --- | --- | --- | --- | --- | --- |
| **(Descending)** | | |  | **(Descending)** | | |
| **Country or region** | **Rank** | **Value (95%CI)** |  | **Country or region** | **Rank** | **Value (95%CI)** |
|  | **1990-2021** |  |  |  | **1990-2021** |  |
| Zimbabwe | 1 | 6.66(5.53-7.80) |  | Republic of Zimbabwe | 1 | 5.17(4.47-5.87) |
| Lesotho | 2 | 6.07(5.33-6.80) |  | Kingdom of Lesotho | 2 | 4.75(4.28-5.23) |
| Libya | 3 | 3.89(3.56-4.22) |  | People's Republic of Bangladesh | 3 | 4.23(3.94-4.53) |
| Eswatini | 4 | 3.72(2.87-4.57) |  | State of Libya | 4 | 3.96(3.73-4.19) |
| Mauritius | 5 | 3.26(2.92-3.59) |  | People's Republic of China | 5 | 3.41(3.19-3.64) |
| Saudi Arabia | 6 | 3.20(2.94-3.47) |  | Kingdom of Saudi Arabia | 6 | 3.20(3.02-3.39) |
| Mozambique | 7 | 3.15(2.89-3.42) |  | Republic of Mauritius | 7 | 3.20(2.95-3.44) |
| Bangladesh | 8 | 3.07(2.68-3.46) |  | Socialist Republic of Viet Nam | 8 | 3.19(2.78-3.61) |
| Pakistan | 9 | 2.90(2.62-3.18) |  | Islamic Republic of Pakistan | 9 | 3.18(2.98-3.37) |
| Viet Nam | 10 | 2.83(2.32-3.35) |  | Kingdom of Eswatini | 10 | 3.15(2.55-3.76) |
| Kenya | 11 | 2.81(2.50-3.12) |  | Republic of Mozambique | 11 | 3.10(2.89-3.30) |
| Dominican Republic | 12 | 2.78(2.46-3.10) |  | Republic of Botswana | 12 | 3.00(2.69-3.31) |
| Panama | 13 | 2.59(2.39-2.79) |  | Dominican Republic | 13 | 2.90(2.71-3.09) |
| Indonesia | 14 | 2.50(2.15-2.86) |  | Republic of Tunisia | 14 | 2.88(2.79-2.97) |
| Botswana | 15 | 2.44(1.96-2.92) |  | Islamic Republic of Iran | 15 | 2.80(2.61-2.99) |
| Sierra Leone | 16 | 2.37(2.28-2.45) |  | Republic of Panama | 16 | 2.77(2.68-2.86) |
| Solomon Islands | 17 | 2.36(2.21-2.51) |  | Taiwan (Province of China) | 17 | 2.68(2.62-2.74) |
| Thailand | 18 | 2.26(1.30-3.23) |  | Republic of Kenya | 18 | 2.67(2.47-2.87) |
| Dominica | 19 | 2.23(2.09-2.37) |  | Kingdom of Thailand | 19 | 2.60(1.89-3.30) |
| China | 20 | 2.22(1.99-2.45) |  | Republic of the Congo | 20 | 2.54(2.33-2.75) |
| Côte d'Ivoire | 21 | 2.19(1.99-2.40) |  | Federal Democratic Republic of Nepal | 21 | 2.48(2.24-2.72) |
| Congo | 22 | 2.05(1.77-2.32) |  | Republic of Indonesia | 22 | 2.46(2.21-2.72) |
| Eritrea | 23 | 1.97(1.86-2.08) |  | State of Eritrea | 23 | 2.44(2.34-2.54) |
| Togo | 24 | 1.96(1.82-2.11) |  | Solomon Islands | 24 | 2.41(2.28-2.54) |
| Iran (Islamic Republic of) | 25 | 1.94(1.72-2.16) |  | Republic of Sierra Leone | 25 | 2.41(2.34-2.47) |
| Palau | 26 | 1.93(1.76-2.09) |  | Sultanate of Oman | 26 | 2.40(2.16-2.65) |
| Central African Republic | 27 | 1.90(1.79-2.01) |  | Republic of Côte d'Ivoire | 27 | 2.33(2.19-2.47) |
| Liberia | 28 | 1.89(1.53-2.26) |  | Democratic Republic of Sao Tome and Principe | 28 | 2.27(2.01-2.53) |
| Tunisia | 29 | 1.85(1.76-1.95) |  | Central African Republic | 29 | 2.27(2.18-2.35) |
| Djibouti | 30 | 1.84(1.58-2.10) |  | People's Democratic Republic of Algeria | 30 | 2.24(2.06-2.41) |
| Philippines | 31 | 1.82(1.69-1.95) |  | Republic of India | 31 | 2.23(2.06-2.40) |
| Gambia | 32 | 1.78(1.49-2.08) |  | United Republic of Tanzania | 32 | 2.23(2.14-2.31) |
| Mexico | 33 | 1.78(1.40-2.16) |  | Togolese Republic | 33 | 2.18(2.10-2.26) |
| United Republic of Tanzania | 34 | 1.78(1.65-1.90) |  | Republic of Liberia | 34 | 2.15(1.86-2.44) |
| Oman | 35 | 1.64(1.37-1.91) |  | Republic of Djibouti | 35 | 2.08(1.91-2.25) |
| Nicaragua | 36 | 1.63(1.39-1.88) |  | Republic of Suriname | 36 | 2.08(1.90-2.26) |
| India | 37 | 1.62(1.31-1.94) |  | Commonwealth of Dominica | 37 | 2.05(1.96-2.15) |
| Samoa | 38 | 1.61(1.50-1.72) |  | Republic of Singapore | 38 | 2.05(1.77-2.33) |
| Yemen | 39 | 1.60(1.39-1.83) |  | Republic of Yemen | 39 | 2.04(1.75-2.33) |
| Tonga | 40 | 1.60(1.40-1.79) |  | United Arab Emirates | 40 | 2.02(1.78-2.26) |
| Sao Tome and Principe | 41 | 1.57(1.12-2.03) |  | Republic of Malta | 41 | 2.01(1.91-2.12) |
| Vanuatu | 42 | 1.57(1.45-1.68) |  | Republic of Palau | 42 | 1.98(1.85-2.12) |
| Guinea | 43 | 1.55(1.46-1.64) |  | Republic of the Gambia | 43 | 1.98(1.78-2.18) |
| Taiwan | 44 | 1.54(1.39-1.69) |  | Republic of Angola | 44 | 1.96(1.84-2.08) |
| Marshall Islands | 45 | 1.52(1.36-1.68) |  | Democratic Republic of the Congo | 45 | 1.92(1.81-2.04) |
| El Salvador | 46 | 1.52(1.24-1.80) |  | Independent State of Samoa | 46 | 1.88(1.79-1.98) |
| Democratic Republic of the Congo | 47 | 1.47(1.35-1.60) |  | Democratic People's Republic of Korea | 47 | 1.87(1.71-2.02) |
| Democratic People's Republic of Korea | 48 | 1.45(1.24-1.66) |  | Republic of the Marshall Islands | 48 | 1.86(1.74-1.99) |
| Suriname | 49 | 1.41(1.09-1.73) |  | Republic of Vanuatu | 49 | 1.85(1.77-1.93) |
| Nepal | 50 | 1.40(1.17-1.63) |  | Republic of Haiti | 50 | 1.84(1.65-2.04) |
| Cameroon | 51 | 1.38(1.06-1.69) |  | Democratic Republic of Timor-Leste | 51 | 1.82(1.42-2.22) |
| Guam | 52 | 1.36(1.03-1.68) |  | United Mexican States | 52 | 1.82(1.69-1.94) |
| Haiti | 53 | 1.35(1.10-1.59) |  | Republic of Nicaragua | 53 | 1.78(1.64-1.92) |
| Timor-Leste | 54 | 1.32(0.72-1.93) |  | Kingdom of Tonga | 54 | 1.78(1.63-1.92) |
| South Sudan | 55 | 1.29(0.85-1.73) |  | Hellenic Republic | 55 | 1.77(1.55-1.98) |
| Angola | 56 | 1.28(1.11-1.45) |  | Republic of Cabo Verde | 56 | 1.76(1.63-1.89) |
| Chad | 57 | 1.28(1.02-1.54) |  | Republic of the Philippines | 57 | 1.75(1.65-1.85) |
| Guinea-Bissau | 58 | 1.27(1.22-1.32) |  | Republic of Guinea | 58 | 1.74(1.61-1.87) |
| Belize | 59 | 1.22(0.74-1.71) |  | Islamic Republic of Afghanistan | 59 | 1.73(1.24-2.23) |
| Ukraine | 60 | 1.18(0.71-1.66) |  | Republic of Cameroon | 60 | 1.72(1.49-1.95) |
| American Samoa | 61 | 1.16(0.98-1.35) |  | Brunei Darussalam | 61 | 1.67(1.43-1.90) |
| Venezuela (Bolivarian Republic of) | 62 | 1.15(0.62-1.69) |  | Republic of Ghana | 62 | 1.63(1.52-1.74) |
| Malawi | 63 | 1.15(0.88-1.41) |  | Kingdom of Morocco | 63 | 1.63(1.54-1.72) |
| Uzbekistan | 64 | 1.14(0.88-1.40) |  | Republic of El Salvador | 64 | 1.62(1.47-1.76) |
| Uganda | 65 | 1.13(0.68-1.58) |  | Republic of Uzbekistan | 65 | 1.61(1.45-1.78) |
| Guyana | 66 | 1.13(0.70-1.55) |  | Republic of Guyana | 66 | 1.61(1.29-1.93) |
| Costa Rica | 67 | 1.12(0.60-1.63) |  | Republic of Cyprus | 67 | 1.61(1.45-1.77) |
| Madagascar | 68 | 1.09(0.97-1.22) |  | Republic of Paraguay | 68 | 1.61(1.54-1.68) |
| Lao People's Democratic Republic | 69 | 1.06(0.98-1.15) |  | Republic of Costa Rica | 69 | 1.59(1.39-1.79) |
| Jamaica | 70 | 1.04(0.40-1.68) |  | Republic of Guinea-Bissau | 70 | 1.58(1.51-1.64) |
| Paraguay | 71 | 1.03(0.90-1.17) |  | Democratic Socialist Republic of Sri Lanka | 71 | 1.57(1.40-1.74) |
| Ghana | 72 | 1.03(0.85-1.21) |  | Republic of Benin | 72 | 1.56(1.49-1.63) |
| Namibia | 73 | 0.99(0.40-1.59) |  | State of Kuwait | 73 | 1.54(1.36-1.73) |
| Gabon | 74 | 0.98(0.63-1.32) |  | Republic of Chad | 74 | 1.54(1.36-1.72) |
| Fiji | 75 | 0.97(0.83-1.11) |  | Bolivarian Republic of Venezuela | 75 | 1.53(1.21-1.86) |
| Afghanistan | 76 | 0.97(0.34-1.60) |  | French Republic | 76 | 1.53(1.43-1.63) |
| Trinidad and Tobago | 77 | 0.93(0.58-1.29) |  | Gabonese Republic | 77 | 1.53(1.27-1.78) |
| Benin | 78 | 0.92(0.75-1.09) |  | American Samoa | 78 | 1.52(1.35-1.70) |
| Papua New Guinea | 79 | 0.90(0.76-1.03) |  | Republic of Korea | 79 | 1.52(1.30-1.73) |
| Egypt | 80 | 0.87(0.47-1.28) |  | Lao People's Democratic Republic | 80 | 1.51(1.44-1.58) |
| Somalia | 81 | 0.85(0.41-1.28) |  | Saint Vincent and the Grenadines | 81 | 1.51(1.34-1.68) |
| Republic of Moldova | 82 | 0.85(0.51-1.19) |  | Republic of Uganda | 82 | 1.51(1.20-1.82) |
| Burkina Faso | 83 | 0.84(0.66-1.02) |  | Republic of Seychelles | 83 | 1.50(1.27-1.74) |
| Algeria | 84 | 0.82(0.56-1.09) |  | Belize | 84 | 1.49(1.19-1.80) |
| Tokelau | 85 | 0.82(0.58-1.06) |  | Lebanese Republic | 85 | 1.49(1.14-1.84) |
| United States Virgin Islands | 86 | 0.81(0.53-1.09) |  | Kingdom of Spain | 86 | 1.48(1.43-1.54) |
| United States of America | 87 | 0.80(0.64-0.96) |  | Republic of Moldova | 87 | 1.48(1.27-1.69) |
| Sudan | 88 | 0.78(0.72-0.84) |  | Jamaica | 88 | 1.46(1.10-1.83) |
| Micronesia (Federated States of) | 89 | 0.76(0.72-0.80) |  | Arab Republic of Egypt | 89 | 1.46(1.14-1.78) |
| Nigeria | 90 | 0.73(0.48-0.98) |  | Republic of Trinidad and Tobago | 90 | 1.45(1.22-1.69) |
| Mongolia | 91 | 0.71(0.24-1.20) |  | Union of the Comoros | 91 | 1.45(1.07-1.82) |
| Saint Vincent and the Grenadines | 92 | 0.70(0.46-0.94) |  | Republic of Sudan | 92 | 1.43(1.40-1.46) |
| Bulgaria | 93 | 0.70(0.54-0.85) |  | Republic of Namibia | 93 | 1.41(1.00-1.83) |
| Malaysia | 94 | 0.68(0.39-0.98) |  | Independent State of Papua New Guinea | 94 | 1.41(1.32-1.50) |
| Kiribati | 95 | 0.65(0.45-0.86) |  | Ukraine | 95 | 1.41(1.18-1.64) |
| Comoros | 96 | 0.65(0.06-1.24) |  | Republic of South Sudan | 96 | 1.37(1.06-1.68) |
| Seychelles | 97 | 0.60(0.28-0.93) |  | Saint Lucia | 97 | 1.37(1.27-1.47) |
| Cambodia | 98 | 0.58(0.30-0.86) |  | Republic of Mali | 98 | 1.36(1.27-1.45) |
| Greece | 99 | 0.57(0.06-1.08) |  | United States of America | 99 | 1.33(1.27-1.40) |
| Niue | 100 | 0.57(0.33-0.80) |  | Republic of Equatorial Guinea | 100 | 1.32(1.01-1.63) |
| Nauru | 101 | 0.54(0.17-0.90) |  | Tokelau | 101 | 1.32(1.15-1.48) |
| Zambia | 102 | 0.53(0.25-0.82) |  | Kingdom of Bhutan | 102 | 1.31(1.20-1.43) |
| Cabo Verde | 103 | 0.53(0.26-0.79) |  | Republic of Niue | 103 | 1.31(1.15-1.46) |
| United Arab Emirates | 104 | 0.49(0.20-0.79) |  | Burkina Faso | 104 | 1.31(1.16-1.45) |
| Saint Lucia | 105 | 0.49(0.28-0.69) |  | Republic of Malawi | 105 | 1.30(1.11-1.50) |
| Senegal | 106 | 0.48(0.29-0.66) |  | Malaysia | 106 | 1.30(1.14-1.46) |
| Mali | 107 | 0.47(0.33-0.61) |  | Mongolia | 107 | 1.30(1.00-1.60) |
| Guatemala | 108 | 0.46(-0.04-0.96) |  | Republic of Fiji | 108 | 1.28(1.17-1.40) |
| Syrian Arab Republic | 109 | 0.43(0.04-0.82) |  | Republic of Zambia | 109 | 1.26(1.05-1.47) |
| Equatorial Guinea | 110 | 0.42(0.01-0.82) |  | Guam | 110 | 1.26(1.05-1.47) |
| Turkmenistan | 111 | 0.41(-0.20-1.02) |  | Federal Republic of Nigeria | 111 | 1.26(1.07-1.44) |
| Sri Lanka | 112 | 0.36(0.06-0.67) |  | Republic of Madagascar | 112 | 1.23(1.11-1.36) |
| Bahamas | 113 | 0.34(0.16-0.52) |  | Syrian Arab Republic | 113 | 1.23(0.96-1.49) |
| Antigua and Barbuda | 114 | 0.19(-0.27-0.64) |  | Federal Republic of Somalia | 114 | 1.22(0.85-1.59) |
| Bhutan | 115 | 0.16(0.01-0.31) |  | Eastern Republic of Uruguay | 115 | 1.22(1.10-1.34) |
| Peru | 116 | 0.16(-0.26-0.58) |  | United States Virgin Islands | 116 | 1.21(1.06-1.37) |
| Brunei Darussalam | 117 | 0.16(-0.19-0.51) |  | Kingdom of Cambodia | 117 | 1.21(0.93-1.48) |
| Tuvalu | 118 | 0.14(0.07-0.22) |  | Japan | 118 | 1.17(1.06-1.29) |
| Morocco | 119 | 0.10(-0.13-0.33) |  | Republic of Colombia | 119 | 1.17(0.98-1.35) |
| Kuwait | 120 | 0.05(-0.35-0.45) |  | Republic of San Marino | 120 | 1.16(0.95-1.37) |
| Grenada | 121 | -0.05(-0.43-0.33) |  | Republic of Guatemala | 121 | 1.16(0.82-1.50) |
| Bahrain | 122 | -0.05(-0.49-0.39) |  | Antigua and Barbuda | 122 | 1.14(0.94-1.34) |
| Malta | 123 | -0.05(-0.38-0.28) |  | Portuguese Republic | 123 | 1.13(1.02-1.24) |
| Ecuador | 124 | -0.08(-0.43-0.27) |  | Federated States of Micronesia | 124 | 1.10(1.04-1.16) |
| Belarus | 125 | -0.08(-0.65-0.49) |  | Kingdom of Bahrain | 125 | 1.10(0.90-1.31) |
| Uruguay | 126 | -0.10(-0.37-0.18) |  | Republic of Maldives | 126 | 1.08(0.65-1.52) |
| Palestine | 127 | -0.15(-0.43-0.13) |  | Republic of Bulgaria | 127 | 1.07(0.97-1.17) |
| Cook Islands | 128 | -0.22(-0.44-0.01) |  | Kingdom of Belgium | 128 | 1.02(0.93-1.10) |
| Chile | 129 | -0.24(-0.32-0.17) |  | Republic of Chile | 129 | 1.01(0.95-1.07) |
| Colombia | 130 | -0.31(-0.67-0.06) |  | Republic of Senegal | 130 | 1.01(0.89-1.13) |
| Mauritania | 131 | -0.31(-0.42-0.20) |  | Hashemite Kingdom of Jordan | 131 | 1.00(0.79-1.20) |
| Brazil | 132 | -0.35(-0.57-0.12) |  | Republic of Italy | 132 | 0.99(0.77-1.21) |
| Barbados | 133 | -0.35(-0.63-0.07) |  | United Kingdom of Great Britain and Northern Ireland | 133 | 0.98(0.89-1.08) |
| Maldives | 134 | -0.40(-0.93-0.14) |  | Principality of Andorra | 134 | 0.96(0.92-1.00) |
| Albania | 135 | -0.45(-0.84-0.05) |  | Palestine | 135 | 0.95(0.79-1.12) |
| Monaco | 136 | -0.48(-0.84-0.12) |  | Republic of Peru | 136 | 0.94(0.66-1.22) |
| Canada | 137 | -0.48(-0.83-0.13) |  | Commonwealth of the Bahamas | 137 | 0.93(0.80-1.07) |
| Romania | 138 | -0.56(-0.77-0.34) |  | Romania | 138 | 0.89(0.75-1.03) |
| Puerto Rico | 139 | -0.57(-0.96-0.18) |  | Republic of Kiribati | 139 | 0.86(0.69-1.03) |
| Russian Federation | 140 | -0.60(-1.17-0.03) |  | Federative Republic of Brazil | 140 | 0.84(0.69-0.98) |
| Burundi | 141 | -0.61(-1.08-0.14) |  | Grenada | 141 | 0.82(0.59-1.06) |
| Andorra | 142 | -0.63(-0.86-0.40) |  | Republic of Ecuador | 142 | 0.80(0.61-1.00) |
| Northern Mariana Islands | 143 | -0.67(-0.96-0.37) |  | Argentine Republic | 143 | 0.78(0.59-0.97) |
| Iraq | 144 | -0.68(-1.03-0.33) |  | Kingdom of Sweden | 144 | 0.77(0.57-0.96) |
| San Marino | 145 | -0.75(-1.11-0.39) |  | Turkmenistan | 145 | 0.76(0.34-1.19) |
| Montenegro | 146 | -0.77(-1.10-0.44) |  | Canada | 146 | 0.76(0.64-0.88) |
| South Africa | 147 | -0.79(-2.01-0.45) |  | Republic of Iraq | 147 | 0.75(0.55-0.96) |
| Lebanon | 148 | -0.80(-1.39-0.21) |  | Republic of Finland | 148 | 0.74(0.64-0.84) |
| France | 149 | -0.82(-0.92-0.71) |  | Republic of Belarus | 149 | 0.74(0.46-1.02) |
| Niger | 150 | -0.86(-1.04-0.68) |  | Republic of Serbia | 150 | 0.73(0.67-0.80) |
| Argentina | 151 | -0.87(-1.19-0.54) |  | Republic of Iceland | 151 | 0.72(0.68-0.75) |
| Australia | 152 | -0.87(-1.15-0.59) |  | Republic of Nauru | 152 | 0.71(0.39-1.02) |
| Cuba | 153 | -0.89(-1.15-0.62) |  | Ireland | 153 | 0.70(0.55-0.86) |
| United Kingdom | 154 | -0.90(-1.08-0.72) |  | Cook Islands | 154 | 0.69(0.60-0.78) |
| Bolivia (Plurinational State of) | 155 | -0.91(-1.27-0.54) |  | Barbados | 155 | 0.66(0.49-0.84) |
| Myanmar | 156 | -0.96(-1.26-0.65) |  | Republic of Honduras | 156 | 0.66(0.47-0.84) |
| Jordan | 157 | -0.99(-1.35-0.63) |  | Republic of Turkey | 157 | 0.64(0.44-0.83) |
| Honduras | 158 | -1.04(-1.44-0.63) |  | Montenegro | 158 | 0.63(0.48-0.79) |
| North Macedonia | 159 | -1.07(-1.33-0.82) |  | Bermuda | 159 | 0.62(0.52-0.71) |
| Japan | 160 | -1.10(-1.30-0.91) |  | Czech Republic | 160 | 0.58(0.29-0.86) |
| Iceland | 161 | -1.13(-1.55-0.71) |  | Bosnia and Herzegovina | 161 | 0.57(0.34-0.80) |
| Bosnia and Herzegovina | 162 | -1.16(-1.55-0.76) |  | Kingdom of Denmark | 162 | 0.55(0.27-0.84) |
| Lithuania | 163 | -1.18(-1.68-0.67) |  | Kingdom of the Netherlands | 163 | 0.55(0.39-0.72) |
| Serbia | 164 | -1.21(-1.32-1.10) |  | Australia | 164 | 0.55(0.30-0.80) |
| Cyprus | 165 | -1.23(-1.84-0.62) |  | State of Qatar | 165 | 0.54(0.29-0.79) |
| Italy | 166 | -1.27(-1.53-1.02) |  | Republic of Cuba | 166 | 0.52(0.32-0.72) |
| Singapore | 167 | -1.28(-1.55-1.00) |  | Principality of Monaco | 167 | 0.50(0.33-0.67) |
| Bermuda | 168 | -1.33(-1.59-1.06) |  | Republic of Slovenia | 168 | 0.49(0.37-0.62) |
| Czechia | 169 | -1.33(-1.85-0.81) |  | Puerto Rico | 169 | 0.48(0.32-0.63) |
| Türkiye | 170 | -1.33(-1.62-1.05) |  | Tuvalu | 170 | 0.45(0.38-0.53) |
| Slovakia | 171 | -1.39(-1.70-1.07) |  | Swiss Confederation | 171 | 0.44(0.33-0.55) |
| Armenia | 172 | -1.42(-1.85-0.99) |  | Slovak Republic | 172 | 0.43(0.18-0.68) |
| Qatar | 173 | -1.54(-1.99-1.10) |  | North Macedonia | 173 | 0.43(0.32-0.54) |
| Sweden | 174 | -1.69(-1.86-1.53) |  | Republic of Albania | 174 | 0.43(0.20-0.65) |
| Ireland | 175 | -1.75(-2.11-1.39) |  | Republic of Croatia | 175 | 0.35(0.25-0.45) |
| New Zealand | 176 | -1.77(-2.03-1.50) |  | Republic of Austria | 176 | 0.31(0.20-0.41) |
| Kyrgyzstan | 177 | -1.80(-2.40-1.19) |  | State of Israel | 177 | 0.30(0.24-0.36) |
| Tajikistan | 178 | -1.80(-2.39-1.21) |  | Republic of Lithuania | 178 | 0.28(0.04-0.52) |
| Croatia | 179 | -1.84(-2.03-1.65) |  | Federal Republic of Germany | 179 | 0.27(0.18-0.36) |
| Spain | 180 | -1.91(-2.12-1.70) |  | Russian Federation | 180 | 0.23(-0.07-0.53) |
| Georgia | 181 | -2.06(-2.83-1.29) |  | Islamic Republic of Mauritania | 181 | 0.22(0.15-0.30) |
| Azerbaijan | 182 | -2.12(-2.57-1.67) |  | Plurinational State of Bolivia | 182 | 0.20(-0.04-0.45) |
| Belgium | 183 | -2.13(-2.30-1.95) |  | Grand Duchy of Luxembourg | 183 | 0.12(0.01-0.23) |
| Latvia | 184 | -2.14(-2.73-1.54) |  | Republic of Armenia | 184 | 0.12(-0.22-0.46) |
| Portugal | 185 | -2.20(-2.59-1.80) |  | Republic of the Niger | 185 | 0.02(-0.08-0.13) |
| Finland | 186 | -2.34(-2.52-2.15) |  | Northern Mariana Islands | 186 | -0.05(-0.31-0.20) |
| Israel | 187 | -2.44(-2.62-2.27) |  | Republic of Burundi | 187 | -0.07(-0.48-0.34) |
| Austria | 188 | -2.50(-2.75-2.25) |  | Republic of South Africa | 188 | -0.11(-1.02-0.81) |
| Slovenia | 189 | -2.56(-2.85-2.26) |  | Kingdom of Norway | 189 | -0.15(-0.29-0.01) |
| Kazakhstan | 190 | -2.59(-3.67-1.49) |  | New Zealand | 190 | -0.16(-0.26-0.06) |
| Ethiopia | 191 | -2.73(-3.02-2.44) |  | Republic of Poland | 191 | -0.19(-0.69-0.32) |
| Netherlands | 192 | -2.81(-3.28-2.33) |  | Republic of the Union of Myanmar | 192 | -0.28(-0.52-0.03) |
| Rwanda | 193 | -2.87(-3.55-2.19) |  | Republic of Kazakhstan | 193 | -0.31(-0.91-0.30) |
| Saint Kitts and Nevis | 194 | -2.95(-3.83-2.06) |  | Republic of Latvia | 194 | -0.33(-0.70-0.05) |
| Denmark | 195 | -2.96(-3.36-2.56) |  | Kyrgyz Republic | 195 | -0.49(-0.87-0.10) |
| Greenland | 196 | -3.00(-3.43-2.58) |  | Republic of Azerbaijan | 196 | -0.54(-0.90-0.17) |
| Poland | 197 | -3.02(-3.76-2.27) |  | Republic of Estonia | 197 | -0.56(-0.79-0.33) |
| Norway | 198 | -3.06(-3.37-2.75) |  | Saint Kitts and Nevis | 198 | -0.61(-1.05-0.17) |
| Germany | 199 | -3.10(-3.29-2.90) |  | Republic of Tajikistan | 199 | -0.62(-1.09-0.16) |
| Republic of Korea | 200 | -3.22(-3.47-2.97) |  | Hungary | 200 | -0.65(-0.97-0.34) |
| Estonia | 201 | -3.34(-3.75-2.93) |  | Georgia | 201 | -0.66(-1.15-0.16) |
| Hungary | 202 | -3.35(-3.86-2.83) |  | Greenland | 202 | -1.27(-1.57-0.97) |
| Switzerland | 203 | -4.10(-4.37-3.84) |  | Federal Democratic Republic of Ethiopia | 203 | -1.69(-1.93-1.45) |
| Luxembourg | 204 | -4.48(-4.83-4.12) |  | Republic of Rwanda | 204 | -1.72(-2.28-1.17) |

CI-confidence interval; DALY- disability-adjusted life year; EAPC- estimated annual percentage change;

**Table S6.** Deaths- mortality rates- DALYs- and DALY rates of youths and young adults with High BMI (1990–2021) by different age groups

| **Age group** | **Death cases (95%UI)** | | **Death rate/100-000 (95%UI)** | | **DALYs (95%UI)** | | **DALY rate/100-000 (95%UI)** | |
| --- | --- | --- | --- | --- | --- | --- | --- | --- |
|  | **1990** | **2021** | **1990** | **2021** | **1990** | **2021** | **1990** | **2021** |
| 15-19 years | 114.40(56.46-183.40) | 152.11(74.64-240.04) | 0.02(0.01-0.04) | 0.02(0.01-0.04) | 41352.32(19034.66-74746.11) | 70676.90(32191.97-128125.14) | 7.96(3.66-14.39) | 11.33(5.16-20.53) |
| 20-24 years | 3087.96(1949.89-4484.41) | 5768.31(3105.52-8558.06) | 0.63(0.40-0.91) | 0.97(0.52-1.43) | 468440.13(226088.34-756000.67) | 1037160.66(449025.48-1642430.63) | 95.19(45.94-153.63) | 173.68(75.19-275.04) |
| 25-29 years | 6916.12(3903.53-11683.44) | 13852.15(7316.91-22601.35) | 1.56(0.88-2.64) | 2.35(1.24-3.84) | 864947.90(399359.20-1470449.56) | 2000881.90(891401.22-3262516.47) | 195.42(90.23-332.22) | 340.09(151.51-554.53) |
| 30-34 years | 12885.51(6813.17-20917.29) | 27957.30(13958.53-43451.98) | 3.34(1.77-5.43) | 4.63(2.31-7.19) | 1344721.56(594619.01-2235107.04) | 3381096.05(1535904.92-5292987.34) | 348.90(154.28-579.91) | 559.34(254.09-875.63) |
| 35-39 years | 22575.85(11339.44-36723.55) | 47373.07(24506.06-73616.34) | 6.41(3.22-10.43) | 8.45(4.37-13.13) | 2013626.24(874723.28-3364242.67) | 4938616.63(2282505.88-7765650.06) | 571.66(248.33-955.09) | 880.53(406.96-1384.58) |

CI- confidence interval; EAPC- estimated annual percentage change; GBD- Global Burden of Disease;SDI- sociodemographic index; UI- uncertainty interval.

**Table S7.** Deaths- mortality rates- DALYs- and DALY rates of youths and young adults with High BMI (1990–2021) by 2 age groups

| **Age group** | **Death cases (95%UI)** | | **Death rate/100-000 (95%UI)** | | **DALYs (95%UI)** | | **DALY rate/100-000 (95%UI)** | |
| --- | --- | --- | --- | --- | --- | --- | --- | --- |
|  | **1990** | **2021** | **1990** | **2021** | **1990** | **2021** | **1990** | **2021** |
| 15-19 years | 114.40  (56.46-183.40) | 152.11  (74.64-240.04) | 0.02  (0.01-0.04) | 0.02  (0.01-0.04) | 41352.32  (19034.66-74746.11) | 70676.90  (32191.97-128125.14) | 7.96  (3.66-14.39) | 11.33  (5.16-20.53) |
| 20-39 years | 45465.44  (24006.03-73808.69) | 94950.83  (48887.02-148227.73) | 2.88  (1.51,4.67) | 3.96  (2.04,6.18) | 4691735.83  (2094789.83-7825799.94) | 11357755.24  (5158837.5-17963584.5) | 293.95  (130.95,490.41) | 475.40  (215.82,752.15) |

DALY- disability-adjusted life year; UI- uncertainty interval.

**Figure S1.** Comparison of High BMI-related mortality (a) and DALY rates (b) in the 21 regions in 1990 and 2021.


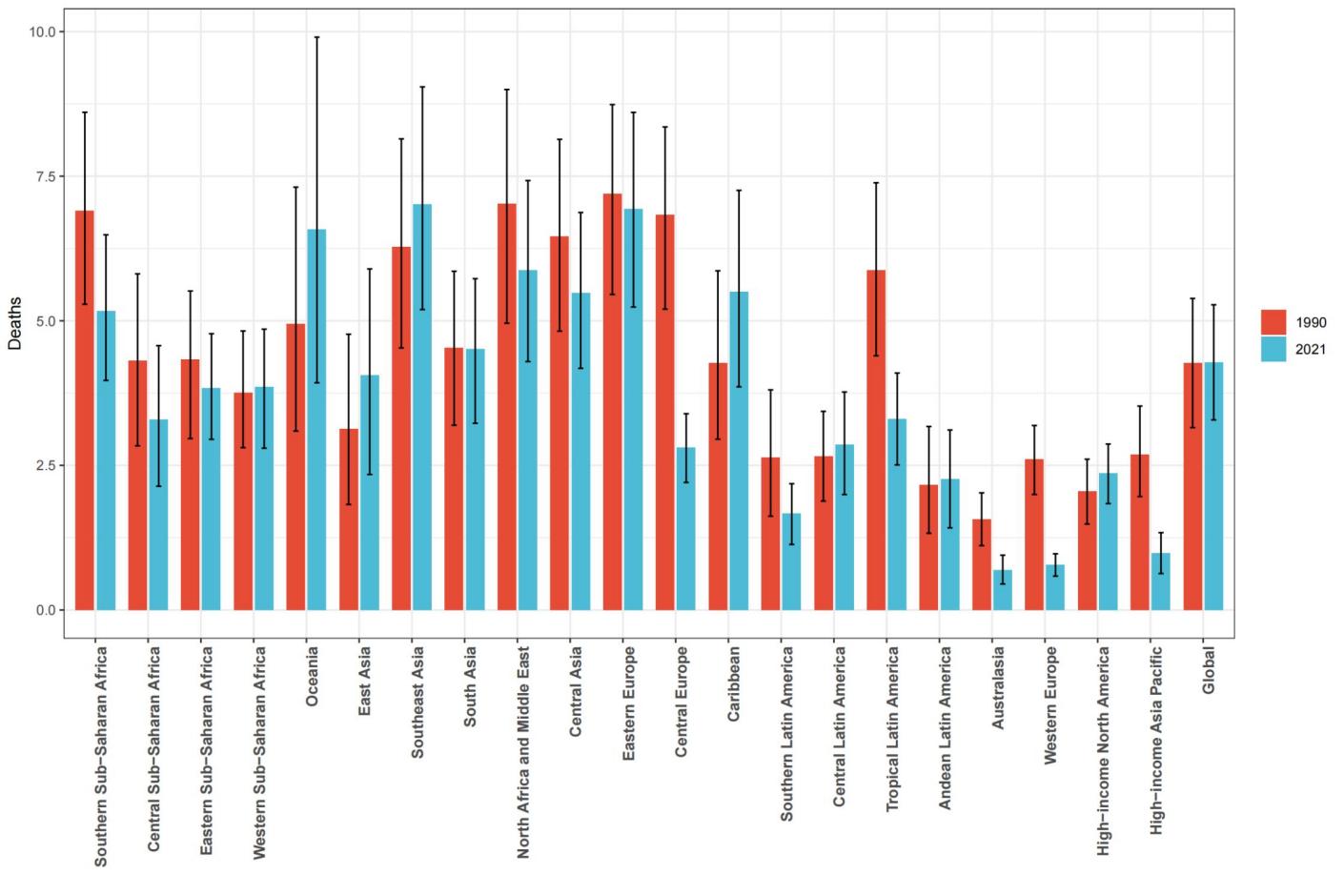

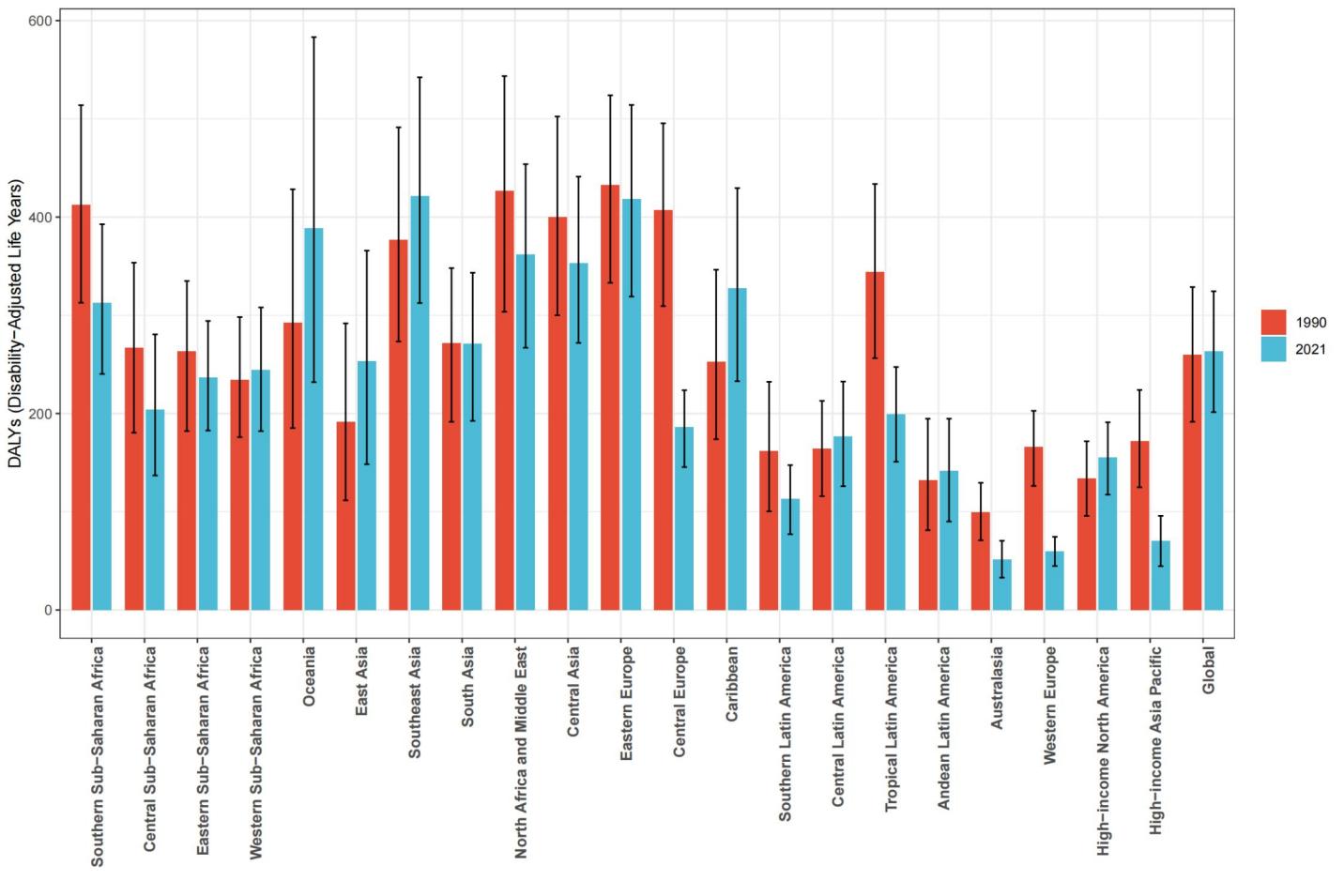


DALYs- disability-adjusted life years; SDI- sociodemographic index.

**Figure S2**. Proportion of High BMI-related disease burden by different age groups


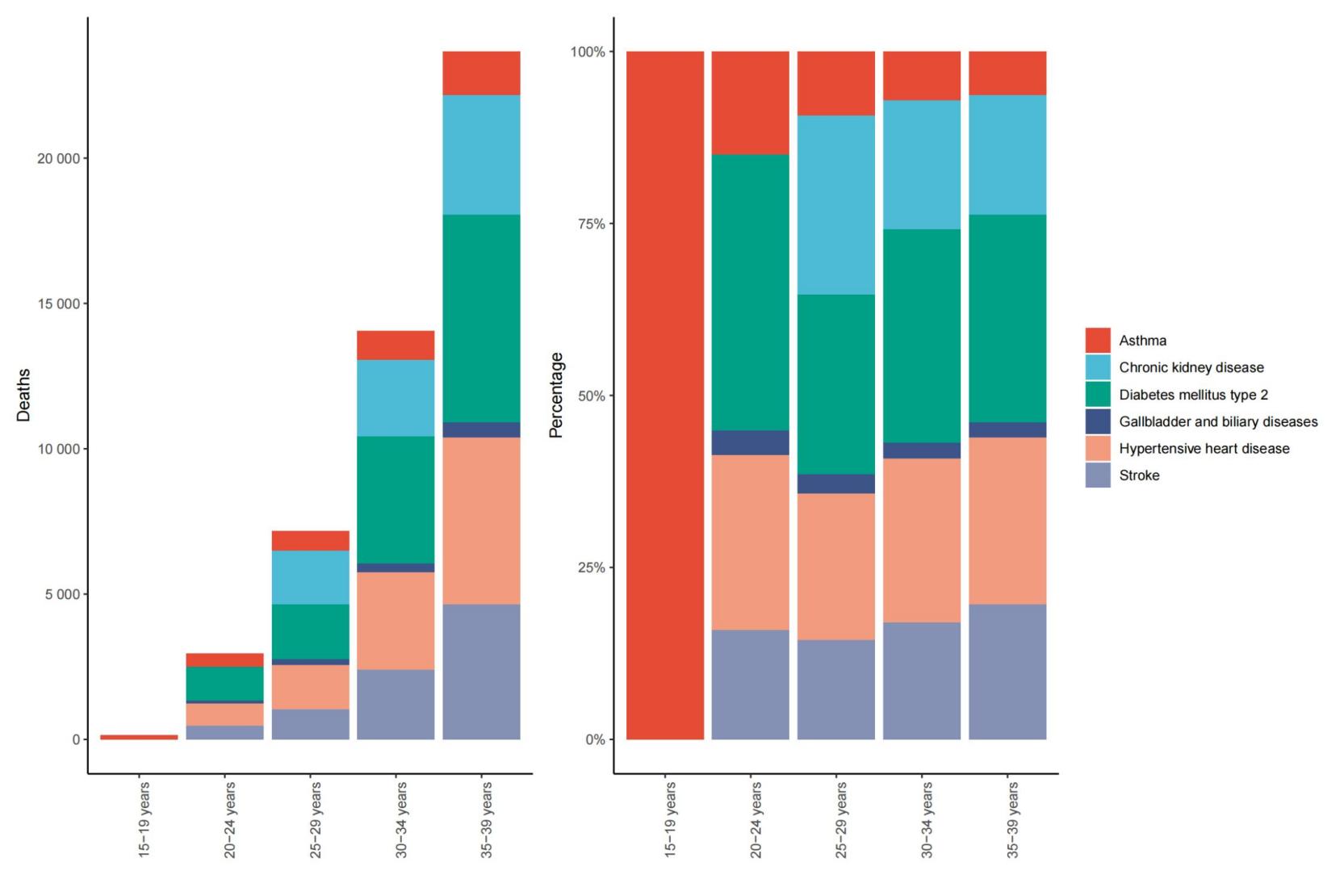

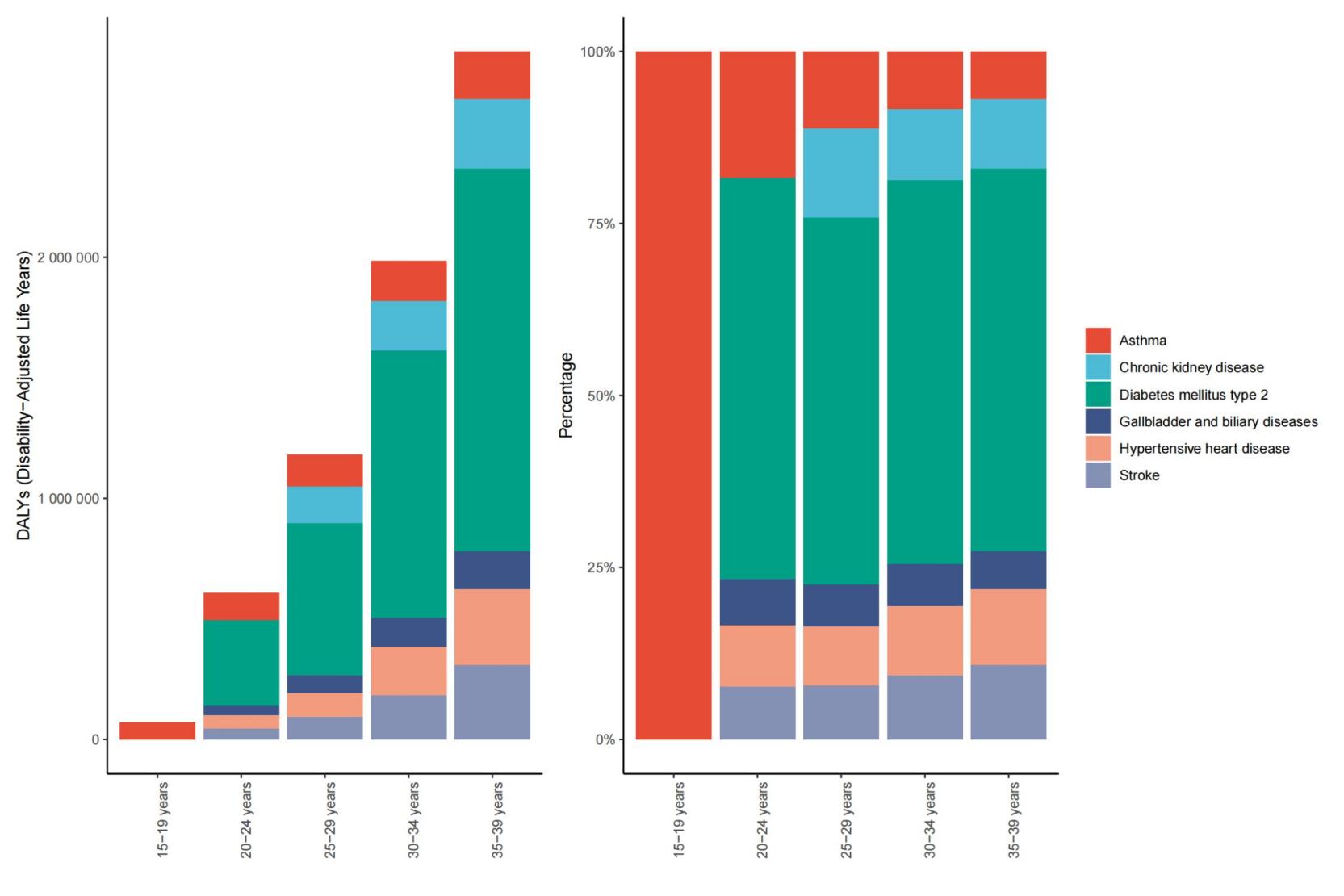


DALYs- disability-adjusted life years;
